# Dietitian-led intervention to manage constipation in Parkinson’s disease: Study protocol for a parallel-group randomized controlled trial (NUTRI-GUT-PD)

**DOI:** 10.1371/journal.pone.0354608

**Published:** 2026-07-27

**Authors:** Juliana Heitich Brendler, Khadija Younes da Silva, Valesca Dall’Alba, Andreza Francisco Martins, Daniel Teixeira-dos-Santos, Artur Francisco Schumacher Schuh, Maira Rozenfeld Olchik

**Affiliations:** 1 Graduate Program in Medical Sciences, Universidade Federal do Rio Grande do Sul, Porto Alegre, RS, Brazil; 2 Department of Nutrition, School of Medicine, Universidade Federal do Rio Grande do Sul, Porto Alegre, RS, Brazil; 3 Nutrition and Dietetics Division, Hospital de Clínicas de Porto Alegre, Porto Alegre, Brazil; 4 Microbiology Unit, Clinical Pathology Service, Hospital de Clínicas de Porto Alegre, Porto Alegre, Brazil; 5 Department of Microbiology, Immunology and Parasitology, Universidade Federal do Rio Grande do Sul, Porto Alegre, RS, Brazil; 6 Neurology Service, Hospital de Clínicas de Porto Alegre (HCPA), Porto Alegre, Brazil [https://ror.org/010we4y38]; 7 Department of Surgery and Orthopedics, School of Dentistry, Universidade Federal do Rio Grande do Sul, Porto Alegre, RS, Brazil; Necmettin Erbakan Üniversitesi: Necmettin Erbakan Universitesi, TÜRKIYE

## Abstract

**Background:**

Constipation is a frequent non-motor symptom in Parkinson’s Disease (PD). Standard care includes dietary and lifestyle guidance or the use of laxatives. Diet represents a promising non-pharmacological approach, but its management in PD is complex and goes beyond simple dietary recommendations. This study protocol aims to examine whether nutritional counseling delivered by a dietitian is more effective than usual care in reducing constipation symptoms in PD patients.

**Methods:**

This 90-day randomized controlled superiority trial uses a parallel-group design. A total of 54 outpatients with PD without dementia and fulfilling the Rome IV criteria for functional constipation will be included. Baseline assessments comprise demographics, nutritional evaluation (anthropometric measurements and sarcopenia indicators), neurological assessment, and the Constipation Scoring System (CSS) scores. Participants will perform stool sample collection for microbiota analysis and will be requested to complete three 24-hour dietary recalls over the subsequent week. After baseline, participants will be randomly allocated to either the control group (usual care) or the intervention group (nutritional counseling delivered by a dietitian). At the first study visit, participants will deliver the stool sample and dietary recalls. The intervention will last for 90 days and will include one in person consultations and bi-weekly follow-up phone calls. Nutritional counseling will address four main topics: healthy eating, food processing, fiber and fluid intake, and levodopa interactions with diet. In addition, participants in the intervention group will receive an individualized diet plan. All baseline assessments will be repeated at the 90-day endpoint. The primary outcome is the change in weekly bowel movement frequency from baseline to day 90, measured through the stool diary. Secondary outcomes include CSS, changes in fecal gut microbiota, macronutrient distribution, diet quality, body composition and sarcopenia indicators. Trial registration number: NCT07213856.

## Introduction

Parkinson’s Disease (PD) has seen a rapid increase in its incidence and prevalence worldwide over the last decades, in an almost pandemic manner [1]. This neurodegenerative disease can span decades and tends to accumulate disabilities over time. For people living with PD, the consequences are significant, and for society, the socioeconomic burden is straining [2]. At this time, there is still no therapy that can slow down disease progression; therefore, optimal treatment should include a multidisciplinary approach with optimized symptomatic pharmacological therapy associated with non-pharmacological strategies [3].

Constipation is one of the most prevalent non-motor symptoms in patients with PD, affecting from 20% to 81% of individuals [4], and contributing to a reduced quality of life [5]. This symptom is linked to alterations in gut motility and gastrointestinal dysregulation [6], which can precede motor symptoms by up to two decades [7], and it is a prodromal characteristic of the disease. The brain-gut axis connects the enteric nervous system, gut microbiota and central nervous system in a communication pathway involving neural, immune, and endocrine mechanisms [8,9]. The prolonged intestinal transit time in these patients can lead to gut microbiota dysbiosis. Dysbiosis in PD is characterized by reductions in beneficial short-chain fatty acid (SCFA)-producing bacteria and elevations in pro-inflammatory microbial species. These alterations are linked to increased intestinal permeability, which allows bacterial endotoxins such as lipopolysaccharides to enter the bloodstream, intensifying neuroinflammation and dopaminergic neuronal degeneration [8,10].

The relationship between gut dysbiosis and constipation in PD is bidirectional and complex. Recent evidence demonstrates that specific gut microbiota alterations may have a causal effect on the development of constipation, and that PD pathophysiology may drive the development of constipation indirectly by altering these specific bacterial taxa [11]. Large-scale metagenomic studies revealed that over 30% of bacterial species, genes, and pathways are altered in PD, with depletion of SCFA-producing genera such as Faecalibacterium and Roseburia, and enrichment of pro-inflammatory taxa including Collinsella and Akkermansia [12,13]. This positions constipation not merely as a symptom but as a potential early biomarker and therapeutic target in PD.

Despite its prevalence and relevance, constipation in PD is often overlooked, and the absence of robust evidence has led to a lack of a standardized approach in international guidelines [14]. Usual care typically relies on general advice regarding fiber intake and lifestyle modifications provided by the clinician, or on the use of laxatives [15]. The available treatment options remain suboptimal for managing constipation symptoms in the majority of patients [16].

Focusing on dietary interventions for constipation in PD, the current literature does not provide sufficient evidence to support strong recommendations. The Mediterranean diet may have beneficial effects on the gut microbiota [17], and has been effective in reducing PD-related constipation symptoms, however those findings were comparable to those achieved with standard care [18]. The Mediterranean approach was associated with increased fiber intake and reduced intestinal inflammation [18], though its feasibility in underdeveloped countries remains a concern. Psyllium fiber supplementation demonstrated positive effects on stool frequency and weight but did not alter colonic transit, this evidence is limited to a small sample of seven participants [19]. Dietary changes in PD patients without expert guidance may pose risks of weight loss, malnutrition, sarcopenia, and worsening abdominal symptoms [20].

Diet management in PD is multifaceted and extends beyond simple dietary advice, encompassing the management of several non-motor symptoms and nutritional complications, including constipation [21]. Nutritional interventions led by dietitians have demonstrated superior effects compared to usual care in randomized controlled trials (RCTs) and meta-analyses, particularly in improving metabolic and cardiovascular outcomes, nutritional status, and diet quality [22–24]. Given the importance of nutritional management in PD and the lack of RCTs evaluating structured dietary interventions in this population, further investigation is warranted. Therefore, the primary objective is to investigate whether individualized nutritional counseling, delivered by a dietitian, is more effective than usual care in increasing the frequency of bowel movements in patients with PD. We hypothesize that the intervention group will present greater improvement in constipation assessments, gut microbiota profile, and diet quality.

## Methods

This protocol is reported following the SPIRIT reporting guidelines.

### Trial design

This study is a randomized, controlled, superiority trial with a parallel-group design and a duration of 90 days. It will be conducted at the Hospital de Clínicas de Porto Alegre (HCPA), Brazil. The study targets adults diagnosed with PD without dementia who are experiencing constipation. Participants will be recruited at the Movement Disorders Research Group outpatient clinic of HCPA.

### Eligibility criteria

Adults with a prior diagnosis of PD, confirmed by a neurologist according to the Movement Disorders Society clinical diagnostic criteria [25], and receiving a stable dose of levodopa for at least 3 months will be included. In addition, participants must report at least two criteria defined by the Rome IV protocol for functional constipation [26]. Exclusion criteria include: (1) atypical or secondary parkinsonism, (2) Hoehn & Yahr stage greater than 3, (3) severe neurological or psychiatric disorders that could interfere with study procedures, (4) dementia diagnosis, (5) diagnosis of active cancer, chronic obstructive pulmonary disease, heart failure, or chronic kidney disease, (6) history of gastrointestinal neoplasms, inflammatory bowel disease, or gastrointestinal surgery (7) constipation secondary to conditions such as hypothyroidism or diabetes mellitus, (8) recent opioid use, (9) use of probiotics or antibiotics within the past 30 days, or within the past 90 days if administered during hospitalization, (10) continuous daily laxative use in the last eight weeks, (11) uses a texture-modified diet, requires feeding assistance, or presents chewing/swallowing limitations, defined as not being classified as FOIS level 7 (total oral intake with no restrictions), (12) enteral or parenteral nutrition, or (13) ongoing nutritional counseling within the last 3 months.

### Recruitment

Participants will be recruited from a database of individuals who participated in a prior study. Individuals meeting the eligibility criteria will be invited to participate in the NUTRI-GUT-PD study, either by phone or during their routine outpatient visits. Those who agree to participate will provide written informed consent, after which the baseline visit will be scheduled to initiate study procedures.

### Randomization

The randomization sequence will be generated by an independent statistician using the Sealed Envelope software. Randomization will be stratified by sex (male/female). Allocation concealment will be ensured through the REDCap electronic data capture tools. Participants will be randomized sequentially in the order that they provide informed consent. Group assignment will be disclosed to participants at D0.

Initial baseline assessments will occur before randomization, eliminating the need for blinding at this stage. Outcome assessors, including dietitians responsible exclusively for assessments and the neurologist performing clinical evaluations at baseline and D90, as well as microbiota sequencing professionals and the statistician, will be blinded to group allocation. Dietitians responsible for delivering the intervention will not be blinded, due to the nature of the counseling.

### Sample size

A sample size of 48 subjects (24 per group) was calculated to determine whether a minimum difference of 1 bowel movement/week exists between the mean weekly bowel movements of the intervention and control groups at the final time point (D90) of this repeated measures study protocol. Accounting for a 10% attrition rate due to potential losses and refusals, the total sample size was adjusted to 54 subjects (27 per group). The calculation considered a power of 80%, a significance level of 5%, and standard deviations of 1.15 and 1.21 bowel movements/week for the intervention and control groups, respectively [18], as well as a 90% retention rate at time points 2 and an exchangeable correlation matrix with a correlation of 0.5. This calculation was performed using the PSS Health online tool [27].

### Outcomes

#### Primary outcome.

The primary outcome of this study protocol is change in weekly bowel movements measured through the stool diary, comparing between-group differences at endpoint (D90).

#### Secondary outcomes.

Secondary outcomes will evaluate the following from baseline (D0) to the end point (D90):

- Proportion of participants achieving a clinically meaningful improvement, defined as an increase of >1 bowel movement per week;
- Changes in total scores of the Constipation Scoring System (CSS);
- Alterations in fecal gut microbiota composition, specifically measuring alpha diversity (within-sample richness), beta diversity (between-sample composition), and differential abundance at the species level;
- Changes in macronutrient distribution derived from three 24-hour dietary recalls;
- Changes in the Screener-Nova scores, regarding food processing;
- Changes in Bioelectrical Impedance Analysis (BIA) and anthropometric measurements;
- Changes in sarcopenia assessments.

### Participant timeline

**Fig 1:**
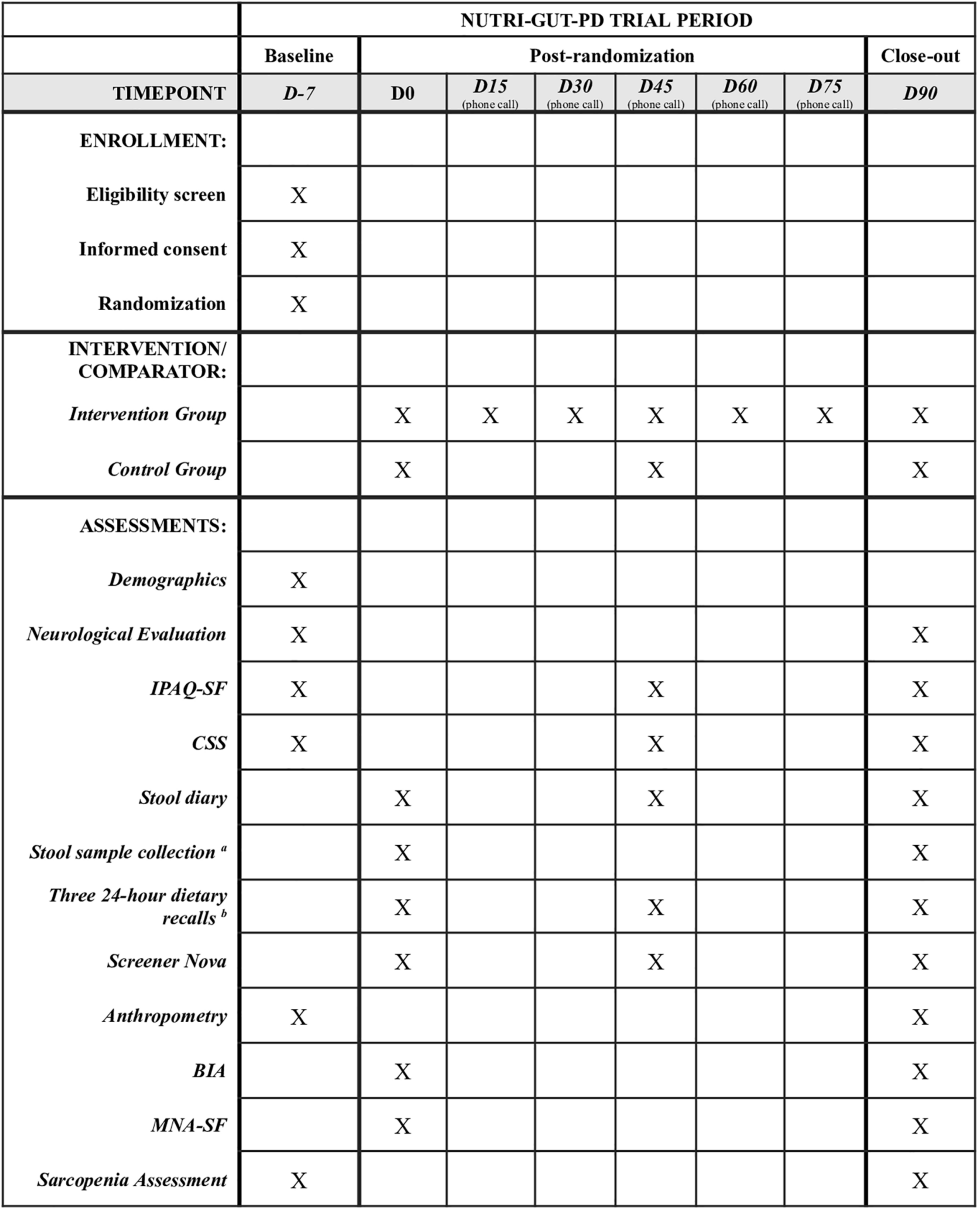
Participant schedule of enrolment, interventions, and assessments. IPAQ-SF: International Physical Activity Questionnaire- Short Form; CSS: Constipation Scoring System; FFQ: Food Frequency Questionnaire; BIA: Bioelectrical Impedance Analysis; MNA-SF: Mini Nutritional Assessment – Short Form. *All visit days are ± 3 days. ^a^ Patients will receive stool collection kits one week before visit days, allowing them to bring the samples on the visit day for analysis. ^b^ Two 24-hour dietary recalls by phone in the week prior and one in-person recall at the study visit.

The participant timeline is presented in Fig 1. After eligibility screening and informed consent, participants will complete baseline assessments and will then be randomized to either the control or the intervention group. The intervention period will last 90 days.

At the time of submission, the study is in the pre-recruitment phase. Participant recruitment is expected to begin in March 2026 and to be completed by July 2026. Each participant will undergo a 3-month intervention period, and data collection is expected to be completed by November 2026. Data analysis is planned between December 2026 and January 2027, and study results are expected in early 2027. Study findings will be disseminated through peer-reviewed publications and conference presentations.

### Data collection

**Fig 2:**
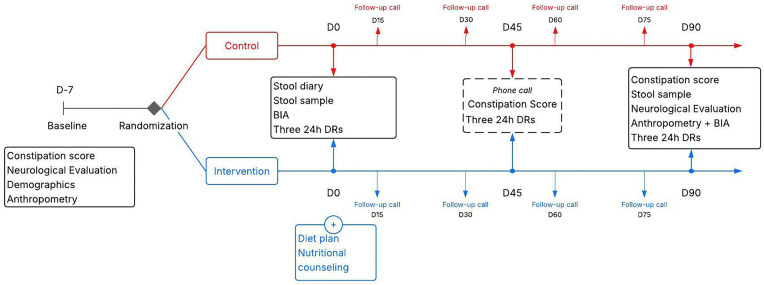
Study Design NUTRI-GUT-PD. BIA: Bioelectrical Impedance Analysis; 24h DRs: Three 24h Dietary Recalls. Figure created by the author (2025).

The study will include three in-person visits at baseline, D0, and D90, with a phone contact on D45. In addition, participants will receive phone calls every 15 days to promote adherence to the intervention, address potential issues, and inquire about intestinal symptoms. The assessments planned for each visit are summarized in Fig 2 and detailed below.

Considering the complexity of the proposed assessment battery and the inherent mental and physical fatigue associated with the neurodegenerative condition, several strategies will be utilized to promote participant retention and adherence throughout the trial. Participants will be offered a ± 3day window for all scheduled visits and phone calls. Caregivers will be actively encouraged to attend study visits and assist with data collection, and provide logistical support to the participant. Lastly, to facilitate compliance, scheduled communication via text message or telephone calls will be established. Encompassing confirmation of visit appointments, reminders regarding BIA orientation, daily prompts to complete the stool diary, and reminders to collect and bring the stool sample and the completed diary to the scheduled visit.

#### Sociodemographic data.

A standardized questionnaire including age, sex, education, self-reported ethnicity, and household income will be used to collect sociodemographic data at baseline.

#### Neurological evaluation.

Medical history, covering disease duration, age at diagnosis, comorbidities, and use of Deep Brain Stimulation (DBS), will be collected at baseline. Disease severity and duration will be assessed using the Hoehn and Yahr staging scale. Information on medication use will be collected at baseline and on D90, including the daily levodopa equivalent dose (LEED) and any concomitant treatments or constipating medications.

The study neurologist will perform a clinical assessment at baseline and D90, including the following validated instruments:

- MDS-Unified Parkinson’s Disease Rating Scale (MDS-UPDRS) [28]: Comprehensive assessment of PD severity across motor and non-motor symptoms. The MDS-UPDRS consists of four parts with a total of 65 items, each rated on a 5-point scale (0 = normal, 1 = slight, 2 = mild, 3 = moderate, 4 = severe).
- Montreal Cognitive Assessment (MoCA) [29]: A cognitive screening that evaluates attention, executive functions, memory, language, visuospatial abilities, abstraction, calculation, and orientation. The total score ranges from 0 to 30, with higher scores indicating better cognitive performance. The cut-off score of ≤25 will be used to indicate possible cognitive impairment.
- Swallowing Disturbance Questionnaire (SDQ) [30]: A 15 items screening questionnaire designed to identify symptoms of dysphagia. The instrument assesses the frequency of swallowing-related difficulties in daily life, with higher scores indicating greater severity of swallowing disturbances.
- International Physical Activity Questionnaire- Short Form (IPAQ-SF) [31]: A 7-item instrument that assesses physical activity over the last 7 days. Results are calculated in MET-minutes/week by multiplying the activity’s MET by its duration and frequency. Based on these scores, individuals are classified into three levels: Low (failing to meet other criteria), Moderate (total from any activity ≥600 MET-min/week or specific frequency thresholds), and High (vigorous activity reaching ≥1500 MET-min/week or a total of ≥3000 MET-min/week). This assessment will be repeated at mid-point (D45) through phone contact by the study dietitian.

#### Constipation assessment.

Constipation will be evaluated using two instruments to capture both subjective and objective aspects of bowel function. All participants will receive a stool diary to be completed daily. This tool will be used to monitor the progression of constipation symptoms throughout the study period. The following items will be recorded:

- Was evacuation present today?
  - Weekly bowel movement frequency will be calculated accordingly.
- What was the stool consistency?
  - Assessed using the Bristol Stool Form Scale. Types 1–2 will be classified as hard stools and reported as the weekly percentage of total evacuations.
- Was there a sensation of straining?
  - Responses (“Yes” or “No”) will be summarized as weekly percentages.
- Was the evacuation incomplete?
  - Responses (“Yes” or “No”) will be summarized as weekly percentages.
- Did you use laxatives today due to the absence of spontaneous bowel movements?
  - The weekly frequency of rescue laxative use will be calculated.

The CSS will be administered at baseline, D45, and D90. This instrument assesses intestinal dysfunction through eight items evaluating bowel movement frequency, straining, incomplete evacuation, and time spent in the bathroom. The total score ranges from 0 to 30, with higher scores indicating a worse perception of functional constipation. The culturally adapted Brazilian version has demonstrated strong comprehension and psychometric reliability [32]. Intermittent or rescue laxative use will not be restricted or managed by the research team during follow-up to ensure patient safety and ethical care, however this information will be accounted for as a potential confounder in the statistical analysis.

#### Gut microbiota.

At baseline, participants will receive standardized instructions and two sterile containers labeled with their name and the collection date, to collect stool samples at home on D0 and D90. Participants will be reminded during the visit and through follow-up messages about the instructions for proper at-home stool sample collection.

Samples must be stored refrigerated (up to 48h) and delivered to the research team, preferably within 1 hour of removal from refrigeration [33,34]. Upon receipt at the laboratory, samples will be aliquoted into sterile microtubes and stored at –80 °C until analysis.

- DNA Extraction: The DNA will be extracted from 250 mg of the sample using QIAamp PowerFecal Pro DNA Kit following the manufacturer’s protocol. DNA yield and quality will be verified before sequencing.
- 16S rRNA Sequencing: The V3–V4 hypervariable region will be amplified by PCR using the Illumina® 16S Metagenomic Sequencing Library Preparation protocol. Amplified products will be purified with AMPure XP beads, quantified using Qubit DNA HS assays, and fragment distribution assessed with the TapeStation 4200 system. Libraries will be pooled and sequenced on the MiSeq® platform with the MiSeq™ i100 Series 25M Reagent Kit (600 cycles). A negative control (molecular-grade water) will be included in each run.

#### Dietary assessments.

Before each assessment point (D0, D45, and D90), participants will complete three 24-hour dietary recalls. The 24-hour dietary recall requires participants to provide a detailed account of all foods and beverages consumed during the preceding 24-hour period. Two recalls will be conducted by telephone during the week preceding the visit, and the third will be completed in person at the visit, referring to the day immediately before. To ensure reliable estimation of most nutrients, the recalls will be collected on nonconsecutive days, including 2 weekdays and 1 weekend day [35]. Standardized multiple-pass methodology will be used: quick listing of foods, detailed descriptions, portion size estimation with household measures, checking for forgotten items (oil, condiments, snacks), and final review. Nutrient intake, including energy, macronutrients, micronutrients, and fiber, will be analyzed using the DietBox© software. Calculations will be based on data from the Brazilian Food Composition Table (TACO) and the IBGE Household Budget Survey (POF 2008–2009) tables.

To evaluate diet quality the Screener-NOVA [36] will be implemented. It is a brief, web-based questionnaire designed to assess diet quality according to the NOVA food classification system. It consists of seven yes/no sections addressing the consumption of unprocessed or minimally processed foods (fruits, vegetables, legumes, grains) and ultra-processed foods (sugar-sweetened beverages, ready-to-eat meals, snacks). The questionnaire will be administered at D0, D45, and D90 with support from the dietitian. It generates two indicators: the NOVA-WPF (Whole Plant Foods) score, ranging from 0 to 33 points, where higher values indicate greater consumption of WPF; and the NOVA-UPF (Ultra-Processed Foods) score, ranging from 0 to 23 points, where higher values indicate greater consumption of UPF. For classification, a NOVA-WPF score of ≥ 10 points will be used to define desirable consumption, and a NOVA-UPF score of < 4 points will be used to define non-elevated consumption [37].

#### Nutritional status.

At baseline and D90, anthropometric data will be collected according to the national guidelines [38]. This includes weight and height for BMI calculation and classification according to the World Health Organization [39] categories. To screen for low muscle mass, we will measure calf circumference (<34 cm for males, < 33 cm for females) [40] and arm circumference (<28 cm for males, < 25 cm for females) [41]. Finally, high abdominal adiposity will be assessed via waist circumference (>102 cm for men, > 88 cm for women) and waist-hip ratio (>0.9 for men, > 0.85 for women) [42].

BIA with multiple electrodes will be performed in the first (D0) and last visit (D90). It estimates lean mass, fat mass, fat percentage, cellular mass, total body water, basal metabolic rate, segmental distribution and visceral fat. Participants will follow standardized pre-test conditions (≥ 2 to 4h fasting, empty bladder, no intense physical activity for 24h, no alcohol/caffeine the previous day).

The Mini Nutritional Assessment – Short Form (MNA-SF) [43] will be applied by a trained dietitian at D0 and D90 of the study to assess malnutrition risk. Scores classify participants as well-nourished (12–14), at risk of malnutrition (8–11), or malnourished (0–7).

#### Sarcopenia assessment.

Sarcopenia assessment will be done in the baseline visit and also at the end point of the study (D90). It will follow the revised 2019 European consensus [44]:

- Screening: SARC-F questionnaire (≥4 indicates probable sarcopenia).
- Muscle Strength: Handgrip strength via calibrated dynamometer (<27 kg men; < 16 kg women = low strength).
- Muscle Mass: Appendicular skeletal muscle mass (BIA; < 20 kg men, < 15 kg women = low mass).
- Physical Performance: Timed Up and Go (TUG) test; ≥ 20 s indicates poor performance.

### Interventions

#### Intervention group.

The nutritional intervention is aimed at improving diet quality, constipation symptoms, and gut health. The intervention is based on recommendations from the Brazilian and European consensus for neurological diseases [45,46] and the Food Guide for the Brazilian Population [47]. Social Cognitive Theory (SCT) is the theoretical basis of nutritional counseling, which defines that dietary behavior results from reciprocal interactions between personal factors, environmental influences, and behavior [48]. The intervention will target key SCT constructs, including self-efficacy (confidence in one’s ability to make and maintain dietary changes), self-regulation (goal setting, self-monitoring, and problem-solving), environmental restructuring (modifying food purchasing and preparation contexts), and social support (interpersonal influences on eating behaviors) [49].

The intervention will last 90 days, with an initial in-person visit (D0) at the research center followed by biweekly telephone contacts until the final assessment, totaling one onsite visit and five phone calls between the participant and the study dietitian. Participants in the intervention group will receive individual nutritional counseling sessions, a standardized dietary intervention manual on the covered topics and a personalized diet plan elaborated by a dietitian specialized in neurology and elderly health. Visit outlines and decision algorithms will guide each session and diet plan prescription, ensuring consistent application of the intervention protocol, while allowing flexibility for individual characteristics. Details regarding the dietitian’s training, sessions outlines, and monitoring are provided in S2 Appendix.

During the first intervention session (D0), the dietitian will assess participants’ prior knowledge and provide counseling on four key topics: levels of food processing, healthy eating, the importance of fiber and fluid intake, and interactions between levodopa and nutrients (Table 1). Individual goals will be established collaboratively with each participant during this session and reinforced throughout the study period (e.g., increasing daily fruit intake, reducing added sugar in coffee, or replacing ultra-processed foods with minimally processed alternatives). This section of the visit will range from 60 to 90 minutes. Participants will also receive a Dietary Intervention Manual (S2 Appendix) developed specifically for this trial, which was reviewed by faculty members from the fields of nutrition, medicine, speech therapy, and pharmacy. The manual covers the four key topics to ensure consistency in the information provided across the group.

**Table 1:** Nutritional counseling framework for the intervention.

Nutritional Counseling Topics	Description
Food Processing	- Definition and examples of minimally processed, processed, and ultra-processed foods. - Practical identification of UPFs in daily life.
Healthy Eating	- Preference for natural or minimally processed foods. - Moderate use of oils, fats, salt, and added sugars. - How to assemble a balanced plate. - How to read food labels.
Fiber and Fluid Intake	- Types of dietary fiber (soluble and insoluble) and their natural food sources. - Role of fiber in improving constipation and supporting gut microbiota. - Importance of adequate hydration and strategies to increase daily fluid intake.
Levodopa and Nutrients	- How levodopa absorption interacts with dietary protein. - Identification of protein-rich foods and recommended intake. - Meal-time strategies to minimize levodopa–protein interaction.

Source: Author, 2026.

During the same visit, participants will receive a personalized diet plan, followed by a 30-minute explanation on how to implement it. Based on the baseline dietary assessment, the individualized diet plan will be previously designed according to protocol and each participant’s food and cultural preferences within the Brazilian context. The plan will outline a full day of meals (breakfast, lunch, afternoon snack, dinner, and supper) with portion sizes provided in grams and standard household measurements. Each meal will include food options.

**Fig 3:**
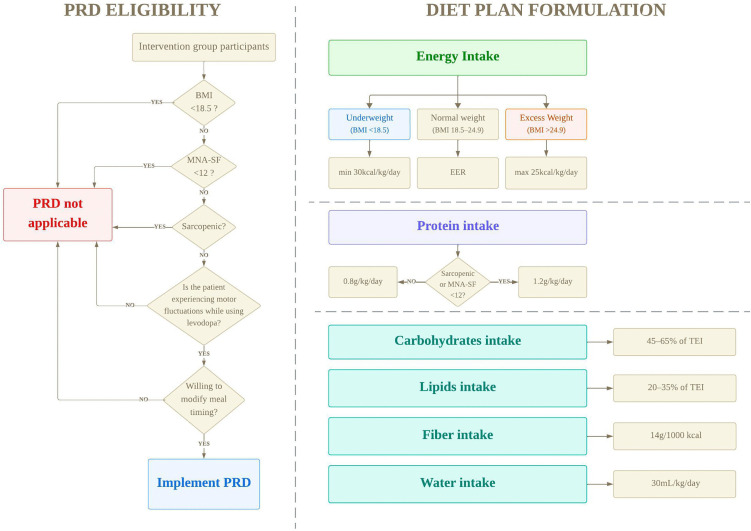
Diet Plan Formulation Diagram. BMI: Body Mass Index; EER: Estimated Energy Requirement; MNA-SF: Mini Nutritional Assessment – Short Form; PRD: Protein Redistribution Diet; TEI: Total Energy Intake. Figure created by the author (2026).

To ensure consistency among all study dietitians, decisions regarding individual dietary needs will be guided by the diagram presented in Fig 3. The nutrient distribution will follow these principles: (1) energy intake estimated using the Estimated Energy Requirement (EER) [50], (2) protein intake is set to 0.8 g/kg/day and Protein Redistribution Diet (PRD) prioritized when appropriate, (3) carbohydrates composing 45–65% of total energy intake, (4) lipids composing 20–35% of total energy intake, and (5) fiber intake set at a minimum of 14 g/1000 kcal. For participants classified as sarcopenic, malnourished or at risk of malnutrition in baseline assessment, protein intake will be increased to 1.2 g/kg/day and PRD will not be implemented [51]. For participants classified as overweight (BMI ≥ 25 kg/m²), the energy intake will have a target of 25 kcal/kg/day. And for participants classified as underweight (BMI 18.5 kg/m²), minimum energy intake will be set at 30 kcal/kg/day or higher as needed to promote weight gain and ensure adequate nutritional status.

Food selection will emphasize items aligned with the counseling recommendations, prioritizing minimally processed and natural foods, while restricting ultra-processed products and high-sugar foods. PRD will be recommended for participants experiencing motor fluctuations while using levodopa, when feasible and safe. PRD is defined as limiting protein-rich foods at breakfast and lunch, with higher protein offerings at dinner and supper to meet total daily protein requirements. This strategy has been associated with improvements in motor fluctuations and lower levodopa requirements [52]. Fiber intake from fruits, vegetables, and whole-grain cereals will be emphasized, alongside adequate hydration. Fluid intake will follow a target of 30 mL/kg/day.

Nutritional counseling, dietary goals, and diet plan implementation will be reinforced every 15 days through phone calls with the study dietitian, totaling five sessions. All four counseling topics will be reviewed during each call, and participants will have the opportunity to discuss any difficulties or questions related to the diet plan.

At midpoint (D45), three 24-hour dietary recalls and diet quality will be analyzed. Individual goals, nutrient composition, and overall diet quality will be reviewed. Adherence to the diet plan will be defined as meeting at least 75% of the nutrient goals. If participants withdraw or fail to fully comply, primary outcome data will still be collected whenever possible to support an intention-to-treat analysis.

#### Control group.

Participants in the control group will maintain their “Treatment as Usual”, defined as the standard clinical care provided by their independent neurologist. This may include lifestyle advice (adequate hydration, dietary fiber intake, and physical activity) as well as over-the-counter laxatives, when clinically indicated. To ensure standardization of the control variables, participants will record every evacuation and the specific use of any rescue laxatives in their stool diary. Participants will receive follow-up calls every 15 days to maintain engagement and monitor for any new clinical recommendations provided by their neurologists. During these calls, researchers will strictly adhere to a non-interventional script, abstaining from providing any nutritional counseling or dietary adjustments (S2 Appendix). At the end of the study, if proven beneficial, the control group will be offered the same nutritional counseling provided to the intervention group (wait-list design).

### Statistical analysis

Participants will be identified by numeric codes to ensure confidentiality. All collected data will be collected and managed using REDCap electronic data capture tools hosted at *Universidade Federal do Rio Grande do Sul* [53], accessible only to the responsible investigators.

Descriptive statistics will be presented according to variable type: mean and standard deviation (or median and interquartile range) for continuous variables, and absolute frequencies and percentages for categorical variables.

Intent-to-treat (ITT) analyses will be performed for all outcomes. Primary and secondary outcomes will be analyzed using generalized linear mixed models (GLMM) with group, time, and their interaction as fixed effects, and random intercepts by participants. Age, sex, disease duration, and LEED will be included as baseline covariates in all models. Weekly laxative use will be included as a time-varying covariate to adjust for its potential confounding effect on bowel movement frequency. Missing data will be handled through the likelihood-based estimation inherent to GLMM, which provides valid estimates under the missing at random (MAR) assumption. Sensitivity analyses will be conducted to assess the robustness of results to potential departures from the MAR assumption.

For gut microbiota analysis, alpha-diversity will be calculated using the Shannon index, and statistical significance will be assessed using linear mixed-effects models. Beta diversity will be examined by Principal Component Analysis (PCA), Principal Coordinates Analysis (PCoA), and Non-metric Multidimensional Scaling (NMDS), based on Aitchison and Jaccard distances. Statistical significance and explained variance will be assessed using Permutational Multivariate Analysis of Variance (PERMANOVA) and linear mixed-effects models. Differential abundance analysis will be performed using MaAsLin2 (Multivariable Association with Linear Models 2). Multiple testing corrections will be applied using the Benjamini–Hochberg method to control the false discovery rate (FDR), and a mixed directional FDR (mdFDR) approach with Holm’s procedure to control the familywise error rate. Statistical significance will be set at a two-sided α = 0.05. Analyses will be performed using Python version 3.6.9 with Pandas v. 1.2.5 and SciPy v. 1.7.0.

### Ethical considerations

This project has been approved by the Research Ethics Committee of Hospital de Clínicas de Porto Alegre under protocol number 2025−0374 and registered in ClinicalTrials.gov under NCT07213856. This is protocol version 1.0, dated January 2026. Researchers declare compliance with the Declaration of Helsinki and the General Data Protection Law (*Lei Nº 13.709*, August 14, 2018) regarding the handling of personal and sensitive data. Participants will be recruited for the present study from a database of individuals who previously participated in a study approved by the Research Ethics Committee of the HCPA, under CAAE: 25047219.0.2002.5327, and authorized under approval number 2021−0576.

De-identified individual participant data and statistical code will be made available upon reasonable request after results publication, subject to ethical approval and data protection regulations. All potential risks and benefits are described in the ICF, and participants will provide specific consent for stool sample collection and microbiota analysis.

Potential adverse events related to dietary changes (e.g., bloating, abdominal discomfort, unintended weight loss) will be monitored systematically during follow-up calls and visits. Given the low-risk behavioral nature of the intervention, no Data Monitoring Committee was established. Trial conduct will be monitored by the principal investigators through regular team meetings.

All data will be anonymized, especially during statistical analysis. After the trial, if the intervention demonstrates benefit, participants in the control group will be offered the same nutritional counseling as a matter of ethical fairness. Additionally, all participants will be able to request feedback on their individual questionnaire and assessment results.

The funder had no role in the study design, data collection, analysis, interpretation of data, or decision to submit the manuscript for publication. The trial is coordinated by the principal investigators, and no independent steering committee or endpoint adjudication committee was established due to the low-risk nature of the intervention.

## Discussion

This study is the first randomized controlled trial identified to date to evaluate the effect of a dietitian-led nutritional intervention for managing constipation in patients with PD. Evidence for nutritional interventions on PD constipation and gut health are scarce and generally focus on a specific dietary pattern or a supplement [18,54–56]. Considering the heterogeneity of the literature on this topic, recommendations have been pointing to a minimally processed plant-based diet with dietary counseling tailored to personal preference, cultural context, and socioeconomic realities [57].

By testing a structured, individualized nutritional intervention, this trial directly addresses this gap in evidence-based management of constipation in PD. Dietitian-led interventions have demonstrated superior improvements compared to usual care or dietary advice provided by other health professionals in the management of prediabetes [23], diabetes type 2 [22] and hypertension [24]. In PD, only 11–15% of patients have access to dietetic care, which calls for further research in this area [21]. The trial is also designed to investigate a range of secondary outcomes, including changes in gut microbiota composition, dietary intake and quality, body composition, and markers of sarcopenia. Particularly, gut microbiota have presented changes in beta-diversity as rapidly as 24 hours after a change in dietary pattern, and genus-level changes in microbiota have been seen after a 3-month intervention period [58,59], although species-level changes may not be apparent within this time frame. Investigating these outcomes will provide more consistency to the importance and influence of diet in PD management.

If successful, this trial will contribute robust evidence to support the integration of specialized nutritional counseling into the standard of care for PD. Such integration has the potential not only to improve quality of life but also to advance precision nutrition approaches that consider the interplay between microbiota, diet, and disease progression. The findings could strengthen clinical guidelines and reinforce the inclusion of nutrition as a key component of multidisciplinary care in PD.

This trial has some limitations. First, restricting eligibility to patients with a maximum H&Y stage of 3 limits generalizability to individuals with more advanced PD. However, this criterion is necessary to ensure adherence and completion of assessments, as more severe disease may compromise participation. Although constipation is also common in later stages, nutritional management must be individualized by disease severity, which already limits broad generalization. Second, as with any behavioral intervention, complete uniformity of counseling sessions is difficult to guarantee. To address this, the diet manual and individualized diet plan were standardized to enhance fidelity. Accordingly, due to the nature of the nutritional intervention, participant blinding was not feasible, as active awareness is essential for adherence to the dietary plan. While the lack of blinding may introduce performance bias, we mitigated potential detection bias by ensuring that researchers responsible for endpoint assessments and data analysis remained blinded to group allocation. Home stool sample collection may also affect microbiota analysis due to variations in storage and transport, but requesting in-center collection would be impractical for constipated patients; therefore, clear instructions will be reinforced during visits and follow-ups. Despite these limitations, the protocol was designed to maximize internal validity through rigorous standardization and fidelity monitoring.

## Supporting information

S1 Appendix SPIRIT checklist. (PDF)

S2 Appendix Dietary intervention manual and visits outlines. (PDF)

S3 Appendix Protocol approved by the ethics committee (English translation). (PDF)
