# Supplementary material for: Dietitian-led intervention to manage constipation in Parkinson’s disease: Study protocol for a parallel-group randomized controlled trial (NUTRI-GUT-PD)
Source: PLoS One. 2026 Jul 27;21(7):e0354608. doi: 10.1371/journal.pone.0354608 (PMC13405298; doi:10.1371/journal.pone.0354608)
Supplement: S2 Appendix — (PDF) [file pone.0354608.s002.pdf]

Part A. Dietary Intervention Protocol training

To ensure standardization of the dietary intervention across studies [1–3], all participating dietitians will undergo a training program. Initial training includes a comprehensive presentation of the intervention protocol and the dietary intervention manual. Followed by a presentation on key components of the Social Cognitive Theory (SCT): self-efficacy, self-regulation, environmental restructuring, and social support [4]. Dietitians will then receive technical instruction on using the *Dietbox©* nutritional software and configuring it to use the Brazilian Food Composition Table (TACO) and the IBGE Household Budget Survey (POF 2008-2009) tables.

Training will progress to counseling simulations using standardized patient scenarios. These scenarios are designed to reflect the diversity of the study population, including: varying stages of disease progression (Hoehn and Yahr 1-3), different educational levels, and prior nutritional health knowledge. Finally, before participating in the trial, each dietitian will complete a competency assessment conducted by the Principal Investigator (PI). This involves a role-play scenario where the dietitian must demonstrate adherence to the protocol and effective communication strategies.

To monitor fidelity throughout the study, 20% of initial visits and phone follow-ups will be randomly audited. The PI will accompany these sessions in person to assess protocol adherence and provide feedback to the dietitians. Concomitantly, weekly team meetings will occur to discuss challenges encountered during intervention sessions and ensure ongoing consistency in counseling techniques across the entire team.

Part B. Structured Session Protocols

Considering the nature of the nutritional intervention, certain aspects such as goal setting and diet plans must be individualized to each participant's characteristics. However, to ensure standardized delivery while maintaining flexibility, flowcharts and scripts will be used to structure the session outlines. The sessions will follow the structure of the Dietary Intervention Manual (Part D) chapters. They will incorporate open-ended questions to assess participants' existing knowledge and comprehension of the topics presented. Outlines for the first visit and the subsequent phone follow-up are provided below:

INTERVENTION GROUP

1st Session *(in person - 60 to 90 minutes total)*

Introduction (5 min)

- Welcome participant, and introduce yourself
- Ask permission to proceed: *"Is it okay if we discuss your current eating habits and how we might work together to improve your nutrition?"*
- Inform expected timeframe: *"This section of the visit will take from 60 to 90 minutes, ok?"*

Assess Current Knowledge & Readiness (5 min)

- *"What do you already know about how diet affects Parkinson's disease?"*
- Readiness ruler (0-10): *"On a scale of 0-10, how ready are you to make changes to your diet?"*
  - **If 0-3 (Not Ready)**: Focus on education, avoid pushing for immediate goals
  - **If 4-7 (Unsure)**: Explore ambivalence, discuss benefits and barriers

- **If 8-10 (Ready)**: Move quickly to goal setting and action planning

Nutritional Counseling + Dietary Intervention Manual (30-40 min)

For each topic: Provide information → Ask teach-back question → Address concerns

1st Principle: Food Processing (7-10 min)

- Define and provide examples of minimally processed, processed, and ultra-processed foods.
- Show examples from the manual.
- Explain how to read food labels and their importance for choosing food items
- Open-ended questions:
  - *"Can you give me an example of an ultra-processed food you eat regularly?"*
  - *"What are your favorite whole-plant or minimally processed foods?"*
  - *"Did you already know how to interpret food labels?"*

2nd Principle: Healthy Eating (7-10 min)

- Discuss briefly the 10 steps for healthy eating
- Focus specifically on 1-4
- Demonstrate how to achieve a balanced plate with images from the manual
- Open-ended questions:
  - *"Do you think it is feasible in your reality to include more whole plant foods in your diet?"*
  - *"What challenges do you think you might face in reducing ultra-processed foods?"*

- *"If you were making lunch today, what would a balanced plate look like for you?"*
- *"In your household, who is responsible for buying food and preparing meals?"*

<u>3rd Principle: Fiber and Fluid Intake (7-10 min)</u>

- Explain what the types of fibers are (soluble and insoluble)
- Explain where to find natural sources
- Discuss the role in constipation management and gut health
- Inform the individual of the hydration target (30 mL/kg/day)
- Open-ended questions:
    - *"What are some fiber-rich foods you already enjoy or would be willing to try?"*
    - *"How do you currently measure your hydration?"* → propose realistic schemes to increase daily hydration if needed

<u>4th Principle: Levodopa and Nutrients (7-10 min)</u>

Identify previously if PRD is appropriate for this participant

- Explain the levodopa-protein interaction
- Explain how this interaction affects medication efficacy and motor symptoms
- Identify protein-rich foods
- Discuss meal-timing strategies
- Open-ended questions:
    - *"In your own words, how might protein timing affect your medication?"*
    - *"Do you think changing the protein timing would be challenging for you?"*

Collaborative Goal Setting (7-10 min)

- Review participants' food preferences and cultural patterns
- Identify 2-3 specific, achievable goals
- Open-ended question: *"Based on what we've discussed, what is ONE change you feel confident making this week?"*
- Document goals in the manual
- Examples of appropriate goals:
  - "Add one serving of fruit a day on at least 5 days/week."
  - "Replace sugary beverages with water at lunch."
  - "Choose whole-grain bread instead of white bread."

Diet Plan Review (30 min)

- Present personalized diet plan *(previously prepared)*
- Review each meal with portion sizes and meal options
- Explain the rationale for nutrient targets specific to the participant
- Address questions and concerns
- Discuss practical implementation strategies
- Open-ended question: *"What part of this plan seems most challenging for you?"*
- Problem-solve barriers together

Closing (5 min)

- Summarize key points and goals
- Provide a standardized manual
- Schedule next telephone contact (D15)
- Final question: *"What questions do you have before we finish today?"*

INTERVENTION GROUP

2nd to 6th Sessions *(remote - 15 to 20 minutes total)*

OPENING (2 min)

- Greeting: *"Hello! This is the dietitian from the study NUTRI-GUT-PD speaking. How have things been going since we last spoke?"*
- Set agenda: *"Today we'll review your goals and the four nutrition topics. This will take around 15minutes, does that work for you?"*

ASSESS Progress (3 min)

- Review goals set at previous contact
- Open-ended questions:
    - *"Tell me about your successes this week."*
    - *"What challenges did you encounter?"*
- Assess adherence to diet plan: *"How closely have you been able to follow the meal plan?"*

ADVISE & REINFORCE (5 min)

Briefly review all four topics - 1 minute each:

1. **Food Processing**: *"How are you doing with choosing minimally processed foods?"*
2. **Healthy Eating**: *"Are you finding it easier to assemble balanced meals?"*
3. **Fiber & Fluids**: *"How is your fiber intake and hydration going?"*
4. **Levodopa-Protein**: *"Are you managing the timing of protein with your medication?"*

- Provide positive reinforcement for successes
- Offer specific advice for challenges identified

AGREE on Next Steps (3 min)

- Collaborative problem-solving for barriers
- Adjust or maintain current goals based on progress
- If goal achieved: *"That's excellent! Would you like to add a new goal or continue with this one to guarantee it is fixed?"*
- If goal not achieved: *"What do you think got in the way? How can we adjust to make this more achievable?"*
- Document any modifications to the diet plan needed

ASSIST with Strategies (1 min)

- Provide specific, actionable strategies for identified challenges
- Example:
    - If struggling with fiber → suggest specific high-fiber options they enjoy
    - If struggling with hydration → suggest morning, afternoon, and evening goals with cups or bottles

ARRANGE Follow-up (1 min)

- Confirm next contact date (next 15 days)
- Encourage continued self-monitoring
- Final encouragement: *"You're making progress. Keep up the good work!"*

<u>CONTROL GROUP</u>

2nd to 6th Sessions *(remote - 10 to 15 minutes total)*

1. Introduction and Engagement

- Greeting: *“Hello [Participant Name], I am calling from the research team to check in on your progress and see how you have been feeling over the last two weeks."*
- Confirm the participant is continuing to complete the daily stool diary.

2. Clinical Monitoring

- Laxative use: *“Have you used any rescue laxatives in the last 15 days? Please confirm if these were recorded in the stool diary?”*
- External advice: *“Since our last contact, have you had a consultation with your neurologist or another healthcare provider?*
    - If yes → *“Did they provide any new recommendations, medications, or changes to your diet?”*
        - *"I have noted those updates. Please continue to follow the guidance provided by your doctor. We will discuss your overall results at the end of the 90 days."*

3. Closing

- Remind the participant of the date for the next call or the final in-person evaluation (D90).
- Thank the participant for their time and contribution to the research.

Part C. Development of the Dietary Intervention Manual

To develop the study's manual for PD, a literature review was conducted. The review assessed guidelines from The European Society for Clinical Nutrition and Metabolism on clinical nutrition in neurology [5], the Brazilian Society of Parenteral and Enteral Nutrition for nutritional therapy in patients with neurodegenerative diseases [6], the Dietetic guideline for Parkinson's disease from the Dutch Parkinson's Disease Association [7], and high-quality reviews and articles addressing dietary aspects of PD [8–11].

Although evidence on nutritional interventions specifically for PD remains limited, available findings generally support dietary patterns emphasizing fresh fruits, vegetables, legumes, nuts, seeds, whole grains, fish, and olive oil, while limiting processed foods, red meat, fried foods, and sweets [8]. These recommendations must be adapted to personal preferences, cultural context, and socioeconomic considerations.

Four core topics were selected for nutritional counseling: healthy eating principles, levels of food processing, importance of fiber and fluid intake, and levodopa–nutrient interactions. A comprehensive manual was developed to support participant understanding and adherence. The manual was initially drafted in Brazilian Portuguese (Part E) by dietitians and subsequently reviewed by experts in nutrition, medicine, speech therapy, and pharmacy. Modifications were implemented based on expert feedback, and the language was tailored to facilitate comprehension by participants. For this publication, the manual text was translated into English to facilitate understanding by international readers, as presented in Part D.

References

1. Barkmeijer A, Te Molder H, Janssen M, Jager-Wittenaar H. Towards effective dietary counseling: a scoping review. Patient Education and Counseling. 2022;105(7):1801–17.

2. Laddu D, Ma J, Kaar J, Ozemek C, Durant RW, Campbell T, et al. Health behavior change programs in primary care and community practices for cardiovascular disease prevention and risk factor management

among midlife and older adults: a scientific statement from the American Heart Association. Circulation. 2021;144(24):e533–49.

3. LeFevre ML, U.S. Preventive Services Task Force. Behavioral counseling to promote a healthful diet and physical activity for cardiovascular disease prevention in adults with cardiovascular risk factors: U.S. Preventive Services Task Force Recommendation Statement. Ann Intern Med. 2014 Oct 21;161(8):587–93. doi:10.7326/M14-1796 PubMed PMID: 25155419.

4. Essat M, Archer R, Williams I, Zarotti N, Coates E, Clowes M, et al. Interventions to promote oral nutritional behaviours in people living with neurodegenerative disorders of the motor system: A systematic review. Clin Nutr. 2020 Aug;39(8):2547–56. doi:10.1016/j.clnu.2019.11.015 PubMed PMID: 31787368.

5. Burgos R, Bretón I, Cereda E, Desport JC, Dziewas R, Genton L, et al. ESPEN guideline clinical nutrition in neurology. Clinical Nutrition. 2018;37(1):354–96.

6. Alves JTM, Gonçalves TJM, Ribeiro PC, de Albuquerque CL, Alvite M de FL, de Assis T, et al. Diretriz BRASPEN de terapia nutricional no paciente com doenças neurodegenerativas. Braspen Journal. 2023;37(2, Supl 2):2–34.

7. van Asseldonk M, Dicke H, van den Beemt B, van den Berg D, ter Borg S, Duin G, et al. DIETETIC GUIDELINE FOR PARKINSON'S. 2012.

8. Subramanian I, Ricciardi L, Schrag A, Appel‐Cresswell S, Kola S, Domingos JM, et al. A Holistic Wellness Prescription for Parkinson's Disease: Evidence‐Based Perspectives and Unmet Needs. Movement Disorders Clinical Practice.

9. Barichella M, Cereda E, Cassani E, Pinelli G, Iorio L, Ferri V, et al. Dietary habits and neurological features of Parkinson's disease patients: implications for practice. Clinical nutrition. 2017;36(4):1054–61.

10. Cereda E, Barichella M, Pedrolli C, Pezzoli G. Low‐protein and protein‐redistribution diets for Parkinson's disease patients with motor fluctuations: a systematic review. Movement Disorders. 2010;25(13):2021–34.

11. Rusch C, Flanagan R, Suh H, Subramanian I. To restrict or not to restrict? Practical considerations for optimizing dietary protein interactions on levodopa absorption in Parkinson's disease. NPJ Parkinsons Dis.

2023 Jun 24;9(1):98. doi:10.1038/s41531-023-00541-w PubMed PMID: 37355689; PubMed Central PMCID: PMC10290638.

Part D. Dietary Intervention Manual – English translation

NUTRI-GUT-PD Study

Nutritional Intervention for Constipation in Parkinson's Disease

Introduction

Food is an important part of health care and makes a difference in daily life. It involves not only nutrients, but also the foods we choose, how we prepare our meals, and the eating habits we build throughout life. Considering these factors, an adequate diet is an important ally for the proper functioning of our body and for managing symptoms, such as constipation. This material was developed within the context of the NUTRI-GUT-PD study by nutritionists affiliated with the Hospital de Clínicas de Porto Alegre and the Federal University of Rio Grande do Sul (UFRGS), based on current recommendations for nutrition and health.

Food Processing

Principle 1

Food Classification According to Type of Processing

When thinking about a healthier diet, it is important to understand how the foods we consume can be classified according to the type of processing used in their production. Knowing these groups helps us reflect on everyday choices and on which foods are most present in our diet.

1) Fresh (In Natura) Foods

Fresh foods are those obtained directly from nature, consisting basically of parts of plants or animals, and that do not undergo industrial processing.

Example: vegetables, legumes, fruits, and meats

2) Minimally Processed Foods

Minimally processed foods are fresh foods that have undergone minimal processing, such as cleaning, removal of non-edible parts, grinding, drying, pasteurization, refrigeration, or freezing.

*Example**:** rice, beans, lentils, dried fruits, nuts without salt or sugar, cassava, corn, tapioca, or wheat flours, fresh pasta, pasteurized or ultra-pasteurized (long-life) milk or milk powder, yogurt (without added sugar), eggs, fresh beef, pork, poultry, and fish, refrigerated or frozen.*

3) Processed Foods

Processed foods are products made from fresh or minimally processed foods, to which salt, sugar, and/or oil are added. These foods undergo simple industrial processes, with the aim of increasing shelf life or modifying flavor.

Example: foods preserved in brine (carrots, cucumbers, peas, olives, corn, and hearts of palm); fruit compotes; salted and smoked meats; canned sardines and tuna; cheeses; and breads.

4) Ultra-Processed Foods

Ultra-processed foods are industrial formulations made mainly from substances extracted from foods or produced in laboratories, containing little or no whole food. These products go through several industrial processes and usually have a long list of ingredients. In general, they contain various additives, such as colorings, flavorings, preservatives, and flavor enhancers, used to improve the product's appearance, taste, and shelf life.

Example: savory crackers, sweet cookies, ice cream, dairy drinks, ready-made sauces (mayonnaise, ketchup, mustard, barbecue sauce), processed meats (ham, sausage, pâté, turkey breast, mortadella), chocolate-flavored drinks, soft drinks, boxed or powdered juice, ready-to-use seasonings in sachets or tablets, instant noodles, packaged snacks, and frozen foods.

Understanding Nutrition Labels

Food labels provide important information about what we are consuming. By looking at the ingredient list, it is possible to identify the quality of the product and the level of processing. Therefore, it is worth paying attention to some important points:

- Ingredients appear in descending order by quantity, meaning the first item on the list is the one present in the largest amount in the food. Whenever possible, avoid products that list sugars or fats as the first ingredients.
- Sugar can appear on the ingredient list under different names, which can make identification more difficult. Some examples include: brown sugar, white sugar, honey, syrup, molasses, glucose, sucrose, maltodextrin, dextrin, dextrose, maltose, fructose, and malt extract.
- Pay attention to the presence of terms such as hydrogenated fat or partially hydrogenated fat, hydrogenated vegetable oil, or partially hydrogenated oil. These terms indicate the presence of trans fat, which is harmful to heart health, even when the label states "zero" or "no trans fat" per serving.
- In general, the shorter and simpler the ingredient list, the better. Give preference to foods whose ingredients you recognize and usually use at home.

Front-of-Pack Nutrition Labeling

Front-of-pack labeling is an information system displayed on the front of food packages and has the main objective of making it easier to understand the nutritional quality of products. Through warning symbols, it indicates when a food has a high content of added sugar, saturated fat, or sodium; nutrients that, when consumed in excess, may be associated with health problems.

Healthy Eating

Principle 2

Ten Steps for Healthy Eating

The Brazilian Dietary Guidelines, developed by the Ministry of Health, present ten practical recommendations to help adopt a healthier diet, respecting the habits and reality of each person.

1) Make fresh or minimally processed foods the basis of your diet

These foods form the foundation of a balanced diet from a nutritional point of view, are tasty, and are aligned with Brazilian food culture, in addition to contributing to a fairer and more sustainable food system. Variety is essential and includes the consumption of different food groups, such as grains, roots, tubers, flours, legumes, vegetables, fruits, nuts, milk, eggs, and meats.

2) Use oils, fats, salt, and sugar in small amounts to season and cook foods and to prepare meals

Oils, fats, salt, and sugar should be used in small quantities because, when consumed in excess, they can be harmful to health. High consumption of salt and saturated fats increases the risk of heart disease, while excess sugar is related to the development of chronic diseases.

- Prefer unsaturated fats**:** Olive oil, Vegetable oils (soybean, canola, corn, sunflower)
- Avoid saturated fats: Butter, Lard, Coconut oil

3) Limit the consumption of processed foods

In small amounts, they can be consumed as ingredients in culinary preparations or as part of meals based on fresh or minimally processed foods.

4) Avoid the consumption of ultra-processed foods

They are nutritionally unbalanced and tend to be consumed in excess, replacing fresh or minimally processed foods. They may be part of the diet as an exception, as long as they are consumed with attention and enjoyment.

5) Eat regularly and mindfully, in appropriate environments, and whenever possible, in company

Try to have meals at similar times every day and avoid eating automatically. Eat slowly, paying attention to signs of hunger and fullness, and try to eat in calm environments,

without distractions such as TV or mobile phones. Whenever possible, have meals in company, making the moment more enjoyable.

6) Shop in places that offer a variety of fresh or minimally processed foods

Markets, street markets, and farmers' markets are good options.

7) Develop, practice, and share cooking skills

Developing and sharing cooking skills encourages home food preparation, without gender distinction. If you do not know how to cook, try to learn with family members, friends, recipes, books, or the internet, and start putting it into practice little by little.

8) Plan how to use your time to give food the space it deserves

Plan weekly grocery shopping and meals, organize the pantry, and share food preparation tasks with the family.

9) Give preference, when eating out, to places that serve freshly prepared meals

In everyday life, look for places that serve meals made to order and at a fair price. Buffet restaurants by weight can be good options, as well as cafeterias that serve homemade food. Avoid fast-food chains.

10) Be critical of information, guidelines, and messages about food conveyed in commercial advertising

Remember: The essential function of advertising is to increase product sales, not to inform or educate people. Critically evaluate what you see, read, and hear about food in commercial advertisements.

Fiber and Fluid Intake

Principle 3

What Are Fibers?

According to official organizations such as the World Health Organization (WHO) and the Codex Alimentarius, dietary fiber is a non-digestible carbohydrate found in foods of

plant origin. In practice, this means that fibers have a composition that our body is not able to digest or "break down."

To help understand this, think of digestive enzymes as scissors. When we eat carbohydrate-rich foods, such as the starch in white bread, these "scissors" cut the food into very small pieces that can enter the bloodstream and be turned into energy. In the case of fiber, our scissors are not able to cut it. For this reason, fiber passes through the stomach and intestines intact.

Types of Dietary Fiber

Soluble Fiber

These are fibers that mix with water and form something thicker, similar to a gel. This "gel" makes the contents of the stomach and intestines heavier and thicker, causing food to take longer to leave the stomach and nutrients to be absorbed more slowly by the body.

Insoluble Fiber

These do not mix with water and remain firm throughout their passage through the intestine. Their main function is to increase stool volume and mechanically stimulate the intestine. This stimulation makes intestinal contents move faster, helping bowel function.

Foods That Are Sources of Dietary Fiber

Fruits: Grapes, bananas, pears, watermelons, persimmons, guava, kiwi, mango, orange, papaya, and pineapple.

Vegetables: Carrot, chayote, eggplant, turnip, spinach, cabbage, cucumber, pumpkin, okra.

Grains and Seeds: Oats, quinoa, chia seeds, sesame seeds, and flaxseed.

"If our body does not digest or absorb fiber, why should we eat it?"

"Even though they are not digested, fiber is essential for intestinal health."

Fluid Intake

Many times we forget, but water is the most important nutrient for our body. Being hydrated is not just about "not feeling thirsty"; it means ensuring that the body has the proper balance to function well, now and in the future. Hydration is a dynamic process of fluid balance, essential for maintaining stable body fluid volume and composition. Water is essential for proper bowel function. It helps form stool and hydrate the fiber in the diet. When water intake is insufficient, stools become dry and hard, which can lead to constipation.

Sources of Hydration

Prefer: Drinking water, Teas and infusions, Natural fruit juices, Coconut water.

Avoid: Boxed juices and powdered juices, Soft drinks, and alcoholic beverages.

Interaction Between Levodopa and Nutrients

Principle 4

Levodopa - Also known as Prolopa®

This is the gold-standard pharmacological treatment for Parkinson's disease and is essential for controlling motor symptoms.

Motor symptoms: Tremors, Rigidity, Bradykinesia (slowness of movement), Akinesia (reduction of voluntary movements).

Medical guidelines usually recommend that the medication be taken on an empty stomach, about 30 minutes before or one hour after meals.

"Why should I take the medication on an empty stomach?"

"To ensure maximum absorption of the medication."

Levodopa Absorption

This recommendation is not random and is directly related to how levodopa is absorbed in the body. After being ingested, levodopa passes through the stomach and is absorbed in the intestine. From there, it needs to reach the brain to exert its effect and help control the symptoms of Parkinson's disease. To make this journey from the intestine to the

brain, levodopa uses specific mechanisms in our body, which are also used by some nutrients present in food, such as proteins, for example.

What Is the Relationship Between Levodopa and Protein?

Levodopa is absorbed in the intestine through the same pathway as food. When we consume protein-rich foods, our body digests them and turn them into amino acids.

What Are Amino Acids?

Amino acids are the final products of protein digestion in the diet. Proteins are essential nutrients for our body and become even more important as we age, as they help maintain muscle mass, strength, balance, and the ability to perform daily activities. When levodopa is taken close to protein-rich meals, part of the medication may not be properly absorbed, reducing its effect on symptom control.

Protein Redistribution

Protein redistribution is a nutritional strategy used in some people with Parkinson's disease to improve the effect of levodopa throughout the day.

- Breakfast and lunch: meals with low protein content are prioritized, favoring the action of the medication during the day.
- Afternoon snack, dinner, and supper: the consumption of protein-rich foods is encouraged, as they are essential for maintaining muscle mass and overall health.

Part E. Dietary Intervention Manual – Original Portuguese version

Pesquisa NUTRI-GUT-PD

Intervenção Nutricional para Constipação em Doença de Parkinson

HOSPITAL DE CLÍNICAS PORTO ALEGRE RS

FONAD

Apresentação

A alimentação é uma parte importante do cuidado com a saúde e pode fazer diferença no dia a dia. Ela não envolve apenas os nutrientes, mas também os alimentos que escolhemos, a forma como preparamos as refeições e os hábitos alimentares que construímos ao longo da vida.

Considerando esses fatores, uma alimentação adequada é uma importante aliada para o bom funcionamento do nosso organismo e manejo dos sintomas, como a constipação.

Este material foi elaborado no contexto do projeto NUTRI-GUT-PD, por nutricionistas vinculadas ao Hospital de Clínicas de Porto Alegre e a Universidade Federal do Rio Grande do Sul (UFRGS), com base em recomendações atuais em alimentação e saúde.

Processamento dos alimentos

Princípio 1

Classificação dos alimentos de acordo com o tipo de processamento

Para pensar em uma alimentação mais saudável, é importante entender como os alimentos que consumimos podem ser classificados conforme o tipo de processamento utilizado na sua produção.

Conhecer esses grupos ajuda a refletir sobre as escolhas do dia a dia e sobre quais alimentos estão mais presentes na sua alimentação.

1) Alimentos *in natura*

Alimentos in natura são aqueles **obtidos diretamente da natureza**, constituídos basicamente por partes de plantas ou de animais, que não passam por processamento industrial.

Exemplo: verduras, legumes, frutas e carnes

2) Alimentos minimamente processados

Alimentos minimamente processados são alimentos in natura que passaram por **processos mínimos**, como **limpeza, remoção de partes não comestíveis, moagem, secagem, pasteurização, refrigeração ou congelamento.**

Exemplo: arroz, feijão, lentilha, frutas secas, castanhas e nozes sem sal ou açúcar; farinhas de mandioca, milho, tapioca ou trigo e massas frescas, leite pasteurizado, ultrapasteurizado ('longa vida') ou em pó, iogurte (sem adição de açúcar), ovos, carnes de gado, de porco e de aves e pescados frescos, resfriados ou congelados.

3) Alimentos processados

Alimentos processados são produtos fabricados a partir de alimentos in natura ou minimamente processados, aos quais são adicionados **sal, açúcar** e/ou **óleo.**

Esses alimentos passam por processos industriais simples, com o objetivo de aumentar a durabilidade ou modificar o sabor.

Exemplo: conservas em salmoura (cenoura, pepino, ervilhas, azeitona, milho e palmito); compotas de frutas; carnes salgadas e defumadas; sardinha e atum de latinha, queijos e pães.

4) Alimentos ultraprocessados

Alimentos ultraprocessados são formulações industriais feitas, principalmente, a partir de substâncias extraídas de alimentos ou produzidas em laboratório, contendo pouco ou nenhum alimento inteiro.

Esses produtos passam por vários processos industriais e costumam apresentar uma lista longa de ingredientes.

Em geral, **contêm diversos aditivos, como corantes, aromatizantes, conservantes e realçadores de sabor**, usados para melhorar a aparência, o sabor e a durabilidade do produto.

Exemplo: bolachas salgadas, bolachas doces, sorvetes, bebida láctea, molhos prontos (maionese, ketchup, mortada, barbecue), embutidos (presunto, salsicha, patê, peito de peru, mortadela), achocolatado, refrigerante, suco de caixinha ou em pó, temperos prontos em sachê ou tablete, macarrão instantâneo, salgadinhos e alimentos congelados.

Prefira sempre alimentos *in natura* ou minimamente processados do que alimentos ultraprocessados

Como identificar cada tipo de alimento?

Através da leitura do rótulo nutricional

Entendendo os rótulos nutricionais

Os rótulos dos alimentos trazem informações importantes sobre o que estamos consumindo. Ao observar a lista de ingredientes, é possível identificar a **qualidade do produto** e o **nível de processamento**. Por isso, vale a pena prestar atenção a alguns pontos importantes:

- Os ingredientes aparecem em ordem decrescente de quantidade, ou seja, o primeiro da lista é o que está presente em maior quantidade no alimento. Sempre que possível, evite produtos que tenham açúcares ou gorduras como os primeiros ingredientes.

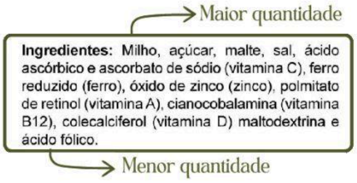

- O açúcar pode aparecer na lista com diferentes nomes, o que pode dificultar sua identificação. Alguns exemplos são: **açúcar mascavo, açúcar cristal, mel, xarope, melado, glicose, sacarose, maltodextrina, dextrina dextrose, maltose, frutose e extrato de malte.**

- Fique atento à presença de termos como **gordura hidrogenada** ou **parcialmente hidrogenada**, **óleo vegetal hidrogenado** ou **parcialmente hidrogenado**. Esses termos indicam a presença de gordura trans, que é prejudicial à saúde do coração, mesmo quando o rótulo informa "zero" ou "sem gordura trans" por porção.

- De modo geral, quanto menor e mais simples for a lista de ingredientes, melhor. Dê preferência aos alimentos cujos ingredientes você reconhece e costuma usar em casa.

Rotulagem nutricional frontal

A rotulagem frontal é um sistema de informação presente na parte da frente das embalagens dos alimentos e tem como principal objetivo facilitar a compreensão sobre a qualidade nutricional dos produtos.

Por meio de símbolos de alerta, ela indica quando um alimento apresenta alto teor de açúcar adicionado, gordura saturada ou sódio, nutrientes que, quando consumidos em excesso, podem estar associados a problemas de saúde.

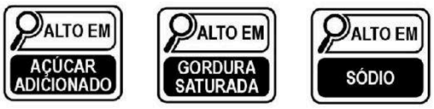

Alimentação saudável

Princípio 2

Dez passos para uma alimentação saudável

O Guia Alimentar para a População Brasileira, elaborado pelo Ministério da Saúde, apresenta dez orientações práticas para ajudar na adoção de uma alimentação mais saudável, respeitando os hábitos e a realidade de cada pessoa.

1) Fazer de alimentos in natura ou minimamente processados a base da alimentação

Esses alimentos formam a base de uma alimentação equilibrada do ponto de vista nutricional, saborosa e alinhada à cultura alimentar, além de contribuírem
para um sistema alimentar mais justo e sustentável.

A variedade é fundamental e envolve o consumo de diferentes grupos de alimentos, como grãos, raízes, tubérculos, farinhas, legumes, verduras, frutas, castanhas, leite, ovos e carnes.

2) Utilizar óleos, gorduras, sal e açúcar em pequenas quantidades ao temperar e cozinhar alimentos e criar preparações culinárias

Óleos, gorduras, sal e açúcar devem ser utilizados em pequenas quantidades porque, quando consumidos em excesso, podem ser prejudiciais à saúde.

O consumo elevado de sal e de gorduras saturas aumenta o risco de doenças do coração, enquanto o excesso de açúcar está relacionado ao desenvolvimento de doenças crônicas.

Prefira gorduras insaturadas

Azeite de oliva
Óleos vegetais
(soja, canola, milho, girassol)

Evite gorduras saturadas

Manteiga
Banha de porco
Óleo de coco

3) Limitar o consumo de alimentos processados

Em pequenas quantidades, podem ser consumidos como ingredientes de preparações culinárias ou parte de refeições baseadas em alimentos in natura ou minimamente processados.

4) Evitar o consumo de alimentos ultraprocessados

São nutricionalmente desbalanceados e tendem a ser consumidos em excesso e a substituir alimentos in natura ou minimamente processados.

Podem fazer parte da alimentação, como exceção, desde que consumidos com atenção e prazer.

5) Comer com regularidade e atenção, em ambientes apropriados e, sempre que possível, com companhia.

Procure fazer as refeições em horários semelhantes todos os dias e evite comer no automático.

Coma devagar, prestando atenção aos sinais de fome e saciedade, e procure se alimentar em ambientes tranquilos, sem distrações como TV ou celular.

Sempre que possível, faça as refeições em companhia, tornando esse momento mais agradável.

6) Fazer compras em locais que ofertem variedades de alimentos in natura ou minimamente processados

Mercados, feiras livres e feiras de produtores são boas sugestões.

7) Desenvolver, exercitar e partilhar habilidades culinárias

Desenvolva e compartilhe habilidades culinárias, incentivando o preparo de alimentos em casa, sem distinção de gênero. Caso não saiba cozinhar, busque aprender com familiares, amigos, receitas, livros, internet e comece aos poucos a colocar isso em prática.

8) Planejar o uso do tempo para dar à alimentação o espaço que ela merece

Planeje as compras e as refeições da semana, organize a despensa e compartilhe com a família as tarefas relacionadas ao preparo dos alimentos.

9) Dar preferência, quando fora de casa, a locais que servem refeições feitas na hora

No dia a dia, procure locais que servem refeições feitas na hora e a preço justo. Restaurantes de comida a quilo podem ser boas opções, assim como refeitórios que servem comida caseira. Evite redes de fast food.

10) Ser crítico quanto a informações, orientações e mensagens sobre alimentação veiculadas em propagandas comerciais

Lembre-se:

a função essencial da publicidade é aumentar a venda de produtos e não informar ou, menos ainda educar as pessoas. **Avalie com crítica** o que você lê, vê e ouve sobre alimentação em propagandas comerciais.

Como montar um prato saudável?

Incluindo hortaliças, alimentos fonte de carboidrato e proteína

Ingestão de fibra e líquidos

Princípio 3

O que são as fibras ?

De acordo com órgãos oficiais como a Organização Mundial da Saúde (OMS) e o Codex Alimentarius, a fibra alimentar é um **carboidrato não digestível** presente em alimentos de **origem vegetal**.

Na prática, isso significa que as fibras têm uma composição que o nosso corpo não consegue digerir ou "quebrar".

Para entender pense nas **enzimas digestivas** como **tesouras.**

Quando comemos alimentos fonte de carboidrato, como o amido do pão branco, essas "tesouras" cortam o alimento em pedaços bem pequenos, que conseguem entrar na corrente sanguínea e virar energia.

No caso das fibras, nossas tesouras não conseguem fazer o corte. Por isso, a fibra passa intacta pelo estômago e pelo intestino

Tipos de fibras alimentares

Fibras solúveis

São fibras que se misturam com a água e formam algo mais grosso, parecido com um gel.

Esse "gel" deixa o conteúdo do estômago e do intestino **mais pesado e espesso**, fazendo com que a **comida demore mais para sair do estômago** e que os **nutrientes sejam absorvidos mais lentamente** pelo organismo.

X

Fibras insolúveis

Essas não se misturam com a água e permanecem firmes durante todo o percurso pelo intestino.

A principal função é **aumentar o volume das fezes e estimular mecanicamente o intestino**. Esse estímulo faz com que o conteúdo intestinal avance **mais rápido**, facilitando o funcionamento do intestino.

Alimentos fontes de fibras alimentares

Frutas

Banana, Uva, Caqui, Goiaba, Laranja, Mamão, Pera, Melancia, Kiwi, Manga, Abacaxi

Hortaliças

Cenoura, Chuchu, Beringela, Nabo, Espinafre, Repolho, Pepino, Abóbora, Quiabo

Cereais

Aveia, Quinoa, Chia, Gergelim, Linhaça

Se nosso corpo não digere nem absorve as fibras, por quê devemos come-las?

Mesmo não sendo digeridas, as fibras são fundamentais para a saúde intestinal

Consumo hídrico

Muitas vezes esquecemos-nos, mas a água é o nutriente mais importante do nosso organismo. Estar hidratado **não é apenas "não ter sede"**; é garantir que o corpo tenha o equilíbrio perfeito para funcionar bem, hoje e no futuro.

A hidratação é um processo dinâmico de **balanço hídrico**, essencial para manter o volume e a composição dos fluidos corporais estáveis.

A A água é essencial para o bom funcionamento do intestino. Ela ajuda a formar as fezes e a hidratar as fibras da alimentação.

Quando a ingestão de água é insuficiente, as fezes ficam ressecadas e duras, o que pode levar à constipação intestinal

Fontes de hidratação

Prefira	Evite
Água potável	Suco em caixa e suco em pó
Sucos naturais	Bebidas alcoólicas
Chás e infusões	Refrigerante
Água de coco	

Interação entre Levodopa e nutrientes

Princípio 4

Levodopa

Também conhecida como Prolopa®

Este é o **tratamento farmacológico padrão-ouro** para a Doença de Parkinson, sendo fundamental para o **controle dos sintomas motores.**

Sintomas motores

- Tremores;
- Rigidez;
- Bradicinesia (lentidão dos movimentos);
- Acinesia (diminuição de movimentos voluntários).

As orientações médicas costumam indicar que o medicamento seja **tomado em jejum**, cerca de **30 minutos antes ou 1 hora após as refeições**.

Por que eu devo tomar o remédio em jejum?

Para garantir a máxima absorção do medicamento.

Absorção da Levodopa

Essa recomendação não é aleatória e está diretamente relacionada ao modo como a levodopa é absorvida no organismo.

Depois de ingerida, a levodopa passa pelo estômago e é **absorvida no intestino**.

A partir daí, ela precisa chegar ao **cérebro** para exercer seu efeito e ajudar no **controle dos sintomas da Doença de Parkinson.**

Para conseguir fazer esse caminho do intestino até o cérebro, a levodopa utiliza mecanismos específicos do nosso organismo, que também são usados por alguns nutrientes presentes na alimentação, **como por exemplo as proteínas.**

Qual a relação entre a levodopa e a proteína?

A levodopa é absorvida no instestino pelo mesmo caminho que os alimentos. Quando consumimos **alimentos ricos em proteína**, o nosso corpo os digere e transforma em **aminoácidos**.

O que são aminoácidos?

Os aminoácidos são os produtos finais da digestão das proteínas que consumimos na alimentação.

As proteínas são nutrientes essenciais para o nosso corpo e se tornam ainda mais importantes com o passar dos anos, pois ela ajudam a **manter a massa muscular**, a **força**, o **equilíbrio** e a **capacidade de realizar atividades do dia a dia.**

Quando a levodopa é tomada próxima a refeições ricas em proteínas, parte do medicamento pode não ser absorvida adequadamente, **diminuindo seu efeito no controle dos sintomas.**

Redistribuição de proteínas

A redistribuição de proteínas é uma estratégia nutricional utilizada em algumas pessoas com Doença de Parkinson para melhorar o efeito da levodopa ao longo do dia.

- **Café da manhã e almoço:** priorizam-se refeições com baixo teor de proteínas, favorecendo a ação do medicamento durante o dia.
- **Lanche da tarde, jantar e ceia:** o consumo de alimentos ricos em proteínas é incentivado, pois eles são essenciais para a manutenção da musculatura e da saúde geral.

Sugestão de cardápio

Baixo teor de proteína

Café da manhã

- Frutas
- Café preto
- Pão
- Geleia

Lanche da manhã

- Fruta
- Aveia
- Oleogenosas (castanhas, nozes)

Almoço

- Arroz
- Feijão
- Salada variada

Sugestão de cardápio

Alto teor de proteína

Lanche da tarde

- Crepioca (ovo + tapioca)
- Frango
- Creme de ricota
- Tomate e cenoura

Jantar

- Massa
- Molho à bolonhesa
- Salada variada

Ceia

- Iogurte natural
- Frutas
- Aveia
- Chia

Receitas saudáveis

Baixo teor de proteína

Salada tropical

Ingredientes

- Folhas verdes de seu interesse (alface, rúcula, agrião, acelga, folhas de beterraba)
- ½ pepino fatiado
- ½ tomate picado
- ½ xícara de manga picada
- ½ cebola roxa picada

Ingredientes do molho

- 100 g de iogurte natural desnatado
- 1 colher de sopa de suco de limão
- 1 colher de sopa de azeite de oliva
- Sal a gosto
- Pimenta do reino a gosto

Preparação

1. Deixe a cebola picada de molho em água fria por 20 minutos.
2. Coe cuidadosamente a cebola.
3. Acrescente as folhas, o pepino, o tomate, a manga e a cebola roxa em uma vasilha.
4. Misture os ingredientes do molho em um uma cumbuca ou pote.
5. Acrescente o molho à salada.

Iogurte com frutas e aveia

Ingredientes

- 1 pote (160g) de iogurte natural
- Frutas de sua preferência (ex.: maçã, morango, banana)
- 2 colheres de sopa de flocos de aveia

Preparação

1. Higienize as frutas de sua escolha
2. Pique as frutas.
3. Em uma vasilha, adicione o iogurte.
4. Adicione as frutas e a aveia.

Pudim de chia

Ingredientes

- 2 colheres de sopa cheias de semente de chia
- 150ml de leite
- 1 banana amassada ou picada
- 1 colher de café de canela

Preparação

1. Em um pote com tampa, adicione a semente de chia e a canela.
2. Acrescente 150mL de leite.
3. Misture os ingredientes.
4. Adicione a banana picada.
5. Leve a geladeira por pelo menos 6 horas.

Referências bibliográficas

Brasil. Ministério da Saúde. Secretaria de Atenção à Saúde. Departamento de Atenção Básica. Guia alimentar para a população brasileira. 2 ed. Brasília: Ministério da Saúde, 2014.

Gill, Samantha K., et al. "Dietary fibre in gastrointestinal health and disease." Nature Reviews Gastroenterology & Hepatology 18.2 (2021): 101-116.

Mathers, John C. "Dietary fibre and health: the story so far." Proceedings of the Nutrition Society 82.2 (2023): 120-129.

Erhardt, Rene, et al. "Functional constipation and the effect of prebiotics on the gut microbiota: a review." British Journal of Nutrition 130.6 (2023): 1015-1023.

Beke, Matthew P., and Wendy J. Dahl. "Managing Constipation and Its Risks in Parkinson's Disease: Is There a Role for Dietary Fiber?." Canadian Journal of Dietetic Practice and Research (2025): 1-7.

Alves, Juliana Tepedino Martins, et al. "Diretriz BRASPEN de terapia nutricional no paciente com doenças neurodegenerativas." Braspen Journal 37.2, Supl 2 (2023): 2-34.

MARTINEZ, Anna Paula; AZEVEDO, Gisele Regina de. Tradução, adaptação cultural e validação da Bristol Stool Form Scale para a população brasileira. Revista Latino-Americana de Enfermagem, Ribeirão Preto, v. 20, n. 3, p. [7 telas], maio-jun. 2012.

Bloem, Bastiaan R., Michael S. Okun, and Christine Klein. "Parkinson's disease." The Lancet 397.10291 (2021): 2284-2303.

Keun, Jikke T. Boelens, et al. "Dietary approaches to improve efficacy and control side effects of levodopa therapy in Parkinson's disease: a systematic review." Advances in Nutrition 12.6 (2021): 2265-2287.

Combinados feitos com a nutricionista
