# Supplementary material for: Dietitian-led intervention to manage constipation in Parkinson’s disease: Study protocol for a parallel-group randomized controlled trial (NUTRI-GUT-PD)
Source: PLoS One. 2026 Jul 27;21(7):e0354608. doi: 10.1371/journal.pone.0354608 (PMC13405298; doi:10.1371/journal.pone.0354608)
Supplement: S3 Appendix — (PDF) [file pone.0354608.s003.pdf]

FEDERAL UNIVERSITY OF RIO GRANDE DO SUL
POSTGRADUATE PROGRAM IN MEDICINE: MEDICAL SCIENCES

NUTRITIONAL INTERVENTION FOR CONSTIPATION SYMPTOMS IN PATIENTS WITH PARKINSON'S DISEASE: A RANDOMIZED CLINICAL TRIAL

The NUTRI-GUT-PD study

Juliana Heitich Brendler
Juan Sebastian Sanchez Leon
João Vitor Barboza Cardoso
Khadija Younes da Silva
Andreza Francisco Martins
Valesca Dall'Alba
Artur Francisco Schumacher Schuh
Maira Rozenfeld Olchik

2025

RESEARCH TEAM

NAME	EDUCATION	AFFILIATION	RESPONSIBILITIES
Juliana Heitich Brendler	Nutritionist, MSc	PhD student in the Graduate Program in Medicine: Medical Sciences (PPGCM)	- Analyze collected data<br>- Apply data collection instruments<br>- Conduct the informed consent process<br>- Organize study materials<br>- Conduct research interviews and study visits<br>- Register and update the project<br>- Communicate with the Research Ethics Committee (CEP)
Juan Sebastian Sanchez Leon	Neurologist	Movement Disorders Fellow at Hospital de Clínicas de Porto Alegre (HCPA)	- Apply data collection instruments<br>- Conduct research interviews and study visits
João Vitor Barboza Cardoso	Pharmacist	Master's student in PPGCM	- Analyze collected data<br>- Organize study materials<br>- Conduct research assessments
Khadija Younes da Silva	Undergraduate Nutrition Student	Undergraduate Research Student at the Federal University of Rio Grande do Sul (UFRGS)	- Apply data collection instruments<br>- Organize study materials<br>- Conduct research interviews and study visits
Andreza Francisco Martins	Pharmacist, PhD	Professor in the Department of Microbiology, Immunology and Parasitology at UFRGS; faculty member of the Graduate Program in Pharmaceutical Sciences (UFRGS) and the PPGCM	- Analyze collected data<br>Conduct research assessments
Valesca Dall'Alba	Nutritionist, PhD	Professor in the Nutrition undergraduate program at UFRGS and faculty member of the Graduate Programs in Gastroenterology and Hepatology and in Food, Nutrition and Health	- Analyze collected data
Artur Francisco Schumacher Schuh	Neurologist, PhD	Head of the Neurology Service at HCPA and Professor in the Department of Pharmacology and the PPGCM	- Analyze collected data<br>- Sign documents for submission to CEP<br>- Manage the study's financial resources
Maira Rozenfeld Olchik	Speech-Language Pathologist, PhD<br>**Principal Investigator*	Professor in the Speech-Language Pathology undergraduate program at UFRGS and faculty member of the PPGCM	- Analyze collected data<br>- Sign documents for submission to CEP<br>- Manage the study<br>- Manage the study's financial resources<br>- Communicate with CEP

ABSTRACT

Intestinal constipation is one of the most prevalent non-motor symptoms of Parkinson's disease (PD), negatively impacting quality of life and treatment adherence. Evidence suggests that healthy dietary patterns may contribute to relieving these symptoms and modulating gut microbiota. However, there is a lack of clinical trials investigating the impact of nutritional interventions in patients with PD. In this context, the NUTRI-GUT-PD research project aims to evaluate the effect of a nutritional intervention on constipation symptoms in individuals with PD. This is a randomized controlled clinical trial including participants diagnosed with PD and functional constipation. Participants will be randomly assigned to one of two groups: (1) a nutritional intervention group, consisting of an individualized meal plan and follow-up with a nutritionist for three months, and (2) a control group, which will receive nutritional guidance only at the end of the study period. The nutritional intervention will be based on the Dietary Reference Intakes (DRIs), in combination with recommendations from the literature for the management of PD-specific nutritional needs and constipation symptoms. Assessments will be conducted at baseline, midway, and at the end of the intervention period. The following outcomes will be evaluated: weekly bowel movement frequency, stool consistency (Bristol Stool Scale), constipation symptoms using the Constipation Scoring System, gut microbiota composition, dietary intake, diet quality, nutritional status, and clinical parameters related to PD. At the end of the study, it is expected that the nutritional intervention will lead to improvement in constipation symptoms, greater gut microbial diversity, improved diet quality, and better nutritional status among participants.

Keywords: Parkinson’s disease; Constipation; Gut microbiota; Nutritional counseling; Healthy diet.

LIST OF ABBREVIATIONS AND ACRONYMS

BIA – Bioelectrical Impedance Analysis

AC – Abdominal Circumference

AC – Arm Circumference

CEP – Research Ethics Committee

CC – Calf Circumference

CPC – Clinical Research Center

CSS – Constipation Scoring System

PD – Parkinson's Disease

HS – Handgrip Strength

HCPA – Porto Alegre Clinical Hospital

BMI – Body Mass Index

MAN-VR – Mini Nutritional Assessment – Reduced Version

MDS-UPDRS – Movement Disorder Society – Unified Parkinson’s Disease Rating Scale

MoCA – Montreal Cognitive Assessment

NOVA-UPF – Nova Ultra-processed Foods

NOVA-WPF – Nova Whole Plant Foods

PPGCM - Graduate Program in Medicine: Medical Sciences

24hFR – 24-hour Food Recall

SARC-F – Sarcopenia Screening Tool

SDQ – Swallowing Disturbance Questionnaire

TACO – Brazilian Table of Food Composition

ICF– Free and Informed Consent Form

GI– Gastrointestinal Tract

TUG – Timed Up and Go

SUMMARY

1. INTRODUCTION...7
2. JUSTIFICATION...9
3. OBJECTIVES...10
3.1 Primary Objective...10
3.2 Secondary Objectives...10
4. METHODS...10
4.1 Study Design...10
4.2 Participants and recruitment...11
4.2.1 Inclusion criteria...11
4.2.2 Exclusion criteria...11
4.2.3 Risks and benefits...12
4.3 Randomization and blinding...12
4.4 Primary Outcome...13
4.5 Sample size...13
4.6 Procedures by groups...13
4.6.1 Control group...13
4.6.2 Intervention group...14
4.7 Assessments...15
4.7.1 Constipation...15
4.7.2 Gut Microbiome...16
4.7.3 Diet...17
4.7.4 Nutritional Status...18
4.7.5 Sociodemographic data...20
4.7.6 Clinical Data...20
4.8 Statistical analysis...21
4.9 Ethical aspects...22
5. BUDGET...23
6.TIMELINE...24
REFERENCES...25
APPENDIX A - SPIRIT 2025 checklist...29
APPENDIX B - Informed Consent Form (ICF)...31
APPENDIX C - Sociodemographic Questionnaire...35

1. INTRODUCTION

Parkinson's Disease (PD) is a progressive neurodegenerative disease that affects the central nervous system (CNS) and is currently the most prevalent movement disorder in the world (Balestrino; Schapira, 2020; Dorsey et al., 2018; Pringsheim et al., 2014). The prevalence of the disease has been increasing globally in recent decades, and between 1990 and 2015 it rose to 6.2 million people with PD (Dorsey et al., 2018a, 2018b). In Latin America and the Caribbean, the prevalence of PD is 1,081 per 100,000 inhabitants (Pereira et al., 2024).

The disease is characterized by motor symptoms such as: tremor, bradykinesia, rigidity, and postural instability, which result from the degeneration of dopaminergic neurons in the substantia nigra of the brain and the depletion of striatal dopamine (Tolosa et al., 2021). In addition to this, PD presents with non-motor symptoms (NMS) that are particularly complex as they involve multiple brain regions and the participation of other neurotransmitters (Munhoz et al., 2015). These manifestations include hyposmia, constipation, urinary dysfunction, orthostatic hypotension, memory loss, depression, pain, and sleep disturbances (Pont‐Sunyer et al., 2015; Tolosa et al., 2021). Constipation emerges as one of the most reported symptoms by people with PD, being mentioned in 20% to 89% of cases, which represents an incidence 2 to 4 times higher than in the general population (Pfeiffer, 2011).

The term "constipation" is often used generically to define any difficulty with evacuation. However, functional constipation is defined by the Rome IV criteria as persistent symptoms of unsatisfactory evacuation in the absence of identifiable organic causes (Drossman; Hasler, 2016). In PD, constipation results from pathophysiological impairment of colonic motility that affects the entire gastrointestinal tract (GIT). The literature has suggested that the gut may be one of the initial sites of PD pathophysiology, as proposed by the Braak hypothesis, which suggests the retrograde transmission of pathological alpha-synuclein from the gastrointestinal tract to the CNS via the vagus nerve (Yemula et al., 2021). Individuals with chronic constipation have a 3 to 11 times higher risk of developing PD, and data from prospective and retrospective studies suggest that constipation becomes evident on average between 15.6 and 24.0 years before diagnosis (Postuma et al., 2013; Yu et al., 2018).

Constipation may also be related to changes in the composition of the gut microbiota, which has been increasingly recognized as part of the pathophysiological process of PD. Evidence suggests that patients with PD have a reduction in microbial diversity and an imbalance between beneficial and potentially pathogenic bacteria, known as dysbiosis (Salim et al., 2023). This imbalance can contribute to increased intestinal permeability, activation of the immune system, and chronic inflammation, factors that are associated with the progression of neurodegeneration. The gut microbiota also communicates with the central nervous system through the microbiota-gut-brain axis, influencing gastrointestinal motility, neurotransmitter synthesis, and the function of epithelial and blood-brain barriers (Uyar; Yildiran, 2019).

Some dietary factors, such as excessive consumption of ultra-processed foods, which are rich in additives and low in fiber, can further compromise gut health, aggravating dysbiosis and contributing to constipation (Song et al., 2023). A cross-sectional study identified that the microbiota pattern in PD patients was related to eating habits and that a healthier diet can increase the abundance of bacteria with potential anti-inflammatory effects, while reducing the presence of bacteria with a pro-inflammatory profile in PD patients. Suggesting that consuming a good quality diet can contribute to controlling pro-inflammatory dysbiosis in these patients and possibly offer some protection against disease progression (Kwon et al., 2024).

A study conducted by Barichella et al. (2017) analyzed the eating habits and neurological characteristics of 600 people with PD compared to 600 healthy controls. The results showed that individuals in the PD group had a lower BMI, higher caloric and protein intake, and a higher prevalence of constipation. Constipation was reported by 46.8% of PD patients, which was significantly higher than the controls (6.8%). Despite a higher fiber intake than controls, water intake was considerably lower, which may compromise the effects of fiber on intestinal function. Furthermore, constipation was associated with higher doses of levodopa, suggesting that changes in intestinal transit and microbiota may reduce the bioavailability of the drug. The study reinforces the importance of including systematic nutritional assessment in the multidisciplinary management of the disease, with a special focus on weight control, constipation management, and adequacy of protein intake to optimize therapeutic efficacy (Barichella et al., 2017).

Several strategies have been proposed in randomized clinical trials (RCTs) to reduce constipation symptoms and improve microbiota diversity in PD patients. Most focus on the use of probiotics or specific drugs with a laxative effect. Probiotics in general demonstrate quite positive effects, with increased bowel frequency, improved stool consistency, decreased intestinal transit time, reduced use of laxatives, improved quality of life, and also improved microbiota (Du et al., 2022; Ibrahim et al., 2020; Tan et al., 2021; Yang et al., 2023). The intervention time in these studies ranged from 8 to 12 weeks with daily consumption, sometimes several times a day, of the proposed probiotic. Medications with a laxative effect also showed safety and positive results in improving constipation symptoms (Hatano et al., 2024; Kubo et al., 2024; Ondo et al., 2012; Parkinson Study Group, 2017; Zangaglia et al., 2007). However, the long-term effect of continuous use of these probiotics and medications is unknown. In contrast, these strategies may not be feasible in diverse socioeconomic contexts.

We identified only one RCT that performed dietary intervention, implementing the Mediterranean diet (Rusch et al., 2024). This study compared a group that received standard treatment (recommendations on physical activity, fiber and liquid consumption, and use of laxatives) with a group that received standard treatment associated with the Mediterranean diet. Both interventions

reduced constipation symptoms, although the change was not significantly different between the groups.

Given this scenario, diet has gained prominence as a promising non-pharmacological intervention in the management of PD. Studies suggest that healthy eating patterns may be associated with improved motor and non-motor symptoms, in addition to contributing to the maintenance of patients' functionality and autonomy (Rees et al., 2024). However, it is observed that the diet quality of individuals with PD is often inadequate, characterized by high consumption of ultra-processed foods and low intake of fiber and essential micronutrients (Dunk et al., 2023; Kwon et al., 2023).

Nutritional monitoring emerges not only as support for pharmacological treatment but as a potential therapeutic intervention to improve intestinal function and the quality of the microbiota of these individuals. Despite advances, randomized clinical studies that structurally evaluate the impact of nutritional interventions for people with PD are still scarce. Thus, investigation through RCTs of the effect of this monitoring on constipation symptoms and microbiota diversity is necessary.

2. JUSTIFICATION

Constipation is one of the most prevalent NMS in PD, impacting the gut microbiota and the effectiveness of drug therapy, in addition to being associated with clinical complications such as abdominal distension and increased risk of intestinal perforation (Cirstea et al., 2020; Yemula et al., 2021). Although there are RCTs on pharmacological approaches and the use of probiotics with positive results, these strategies may present limitations regarding economic feasibility, long-term adherence, and safety, especially in populations with less access to health resources (Hatano et al., 2024; Tan et al., 2021).

Given this scenario, interventions based on healthy eating gain relevance because they are low-cost, culturally adaptable, and have potential benefits in various other aspects. Growing evidence suggests that dietary patterns rich in fiber, micronutrients, and whole foods can contribute not only to improving constipation symptoms but also to the positive modulation of the gut microbiota, a factor that has been studied in the pathophysiology of PD (Kwon et al., 2023; Pourmotabbed et al., 2025; Song et al., 2023).

Despite this, there is still a gap in the literature regarding the contribution of nutritionists to the care of patients with PD. The literature already highlights several nutritional particularities of this population, such as the interaction between dietary protein and levodopa, frequent weight loss and the risk of malnutrition, in addition to dysphagia and constipation. However, more studies are still needed to substantiate more robust guidelines on the recommended nutrient quantities for these patients.

Thus, the research question arises: In individuals with PD and functional constipation, is nutritional intervention with a nutritionist's follow-up effective in reducing constipation symptoms and modulating the gut microbiota? This study intends to contribute to the production of nutritional guidelines in the care of people with PD, offering a safe, accessible, and sustainable therapeutic alternative for the management of constipation.

3. OBJECTIVES

3.1 Primary Objective

To evaluate the efficacy of a nutritional intervention with dietitian follow-up on the number of bowel movements per week in individuals with Parkinson's Disease and functional constipation.

3.2 Secondary Objectives

1. To evaluate the effect of the nutritional intervention compared to the control on the intestinal microbiota composition of people with PD;
2. To relate the clinical aspects of PD with the intestinal microbiota composition;
3. To evaluate the food intake and diet quality of people with PD, as well as the effect of the nutritional intervention on these parameters;
4. To evaluate the nutritional status of patients with PD and the effects of the nutritional intervention on this outcome;
5. To identify the clinical characteristics associated with the effect of the nutritional intervention in patients with PD.

4. METHODS

4.1 Design

This is a 3-month (90 days) parallel randomized clinical trial to be conducted at Hospital de Clínicas de Porto Alegre (HCPA) (SPIRIT 2025 checklist - APPENDIX A)

4.2 Participants and recruitment

Recruitment of participants for the NUTRI-GUT-PD study will be carried out from the LARGE-PD study database (2024-0445). LARGE-PD participants linked to HCPA are invited during in-person appointments at the Parkinson's Disease and Movement Disorders Research Group. Participants outside the institution can volunteer by filling out a questionnaire available in the promotional material. The outpatient clinic has a broad and consolidated base of regularly followed-up patients, which facilitates access to the necessary number of eligible participants.

Participants of LARGE-PD evaluated in the last three months who have the marking “Patient agrees with LGPD Consent Form” and who meet the inclusion and exclusion criteria will be considered for NUTRI-GUT-PD. These participants will be contacted by phone for a formal invitation and, if they accept, a room will be scheduled at the CPC for the detailed explanation and in-person signing of the Informed Consent Form (ICF – APPENDIX B). It will also be possible to invite LARGE-PD participants immediately after their evaluation at the CPC, checking their interest in joining NUTRI-GUT-PD and, if positive, the ICF will be applied in person. All those who accept will be included after signing the ICF.

4.2.1 Inclusion Criteria

Adults with a prior diagnosis of Parkinson's Disease, confirmed by a neurologist based on the Movement Disorders Society criteria (Postuma et al., 2015), and who have been on a stable dose of levodopa for at least 3 months will be included. Furthermore, participants must report at least two of the criteria defined by the Rome IV protocol for the characterization of Functional Constipation (Drossman; Hasler, 2016):

- Straining during more than 25% of defecations;
- Lumpy or hard stools (Bristol Stool Form Scale types 1–2) in more than 25% of defecations;
- Sensation of incomplete evacuation for more than 25% of defecations;
- Sensation of anorectal obstruction or blockage for more than 25% of defecations;
- Need for manual maneuvers to facilitate defecation (e.g., digital evacuation, support of the pelvic floor) in more than 25% of defecations;
- Fewer than three spontaneous bowel movements (SBM) per week;
- Loose stools are rarely present without the use of laxatives;
- Insufficient criteria for irritable bowel syndrome;

4.2.2 Exclusion criteria

Individuals who meet one or more of the following conditions will be excluded:

1. Diagnosis of atypical or secondary parkinsonism;
2. Hoehn and Yahr stage greater than 2;
3. Presence of severe neurological or psychiatric diseases that compromise the ability to participate in study procedures;
4. Diagnosis of previous dementia;
5. Presence of comorbidities such as active neoplasms, chronic obstructive pulmonary disease, heart failure and/or chronic kidney failure.
6. History of gastrointestinal neoplasms, inflammatory bowel diseases or surgeries involving the gastrointestinal tract;
7. Constipation secondary to clinical conditions, such as hypothyroidism or diabetes mellitus;

8. Opioid use;
9. Use of probiotics or antibiotics in the last 3 months;
10. Continuous use of laxatives in the last 8 weeks;
11. Presence of severe dysphagia according to the Functional Oral Intake Scale (FOIS) classification;
12. Individuals using enteral or parenteral nutrition;
13. Individuals receiving nutritional follow-up in the last 3 months.

4.2.3 Risks and Benefits

- Risks: Participation in the research does not involve known significant risks. However, discomfort may arise from the time taken to respond to the questionnaires and evaluations, considering that each visit to the CPC will last approximately 1 hour and 30 minutes. Furthermore, some instruments include questions of a personal nature, which may generate embarrassment or discomfort. Anthropometric measurements and the walking test, although non-invasive, may eventually cause some physical discomfort. It is important to emphasize that all adopted procedures are non-invasive.
- Benefits: Potential benefits for the participant include access to detailed information about their nutritional status, through assessments of food intake and body composition (muscle mass, fat, and water). Participants will also receive individualized nutritional guidance performed by a nutritionist, provided at the beginning or end of the study, contributing to a greater understanding and management of their eating habits. From a collective point of view, participation will help expand scientific knowledge about the relationship between diet and health in people with PD, enabling the development of more effective care and nutritional guidance strategies in future contexts.

4.3 Randomization and blinding

Randomization will be performed by an independent researcher, not involved in data collection or clinical evaluations. The method used will be simple, computer-generated randomization, in a 1:1 ratio, using a random number table. The parallel groups are: (1) intervention group, which will receive a food plan, dietary guidance and follow-up with a nutritionist, and (2) control group.

The researcher responsible for the final evaluations will be blinded to the participants' allocation. Similarly, the professionals responsible for microbiota sequencing and analysis, as well as the statistician in charge of data analysis, will also remain blinded. Participants' randomization will occur after the initial evaluation, so blinding of the researchers responsible for this initial step will not be necessary. However, the researchers involved in the nutritional intervention will not be blinded,

since this measure is not necessary; furthermore, they will be responsible for the evaluations carried out on days 45 and 90 of the study.

4.4 Primary Outcome

The primary outcome of the study is the frequency of bowel movements per week, which will be assessed through the bowel movement diary.

4.5 Sample Size

A sample size of 48 subjects (24 for each group) was calculated to test whether there is a minimum difference of 1 bowel movement/week between the mean bowel movements per week of the intervention and control groups at the last time point (week 12) of the repeated measures study (with the addition of 10% for possible losses and refusals, this number should be 54, 27 for each group). The calculation considered 80% power, a significance level of 5%, a standard deviation of 1.15 and 1.21 bowel movements/week for the intervention and control groups, respectively (Rusch et al., 2024). As well as 90% retention at time points 2 and a permutable correlation matrix with a correlation of 0.5. This calculation was performed using the PSS Health online tool (Borges et al., 2020).

4.6 Procedures by groups

The individuals included in the study will undergo the complete initial assessment (baseline) and will then be randomized into control or intervention groups. After randomization, participants will return to the CPC within 7 days (D0) for stool sample delivery, and participants in the intervention group will receive nutritional guidance.

At 45 days (D45), dietary intake and constipation will be assessed via telephone. At the end of the study (D90), participants will be called to return to the CPC for the complete final assessment. At this time, if positive, participants in the control group will receive complete nutritional guidance. The researchers responsible for the final assessment and data processing will be exclusive to these tasks and will not have access to randomization and group allocation information.

4.6.1 Control group

After the study period (D90), if the intervention shows benefits, the participants in the control group will receive printed material and guidance on healthy eating, fiber and fluid consumption, as well as information about the relationship between protein and dopamine. The intervention will be carried out in a waitlist format, ensuring that all participants benefit, even if at different times. This strategy aligns with the study's objective of evaluating the effect of a nutritional intervention with nutritionist monitoring, while also preserving the ethical principle of beneficence for everyone involved.

4.6.2 Intervention Group

Participants allocated to the nutritional intervention group will receive complete nutritional follow-up, including a calculated meal plan, nutritional guidance, and support via telephone calls throughout the study.

First Nutritional Consultation (D0)

Based on the baseline assessments, a meal plan will be developed for each participant according to the proposed protocol below. In this first consultation, participants will receive guidance on: healthy eating and the level of food processing based on the Food Guide for the Brazilian Population (2014); sources of dietary fiber and the importance of adequate fluid intake; levodopa-nutrient interaction and the importance of the fasting period before and after taking the medication.

Diet plan calculation protocol

The meal plan will be calculated by a nutritionist on the DietBox © platform, using the Brazilian Food Composition Table (TACO) - 4th edition as the basis for food selection. The daily energy will be calculated based on the 2023 equation for Estimated Energy Requirement (EER) (Cominetti; Cozzolino, 2023).

For the calculation of daily protein, the Recommended Dietary Allowance (RDA) for healthy adults of 0.8 g PTN/kg/day will be used. The Protein Redistribution Diet (PRD) will also be implemented, which consists of restricting the consumption of protein-rich foods at breakfast and lunch, concentrating protein intake at dinner. The study by Barichella et al. (2017) demonstrated that PRD is associated with improved response to levodopa, with a reduction in motor fluctuations and the total required dose of the drug. It was also identified that daily protein consumption above the recommendation (0.8g/kg/day) was associated with higher doses of levodopa (Barichella et al., 2017). Recommendations for daily protein quantity and the redistribution pattern are supported by Brazilian and European guidelines for PD patients (Alves et al., 2023; Burgos et al., 2018).

Regarding the other macronutrients, the Acceptable Macronutrient Distribution Ranges (AMDRs) for adults will be used: carbohydrates will represent 45 to 65% and lipids 20 to 35% of the total caloric intake. For fiber, the Adequate Intake (AI) of 14 g/1000 kcal, which is based on energy consumption, will be used (Cominetti; Cozzolino, 2023).

Nutrients	Recommendations
Energy	EER (kcal/dia)
Protein	0,8 g/kg/dia
Carbohydrates	45-65% do EER
Lipids	20-35% do EER
Fiber	14g/1000 kcal

Telephone follow-up (D15 + D75)

To promote adherence to the recommendations and ensure complete follow-through, telephone calls will be made at D15 and D75 of the intervention to reinforce the guidelines and clarify doubts about the dietary plan.

Should a participant withdraw or not completely follow the protocol, the main study outcomes will be collected whenever possible, including data on constipation, diet, nutritional status, and clinical information. This data will allow for intention-to-treat analyses and reduce the impact of losses on the final outcome.

4.7 Assessments

4.7.1 Constipation

The evaluation of constipation will be performed using different instruments with the objective of capturing both subjective and objective aspects of bowel function:

1. Evacuation Diary

All participants will receive an evacuation diary, which must be filled out daily during the study period. This instrument will allow monitoring the evolution of constipation symptoms throughout the intervention weeks. The registered information includes:

- Number of evacuations per week (evacuation frequency);
- Stool consistency using the Bristol Stool Scale, a validated visual tool that classifies stools into seven types. Types 1 and 2 will be considered hard stools, and their percentage will be calculated over time;
- Straining during evacuation (report of need to strain to evacuate);
- Sensation of incomplete evacuation;

- Use of rescue laxative (in case of absence of spontaneous evacuation).

2. *Constipation Scoring System* (CSS)

The CSS will be applied at baseline, D45, and D90. This questionnaire consists of eight items and assesses the severity of functional constipation in adults, considering aspects such as frequency, effort, feeling of incomplete evacuation, and time spent on the toilet. Its total score allows for quantifying the intensity of the symptoms, assisting in monitoring the response to the intervention. The tool was translated, culturally adapted, and validated for the Brazilian population, demonstrating good comprehension by the patients and high psychometric reliability (Taniguchi et al., 2022).

4.7.2 Gut microbiota

Participants will receive instructions and the container for collecting the stool sample at baseline. The collection will be carried out by the participants themselves in their homes, following standardized instructions provided by the team. Participants will be asked to collect a portion from the inner part of the stool, using a spatula and a sterile container, and to keep the sample in a domestic refrigerator, if possible, for a maximum of 48 hours until delivery. This form of storage aims to preserve the integrity of the biological material until transport.

Transport to the laboratory will be carried out by the participant, if possible, in a maximum of one hour from the moment of removal from the refrigerator. The delivery of the sample will be arranged with the researchers, and this moment will be classified as D0. Upon arrival at the laboratory, the samples will be aliquoted into sterile microtubes and stored at -80°C until processing.

DNA extraction from the stool samples will be performed using a commercial kit (QIAamp PowerFecal Pro DNA Kit). The column extraction methodology will be based on the manufacturer's bacterial DNA extraction protocol. The final extraction product will be quantified and evaluated for the quality of the genetic material in each sample, for subsequent genomic library preparation.

The DNA obtained from the stool samples will be used for amplification of the hypervariable V3-V4 region of the bacterial 16S rRNA gene, by polymerase chain reaction (PCR), based on the 16S Metagenomic Sequencing Library Preparation Illumina® protocol. PCR will be performed with Phusion High-Fidelity PCR Master Mix with GC Buffer Enzyme (Invitrogen) and the Nextera XT Index Kit v2 Illumina will be used for DNA library preparation. All amplified PCR products will be purified using AMPure XP beads (Beckman Coulter). A negative control (molecular grade water) will be included for quality assurance. Library concentrations will be quantified using a Qubit DNA HS assay (Invitrogen) and checked for fragment distribution by a 4200 TapeStation System capillary electrophoresis instrument (Agilent). Pooled libraries will be loaded onto the MiSeq® platform using a

MiSeq™ i100 Series 25M Rgt Kit 600 cyc reagent kit. These analyses will be performed at LABRESIS/HCPA.

- Fecal antigen test for H. pylori: A fecal antigen test for H. pylori will also be performed on the stool sample. It is a non-invasive exam that detects the presence of Helicobacter pylori in the feces through specific antibodies that identify bacterial antigens. It is used for the diagnosis of active infection and to confirm eradication after treatment.

4.7.3 Diet

The dietary assessments (R24h and Screener-Nova) will be carried out with all participants at baseline, D45 (via telephone), and D90.

- 24-hour dietary recall (24hR)

The 24h recall (R24h) is a practical and widely used tool in nutritional studies to estimate habitual food intake. Its limitations, such as dependence on patient memory and representing only a single day of habitual eating, are known, meaning the R24h may not adequately reflect the variability of food consumption. However, it allows for quick collection of detailed information about the diet retrospectively, without interfering with eating habits. It is also a low-cost tool and well-accepted by participants.

Participants will be interviewed by a trained nutritionist, who will request the information in five steps: (1) quick listing of all foods and beverages consumed the day before; (2) detailed description of each item; (3) quantification using household measures and standardized utensils; (4) checking for forgotten items, such as the use of oil, condiments, and snacks; and (5) final review. Whenever possible, the use of photographs of household measures will be adopted to aid in the estimation of quantities. The collected information will later be analyzed using specific food composition software to calculate energy, macronutrients, micronutrients, and fiber.

- Diet quality

The diet will be assessed using the Screener-Nova. It is a self-administered food frequency questionnaire based on the Nova food processing classification, used to quickly monitor diet quality, with an average response time of 2 to 3 minutes, available on the QuestNova web platform, developed by the Center for Epidemiological Research in Nutrition and Health, at the University of São Paulo (NUPENS/USP) (Louzada et al., 2024). The researcher responsible for the assessment will apply the questionnaires with the participants to ensure better understanding.

The Screener-Nova is composed of seven blocks of yes/no questions, addressing food consumption from the previous day. Four of these refer to in natura or minimally processed foods

(fruits, vegetables, greens, and grains) and three assess ultra-processed foods (beverages, ready-to-eat meals, and snacks). The data automatically generate two indicators of diet quality, based on the simple sum of the items:

- Nova Whole Plant Foods (Nova-WPF), which ranges from 0 to 33 points and evaluates in natura/minimally processed foods.
- Nova Ultra-processed Foods (Nova-UPF), which ranges from 0 to 23 points and reflects the consumption of ultra-processed foods.

- Adherence to the nutritional intervention

Adherence to the nutritional intervention will be assessed through telephone calls and self-reported compliance with the guidelines, as well as the analysis of food consumption (24h recall). This monitoring will be carried out exclusively by the researcher responsible for the intervention and will be stored separately from the other data. Fiber consumption compared to the goals will be used as one of the indicators of adherence. Participants who meet at least 75% of the goals will be considered to have good adherence.

4.7.4 Nutritional status

The assessments described below will be performed on all participants at the beginning and end of the study (baseline and D90).

- Anthropometry

Anthropometric measurements will be taken following the guidelines set out in the Technical Standard of the Food and Nutrition Surveillance System (Brasil, 2011). The measurements to be collected are: weight (kg), height (cm), calf circumference (CC), arm circumference (AC) and waist circumference (WC). The weight and height of the participants will be measured using a clinical scale with a stadiometer available in the CPC's consultation rooms. Circumference measurements will be taken with an inelastic tape in centimeters. Subsequently, the Body Mass Index (BMI) will be calculated by the ratio of weight (kg) to height (cm) squared. The BMI classification will be as follows: Underweight: BMI $< 18.5kg/m^2$; Normal weight: BMI between 18.5 and $24.9kg/m^2$; Overweight: BMI between 25.0 and 29.9 kg/m^2; Obesity Grade 1: BMI between 30.0 and 34.9 kg/m^2; Obesity Grade 2: BMI between 35.0 and $39.9kg/m^2$ and Obesity Grade 3: BMI ≥ 40.0 kg/m^2 (World Health Organization (WHO), 2000).

- Body Composition

Body composition will be evaluated using multi-electrode electrical bioimpedance (BIA), a non-invasive, safe, and widely used method in clinical and nutritional studies. The exam will be performed following standardized recommendations: minimum 4 hours fast, empty bladder, absence of intense physical activity in the last 24 hours, and no consumption of alcoholic and caffeinated beverages the day before. These guidelines will be previously agreed upon with the participants when signing the IC.

BIA uses a low-intensity electrical current applied through electrodes positioned on extremities (hands and feet), allowing for the estimation of the resistance and reactance of body tissues. From these measurements, various variables related to nutritional status and body composition will be obtained, including lean mass, fat mass, body fat percentage, body cell mass, total body water, estimated basal metabolic rate, segmental distribution of lean mass and fat, estimated visceral fat percentage, and phase angle.

- Nutritional Status Screening

Nutritional screening will be performed using the Mini Nutritional Assessment - Short Form (MNA-SF). This instrument consists of six multiple-choice questions, each related to a different domain, and corresponding to a score of 0-3 points. The questions are divided into changes related to the last three months and current moment questions. They cover the themes: decreased appetite, difficulty chewing and swallowing, weight loss, acute disease, mobility, dementia, BMI, among others. If it is not possible to measure BMI, the MNA-SF allows for the use of the CC value. At the end of the questionnaire, the points are summed, which will indicate: normal nutritional status (12-14 points), at risk of malnutrition (8-11 points), and malnourished (0-7 points) (Rubenstein et al., 2001).

- Sarcopenia Assessment

Sarcopenia assessment will be performed according to the revised European consensus in 2019 (Cruz-Jentoft et al., 2019) and classified as described in Figure 4 below. Firstly, the SARC-F screening tool will be applied. This questionnaire consists of five questions that address the participant's self-perception of their muscle function. Each question can be scored from 0 to 2, and the result can reach up to 10 points. Scores ≥4 are suggestive of sarcopenia (Woo; Leung; Morley, 2014).

Afterward, handgrip strength (HGS) will be assessed with a calibrated hydraulic dynamometer. To perform the measurement, the participant must be seated with their feet firmly supported on the floor and elbows at 90º. They will then be asked to squeeze the device as hard as they can, with 3 repetitions. The highest result in kilograms from the 3 attempts will be used for the classification of low HGS: <27kg for men and <16 kg for women. Subsequently, low appendicular skeletal muscle mass will be evaluated: <20kg for men and <15kg for women, obtained through the BIA exam.

Finally, the Timed Up and Go (TUG) test will be performed, which is a simple and quick method to assess mobility, balance, and fall risk. It consists of measuring the time an individual takes to stand up from a chair, walk three meters, turn around, and return to sit down. Low performance will be considered ≥20 sec.

4.7.5 Sociodemographic data

The participants will answer a questionnaire developed by the researchers containing questions regarding personal and identification data of the participant such as age, sex, education, self-declared color, monthly family income, and about physical activity practice (APPENDIX C).

4.7.6 Clinical data

Clinical data to be collected include: time since diagnosis, age at diagnosis, comorbidities, use of Deep Brain Stimulation (DBS) at the initial assessment (baseline). Disease staging using the Hoehn and Yahr scale, medications in use, daily equivalent levodopa dose (LEDD), and the following questionnaires will be applied both at baseline and at D90.

- Movement Disorder Society - Unified Parkinson's Disease Rating Scale (MDS-UPDRS)

The severity and impact of symptoms will be assessed using the MDS-UPDRS, a revised and widely validated version of the UPDRS scale. The application will be performed by a trained professional and will include the four parts of the instrument: Part I (patient's non-motor experiences of daily living), Part II (patient's motor experiences of daily living), Part III (motor examination performed by the evaluator), and Part IV (motor complications). Each item is scored on a scale from 0 (normal) to 4 (severe), and higher scores reflect greater impairment. This comprehensive assessment will allow for a detailed characterization of the participants' clinical status.

- The Montreal Cognitive Assessment (MoCA)

This instrument is used for screening patients with mild cognitive impairment. With a maximum score of 30 points, the MoCA assesses eight cognitive domains, contemplating various tasks in each domain such as: executive functions, visuospatial skills, naming, memory retrieval, digits, sentence repetition, abstract reasoning, and orientation. Its application takes about 15 minutes, and individuals who score more than 26 points will be classified as having mild cognitive impairment (Tumas et al., 2016).

- Swallowing Disturbance Questionnaire (SDQ)

The Swallowing Disturbance Questionnaire (SDQ) is a screening instrument developed to identify the risk of dysphagia in people with PD. It is composed of 15 questions that address symptoms

related to the oral and pharyngeal phases of swallowing. Fourteen items are answered on a scale of 0 to 3 (0 = never; 1 = rarely; 2 = frequently; 3 = very frequently), and one item is a dichotomous response (yes/no). The total score ranges from 0 to 45 points, with scores equal to or greater than 11 indicating a risk of dysphagia. The questionnaire has been translated and validated for Brazilian Portuguese, showing good reliability properties (Ayres et al., 2016).

4.8 Statistical Analysis

Individuals will be identified through a number to maintain data confidentiality, and all collected information will be typed into the Microsoft Office Excel® spreadsheet which will be stored on the institutional Google Drive®. Only the responsible researchers will have access to the data. After tabulation, the database will be exported to the SPSS v18.0 software to perform the statistical analysis. Descriptive data analysis will be performed according to the characteristics of each variable: mean and standard deviation or median and interquartile range for quantitative variables, and frequency and percentage for categorical variables.

Intent-to-treat (ITT) analyses will be performed for all outcomes. Primary and secondary outcomes will be analyzed using generalized linear mixed models (GLMM) with group, time, and their interaction as fixed effects, and random intercepts by participants. Age, sex, disease duration, and LEED will be included as baseline covariates in all models. Weekly laxative use will be included as a time-varying covariate to adjust for its potential confounding effect on bowel movement frequency. Missing data will be handled through the likelihood-based estimation inherent to GLMM, which provides valid estimates under the missing at random (MAR) assumption. Sensitivity analyses will be conducted to assess the robustness of results to potential departures from the MAR assumption.

Regarding the microbiota, alpha diversity (α-diversity) will be evaluated by the Shannon index and statistical significance will be verified by linear modeling for mixed effects. Beta diversity (β-diversity) will be analyzed using Principal Component Analysis (PCA), Principal Coordinate Analysis (PCoA), and Non-metric Multidimensional Scaling (NMDS), all based on Aitchison (Aitchison, 1982) and Jaccard (Jaccard, 1908) distances. Statistical significance and the proportion of explained variance will be assessed using Permutational Multivariate Analysis of Variance (PERMANOVA) (Anderson, 2014) and linear models for mixed effects.

Differential abundance analyses will be performed using the Microbiome Multivariable Association with Linear Models 2 (Maaslin2) algorithm (Mallick et al., 2021). For multiple testing correction, the Benjamini-Hochberg (BH) method (Benjamini; Hochberg, 1995) will be applied to control the False Discovery Rate (FDR), as well as a mixed directional FDR control (mdFDR) will be used, with the application of the Family-Wise Error Rate control procedure by the Holm method (Holm, 1979), considering a significance level (α) of 0.05.

4.9 Ethical aspects

This project was elaborated in accordance with the Guidelines and regulatory standards for research involving human beings (Resolution 466/12) and will be submitted to the Research Ethics Committee (CEP) of HCPA. The researchers declare that they know and comply with the requirements of the General Data Protection Law (Law Nº 13.709, of August 14, 2018) regarding the treatment of personal data and sensitive personal data that will be used for the execution of this research project. The potential risks and benefits of the study are discriminated in the IC (APPENDIX B). The consent process will take place through the reading and understanding of the term, with subsequent signing by the individual who wishes to participate and the researcher. Two copies will be provided, one for the participant and one for the researcher. All collected data will be stored in a database within the institutional Google Drive® (@hcpa.edu.br) of the principal investigator. This data can only be accessed by the researchers cited here as the research team. Whenever possible, data will be anonymized, especially during statistical analysis. After the data collection period, participants in the control group will receive, if benefit is demonstrated, the nutritional guidelines that make up the nutritional intervention. All participants will receive feedback, if they wish, regarding the questionnaires applied.

5. BUDGET

Materials	Quantity	Unit cost	TOTAL
Anthropometric measuring tape	3	R$ 50,00	R$ 150,00
CPC consultation room	120	R$ 7,55	R$ 906,00
Publication support for a Qualis A journal	1	R$ 3.000,00	R$ 3.000,00
Double-sided printing copies	1000	R$ 0,26	R$ 260,00
Article translation	1	R$ 300,00	R$ 300,00
ECG electrodes for use in BIA	432	R$ 0,48	R$ 207,36
Drawer for sample storage in a cold chamber	1	R$ 100,00	R$ 100,00
**Gut microbiota analysis**			**R$ 27.462,38**
DNA extraction kit - *QIAamp PowerFecal Pro DNA Kit*	2	R$ 2.885,02	R$ 5.770,04
Enzyme for genomic library preparation - *Phusion High-Fidelity PCR Master Mix with GC Buffer*	2	R$ 1.766,67	R$ 3.533,34
Indexing kit for sequencing - *Nextera XT Index Kit v2 Set A (96 indexes, 384 samples)*	1	R$ 8.319,00	R$ 8.319,00
Internal quality control for sequencing - *PhiX CONTROL V3 KIT*	1	R$ 1.577,00	R$ 1.577,00
Sequencing run cartridge - *MiSeq™ i100 Series 25M Rgt Kit 600 cyc*	1	R$ 8.263,00	R$ 8.263,00
**TOTAL**			**R$ 32.385,74**

6. TIMELINE

Project phases	2025/1	2025/2	2026/1	2026/2	2027/1	2027/2	2028/1	2028/2
Project writing	X	X						
Submission to Brazil Platform		X						
Ethics Committee (CEP) approval		X						
ClinicalTrials.gov registration		X						
Data collection		X	X	X	X			
Nutritional intervention			X	X	X			
Statistical data analysis				X	X	X		
Scientific manuscript writing				X		X	X	
Publication and conference presentation of results				X	X	X	X	X
PhD thesis defense								X

REFERENCES

AITCHISON, John. The statistical analysis of compositional data. **Journal of the Royal Statistical Society: Series B (Methodological)**, v. 44, n. 2, p. 139–160, 1982.

ALVES, Juliana Tepedino Martins *et al.* Diretriz BRASPEN de terapia nutricional no paciente com doenças neurodegenerativas. **Braspen Journal**, v. 37, n. 2, Supl 2, p. 2–34, 2023.

ANDERSON, Marti J. Permutational multivariate analysis of variance (PERMANOVA). **Wiley statsref: statistics reference online**, p. 1–15, 2014.

AYRES, Annelise *et al.* Tradução e adaptação cultural do swallowing disturbance questionnaire para o português-brasileiro. **Revista CEFAC**, v. 18, p. 828–834, 2016.

BALESTRINO, Roberta; SCHAPIRA, AHV. Parkinson disease. **European journal of neurology**, v. 27, n. 1, p. 27–42, 2020.

BARICHELLA, Michela *et al.* Dietary habits and neurological features of Parkinson's disease patients: implications for practice. **Clinical nutrition**, v. 36, n. 4, p. 1054–1061, 2017.

BECK, Aaron T. Manual for the beck depression inventory-II. **(No Title)**, 1996.

BENJAMINI, Yoav; HOCHBERG, Yosef. Controlling the false discovery rate: a practical and powerful approach to multiple testing. **Journal of the Royal statistical society: series B (Methodological)**, v. 57, n. 1, p. 289–300, 1995.

BORGES, Rogério Boff *et al.* Power and Sample Size for Health Researchers: uma ferramenta para cálculo de tamanho amostral e poder do teste voltado a pesquisadores da área da saúde. **Clinical and Biomedical Research**, v. 40, n. 4, 2020.

BRASIL. Orientações para a coleta e análise de dados antropométricos em serviços de saúde: Norma Técnica do Sistema de Vigilância Alimentar e Nutricional-SISVAN. 2011.

BURGOS, Rosa *et al.* ESPEN guideline clinical nutrition in neurology. **Clinical Nutrition**, v. 37, n. 1, p. 354–396, 2018.

CIRSTEA, Mihai S. *et al.* Microbiota composition and metabolism are associated with gut function in Parkinson's disease. **Movement Disorders**, v. 35, n. 7, p. 1208–1217, 2020.

COMINETTI, Cristiane; COZZOLINO, Silvia M. Franciscato. **Recomendações de nutrientes**. 3. ed. São Paulo: International Life Sciences Institute do Brasil – ILSI Brasil, 2023.

CRUZ-JENTOFT, Alfonso J. *et al.* Sarcopenia: revised European consensus on definition and diagnosis. **Age and ageing**, v. 48, n. 1, p. 16–31, 2019.

DORSEY, E. *et al.* The emerging evidence of the Parkinson pandemic. **Journal of Parkinson's disease**, v. 8, n. s1, p. S3–S8, 2018.

DROSSMAN, Douglas A.; HASLER, William L. Rome IV—functional GI disorders: disorders of gut-brain interaction. **Gastroenterology**, v. 150, n. 6, p. 1257–1261, 2016.

DU, Yitong *et al.* Probiotics for constipation and gut microbiota in Parkinson's disease. **Parkinsonism & Related Disorders**, v. 103, p. 92–97, 2022.

DUNK, Danielle *et al.* Diet quality, sleep and quality of life in Parkinson's disease: A cross-sectional study. **Irish Journal of Medical Science (1971-)**, v. 192, n. 3, p. 1371–1380, 2023.

HATANO, Taku *et al.* Efficacy and Safety of Elobixibat in Parkinson's Disease with Chronic Constipation: CONST‐PD Study. **Movement Disorders Clinical Practice**, v. 11, n. 4, p. 352–362, 2024.

HOLM, Sture. A simple sequentially rejective multiple test procedure. **Scandinavian journal of statistics**, p. 65–70, 1979.

IBRAHIM, Azliza *et al.* Multi-strain probiotics (Hexbio) containing MCP BCMC strains improved constipation and gut motility in Parkinson's disease: A randomised controlled trial. **Plos one**, v. 15, n. 12, p. e0244680, 2020.

JACCARD, Paul. Nouvelles recherches sur la distribution florale. **Bull. Soc. Vaud. Sci. Nat.**, v. 44, p. 223–270, 1908.

KUBO, Shin-ichiro *et al.* The effects of lactulose on constipation in patients with Parkinson's disease: An exploratory pilot study. **Eneurologicalsci**, v. 35, p. 100503, 2024.

KWON, Dayoon *et al.* Diet quality and Parkinson's disease: Potential strategies for non-motor symptom management. **Parkinsonism & Related Disorders**, v. 115, p. 105816, 2023.

KWON, Dayoon *et al.* Diet and the gut microbiome in patients with Parkinson's disease. **npj Parkinson's Disease**, v. 10, n. 1, p. 89, 2024.

LOUZADA, Maria Laura da Costa *et al.* QuestNova: inovação na avaliação do consumo alimentar segundo o processamento industrial. **Revista de Saúde Pública**, v. 58, p. 38, 2024.

MALLICK, Himel *et al.* Multivariable association discovery in population-scale meta-omics studies. **PLoS computational biology**, v. 17, n. 11, p. e1009442, 2021.

MUNHOZ, Renato P. *et al.* Non-motor signs in Parkinson's disease: a review. **Arquivos de neuro-psiquiatria**, v. 73, p. 454–462, 2015.

ONDO, WG *et al.* Placebo-controlled trial of lubiprostone for constipation associated with Parkinson disease. **Neurology**, v. 78, n. 21, p. 1650–1654, 2012.

PARKINSON STUDY GROUP. A randomized trial of relamorelin for constipation in Parkinson's disease (MOVE-PD): trial results and lessons learned. **Parkinsonism & Related Disorders**, v. 37, p. 101–105, 2017.

PEREIRA, Gabriela Magalhães *et al.* A systematic review and meta-analysis of the prevalence of Parkinson's disease in lower to upper-middle-income countries. **npj Parkinson's Disease**, v. 10, n. 1, p. 181, 2024.

PFEIFFER, Ronald F. Gastrointestinal dysfunction in Parkinson's disease. **Parkinsonism & related disorders**, v. 17, n. 1, p. 10–15, 2011.

PONT‐SUNYER, Claustre *et al.* The O nset of N onmotor S ymptoms in P arkinson's disease (T he ONSET PD S tudy). **Movement Disorders**, v. 30, n. 2, p. 229–237, 2015.

POSTUMA, Ronald B. *et al.* Prodromal autonomic symptoms and signs in Parkinson's disease and dementia with Lewy bodies. **Movement Disorders**, v. 28, n. 5, p. 597–604, 2013.

POSTUMA, Ronald B. *et al.* MDS clinical diagnostic criteria for Parkinson's disease. **Movement disorders**, v. 30, n. 12, p. 1591–1601, 2015.

POURMOTABBED, Ali *et al.* The association of ultra-processed food intake with neurodegenerative

disorders: a systematic review and dose-response meta-analysis of large-scale cohorts. **Nutritional Neuroscience**, v. 28, n. 1, p. 73–86, jan. 2025.

PRINGSHEIM, Tamara *et al.* The prevalence of Parkinson's disease: a systematic review and meta‐analysis. **Movement disorders**, v. 29, n. 13, p. 1583–1590, 2014.

REES, Joanna *et al.* A comprehensive examination of the evidence for whole of diet patterns in Parkinson's disease: a scoping review. **Nutritional neuroscience**, v. 27, n. 6, p. 547–565, 2024.

RUBENSTEIN, Laurence Z. *et al.* Screening for undernutrition in geriatric practice: developing the short-form mini-nutritional assessment (MNA-SF). **The Journals of Gerontology Series A: Biological Sciences and Medical Sciences**, v. 56, n. 6, p. M366–M372, 2001.

RUSCH, Carley *et al.* Promotion of a Mediterranean Diet Alters Constipation Symptoms and Fecal Calprotectin in People with Parkinson's Disease: A Randomized Controlled Trial. **Nutrients**, v. 16, n. 17, p. 2946, 2024.

SALIM, Safa *et al.* Gut microbiome and Parkinson's disease: Perspective on pathogenesis and treatment. **Journal of Advanced Research**, v. 50, p. 83–105, 2023.

SONG, Zheyi *et al.* Effects of ultra-processed foods on the microbiota-gut-brain axis: The bread-and-butter issue. **Food Research International**, v. 167, p. 112730, 2023.

TAN, Ai Huey *et al.* Probiotics for constipation in Parkinson disease: a randomized placebo-controlled study. **Neurology**, v. 96, n. 5, p. e772–e782, 2021.

TANIGUCHI, Thiago Masashi *et al.* Cross-cultural adaptation and validation of the Constipation Scoring System for the Brazilian population. **Arquivos de gastroenterologia**, v. 59, p. 3–8, 2022.

TOLOSA, Eduardo *et al.* Challenges in the diagnosis of Parkinson's disease. **The Lancet. Neurology**, v. 20, n. 5, p. 385–397, maio 2021.

TUMAS, Vitor *et al.* Some aspects of the validity of the Montreal Cognitive Assessment (MoCA) for evaluating cognitive impairment in Brazilian patients with Parkinson's disease. **Dementia & Neuropsychologia**, v. 10, p. 333–338, 2016.

UYAR, Gizem Özata; YILDIRAN, Hilal. A nutritional approach to microbiota in Parkinson's disease. **Bioscience of Microbiota, Food and Health**, v. 38, n. 4, p. 115–127, 2019.

WOO, Jean; LEUNG, Jason; MORLEY, John E. Validating the SARC-F: a suitable community screening tool for sarcopenia? **Journal of the American Medical Directors Association**, v. 15, n. 9, p. 630–634, 2014.

WORLD HEALTH ORGANIZATION (WHO). Obesity: preventing and managing the global epidemic: report of a WHO consultation. 2000.

YANG, Xiaodong *et al.* Effect of Lacticaseibacillus paracasei strain Shirota supplementation on clinical responses and gut microbiome in Parkinson's disease. **Food & Function**, v. 14, n. 15, p. 6828–6839, 2023.

YEMULA, Nehal *et al.* Parkinson's disease and the gut: symptoms, nutrition, and microbiota. **Journal of Parkinson's Disease**, v. 11, n. 4, p. 1491–1505, 2021.

YU, Qiu-Jin *et al.* Parkinson disease with constipation: clinical features and relevant factors. **Scientific reports**, v. 8, n. 1, p. 567, 2018.

ZANGAGLIA, Roberta *et al.* Macrogol for the treatment of constipation in Parkinson's disease. A randomized placebo‐controlled study. **Movement disorders: official journal of the Movement Disorder Society**, v. 22, n. 9, p. 1239–1244, 2007.

APPENDIX A - SPIRIT 2025 checklist

SPIRIT 2025 checklist of items to address in a randomized trial protocol*

Section / Topic	No	SPIRIT 2025 checklist item description	Reported on page no.
**Administrative information**			
Title and structured summary	1a	Title stating the trial design, population, and interventions, with identification as a protocol	1
	1b	Structured summary of trial design and methods, including items from the World Health Organization Trial Registration Data Set	4
Protocol version	2	Version date and identifier	
Roles and responsibilities	3a	Names, affiliations, and roles of protocol contributors	3
	3b	Name and contact information for the trial sponsor	-
	3c	Role of trial sponsor and funders in design, conduct, analysis, and reporting of trial; including any authority over these activities	-
	3d	Composition, roles, and responsibilities of the coordinating site, steering committee, endpoint adjudication committee, data management team, and other individuals or groups overseeing the trial, if applicable	-
**Open science**			
Trial registration	4	Name of trial registry, identifying number (with URL), and date of registration. If not yet registered, name of intended registry	
Protocol and statistical analysis plan	5	Where the trial protocol and statistical analysis plan can be accessed	
Data sharing	6	Where and how the individual de-identified participant data (including data dictionary), statistical code, and any other materials will be accessible	
Funding and conflicts of interest	7a	Sources of funding and other support (e.g., supply of drugs)	
	7b	Financial and other conflicts of interest for principal investigators and steering committee members	
Dissemination policy	8	Plans to communicate trial results to participants, healthcare professionals, the public, and other relevant groups (e.g., reporting in trial registry, plain language summary, publication)	
**Introduction**			
Background and rationale	9a	Scientific background and rationale, including summary of relevant studies (published and unpublished) examining benefits and harms for each intervention	8 -10
	9b	Explanation for choice of comparator	16
Objectives	10	Specific objectives related to benefits and harms	12
**Methods: Patient and public involvement, trial design**			
Patient and public involvement	11	Details of, or plans for, patient or public involvement in the design, conduct, and reporting of the trial	35
Trial design	12	Description of trial design including type of trial (e.g., parallel group, crossover), allocation ratio, and framework (e.g., superiority, equivalence, non-inferiority, exploratory)	12
**Methods: Participants, interventions, and outcomes**			
Trial setting	13	Settings (e.g., community, hospital) and locations (e.g., countries, sites) where the trial will be conducted	12
Eligibility criteria	14a	Eligibility criteria for participants	13
	14b	If applicable, eligibility criteria for sites and for individuals who will deliver the interventions (e.g., surgeons, physiotherapists)	-
Intervention and comparator	15a	Intervention and comparator with sufficient details to allow replication including how, when, and by whom they will be administered. If relevant, where additional materials describing the intervention and comparator (e.g., intervention manual) can be accessed	15 - 18
	15b	Criteria for discontinuing or modifying allocated intervention/comparator for a trial participant (e.g., drug dose change in response to harms, participant request, or improving/worsening disease)	-
	15c	Strategies to improve adherence to intervention/comparator protocols, if applicable, and any procedures for monitoring adherence (e.g., drug tablet return, sessions attended)	22
	15d	Concomitant care that is permitted or prohibited during the trial	13
Outcomes	16	Primary and secondary outcomes, including the specific measurement variable (e.g., systolic blood pressure), analysis metric (e.g., change from baseline, final value, time to event), method of aggregation (e.g., median, proportion), and time point for each outcome	14; 18 - 25
Harms	17	How harms are defined and will be assessed (e.g., systematically, non-systematically)	-
Participant timeline	18	Time schedule of enrollment, interventions (including any run-ins and washouts), assessments, and visits for participants. A schematic diagram is highly recommended (see Figure)	15
Sample size	19	How sample size was determined, including all assumptions supporting the sample size calculation	14
Recruitment	20	Strategies for achieving adequate participant enrollment to reach target sample size	12
**Methods: Assignment of interventions**			
Randomization:			
Sequence generation	21a	Who will generate the random allocation sequence and the method used	14
	21b	Type of randomization (simple or restricted) and details of any factors for stratification. To reduce predictability of a random sequence, other details of any planned restriction (e.g., blocking) should be provided in a separate document that is unavailable to those who enroll participants or assign interventions	14
Allocation concealment mechanism	22	Mechanism used to implement the random allocation sequence (e.g., central computer/telephone; sequentially numbered, opaque, sealed containers), describing any steps to conceal the sequence until interventions are assigned	14
Implementation	23	Whether the personnel who will enroll and those who will assign participants to the interventions will have access to the random allocation sequence	14
Blinding	24a	Who will be blinded after assignment to interventions (e.g., participants, care providers, outcome assessors, data analysts)	14

	24b	If blinded, how blinding will be achieved and description of the similarity of interventions	14
	24c	If blinded, circumstances under which unblinding is permissible, and procedure for revealing a participant's allocated intervention during the trial	-
**Methods: Data collection, management, and analysis**			
Data collection methods	25a	Plans for assessment and collection of trial data, including any related processes to promote data quality (e.g., duplicate measurements, training of assessors) and a description of trial instruments (e.g., questionnaires, laboratory tests) along with their reliability and validity, if known. Reference to where data collection forms can be accessed, if not in the protocol	18 - 25
	25b	Plans to promote participant retention and complete follow-up, including list of any outcome data to be collected for participants who discontinue or deviate from intervention protocols	18
Data management	26	Plans for data entry, coding, security, and storage, including any related processes to promote data quality (e.g., double data entry; range checks for data values). Reference to where details of data management procedures can be accessed, if not in the protocol	25
Statistical methods	27a	Statistical methods used to compare groups for primary and secondary outcomes, including harms	25 - 26
	27b	Definition of who will be included in each analysis (e.g., all randomized participants), and in which group	25 - 26
	27c	How missing data will be handled in the analysis	27
	27d	Methods for any additional analyses (e.g., subgroup and sensitivity analyses)	26
**Methods: Monitoring**			
Data monitoring committee	28a	Composition of data monitoring committee (DMC); summary of its role and reporting structure; statement of whether it is independent from the sponsor and funder; conflicts of interest and reference to where further details about its charter can be found, if not in the protocol. Alternatively, an explanation of why a DMC is not needed	
	28b	Explanation of any interim analyses and stopping guidelines, including who will have access to these interim results and make the final decision to terminate the trial	
Trial monitoring	29	Frequency and procedures for monitoring trial conduct. If there is no monitoring, give explanation	
**Ethics**			
Research ethics approval	30	Plans for seeking research ethics committee/institutional review board approval	27
Protocol amendments	31	Plans for communicating important protocol modifications to relevant parties	27
Consent or assent	32a	Who will obtain informed consent or assent from potential trial participants or authorized proxies, and how	35
	32b	Additional consent provisions for collection and use of participant data and biological specimens in ancillary studies, if applicable	-
Confidentiality	33	How personal information about potential and enrolled participants will be collected, shared, and maintained in order to protect confidentiality before, during, and after the trial	27
Ancillary and post-trial care	34	Provisions, if any, for ancillary and post-trial care, and for compensation to those who suffer harm from trial participation	35

*We strongly recommend reading this checklist in conjunction with the SPIRIT 2025 Explanation and Elaboration and the SPIRIT 2025 Expanded Checklist for important clarifications on all the items. We also recommend reading relevant SPIRIT extensions. See www.consort-spirit.org

Citation: Chan A-W, Boutron I, Hopewell S, Moher D, Schulz KF, et al. SPIRIT 2025 statement: updated guideline for protocols of randomised trials. BMJ 2025;389:e081477. https://dx.doi.org/10.1136/bmj-2024-081477

© 2025 Chan A-W et al. This is an Open Access article distributed under the terms of the Creative Commons Attribution License (https://creativecommons.org/licenses/by/4.0/), which permits unrestricted use, distribution, and reproduction in any medium, provided the original work is properly cited.

APPENDIX B - Informed Consent Form

Project No. in Dipe 2025-0374

Project Title: "Nutritional intervention for constipation symptoms in patients with Parkinson's Disease: Randomized Clinical Trial - *The NUTRI-GUT-PD Study*"

**Researcher Name**	**Researcher Contact**
Nut. Juliana Heitich Brendler Prof.<br>Dr. Maira Rozenfeld Olchik	(51) 98913-7460<br>(51) 3308-3020
**Ethics Committee**	**CEP-HCPA Contact**
Research Ethics Committee of the Hospital de Clínicas de Porto Alegre (CEP-HCPA)	E-mail: cep@hcpa.edu.br, Phone: (51) 33596246 Address: Av. Protásio Alves, 211 – room 5068, Gate 4 - 5th floor of Block C - Rio Branco - Porto Alegre/RS. Monday to Friday, 8 am to 5 pm

You are being invited to participate in a research study that aims to evaluate whether monitoring by a nutritionist helps to improve constipation in people with Parkinson's Disease. Constipation is also known as being "bound" or having difficulty going to the bathroom (pooping) regularly. This research is being carried out by the Degenerative Adult Speech Therapy Research Group of the Neurology Service of the Hospital de Clínicas de Porto Alegre (HCPA).

If you agree to participate, we will conduct some assessments and questionnaires with you in person at the Clinical Research Center of the HCPA (CPC-HCPA). These assessments will be done at the beginning of the study and again after 3 months.

What will be done:

1. **Stool collection:** You will collect a stool sample (poop) at home, using a special container that will be provided along with all instructions. This sample will be used to analyze your intestinal health, observing the "microscopic bugs" (like bacteria) that live in the intestine and help it function well (gut microbiota). Afterward, you will deliver the sample at the location and time agreed upon with the team. The collection is simple, painless, and very important for the study.
2. **Diet evaluation:** We will ask you to tell us everything you ate the previous day. This will help us better understand the quality of your diet.
3. **Sociodemographic questionnaire:** You will fill out a questionnaire with personal information, such as education, income, race/ethnicity, and physical activity level.

4. **Clinical data:** We would also like your authorization to access your medical records and consult information about your history of Parkinson's disease, other illnesses, which medications you use, and symptoms you reported to your doctor at your last visit.
5. **Body measurements:** We will measure your weight, height, and the circumference of your calf, arm, waist, and hips.
6. **Body composition exam:** We will evaluate the amount of muscle, fat, and water in your body using an exam called bioelectrical impedance (BIA). For this, we will ask you to take off your shoes and socks and step on a device, holding two handles with your hands. The exam is fast, painless, and non-invasive. During the exam, you will stand with your arms away from your body.
7. **Strength evaluation:** We will do a quick questionnaire about your strength and, then, measure the strength of your hands with a device called a dynamometer. For this, you will sit with your feet on the floor and your arm bent. You will squeeze the device hard, three times.
8. **Mobility evaluation:** We will measure the time it takes for you to stand up from a chair, walk three meters, turn around, and sit down again.
9. **Motor evaluation:** A trained professional will apply an assessment that measures the motor symptoms of Parkinson's Disease. This assessment is done through the observation of movements and some simple tasks. The procedure is fast, painless, and non-invasive.
10. **Other questionnaires:** You will also answer questionnaires about memory and the risk of having difficulty swallowing (dysphagia).

Group division: Participants will be divided into two groups by a lottery draw:

- **Group 1:** Will receive nutritional guidance and a diet plan in a consultation with a nutritionist at the beginning of the study. Telephone calls will also be made every 15 days during the 3 months.
- **Group 2:** Will not receive guidance at the beginning, but after 3 months, they will also have the consultation with a nutritionist and receive the same guidance. Telephone calls will also be made every 15 days during the 3 months.

There are no known risks to participating in the research. There may be discomfort due to the response time for the various questionnaires and assessments; each visit to the CPC should take about 1 hour and 30 minutes, or due to the content of the questions, which involve aspects of your privacy. Body measurements or the walking test may also cause slight discomfort. The evaluations that will be performed are called non-invasive, that is, they simply check, without anything entering your body.

The possible benefits resulting from participating in the research are related to the guidance that will be provided and the exams performed. By participating, you will receive a detailed evaluation of your diet and will be able to better understand your eating habits, in addition to receiving nutritional guidance from a nutritionist (at the beginning or end of the study). You will also receive information about your body composition (how much muscle, fat, and water you have). Finally, your participation will contribute to a better understanding of the relationship between diet and health in people with Parkinson's Disease. This may contribute to improving care and nutritional guidance for other people in the future.

Your participation in the research is completely voluntary, that is, it is not mandatory. If you decide not to participate, or even, decide to withdraw from participating and withdraw your consent, there will be no prejudice to the care you receive or may come to receive at the institution.

There is no provision for any type of payment for your participation in the research, and you will not have any costs regarding the procedures involved.

The data collected during the research will always be treated confidentially. The results will be presented collectively, without the identification of the participants, that is, your name will not appear in the publication of the results.

The biological material collected in the stool sample will be stored in a coded manner. After the analyses planned in this project are carried out, the samples will be stored. This material, in addition to being used in this study, may be used in other future studies by our group.

In this case, a new research project will be submitted for the appreciation of the Research Ethics Committee and you may be called to re-consent to the use of the material.

( ) I authorize my samples to be stored for future research.

( ) I do not authorize my samples to be stored for future research.

If you have any questions, you can contact the responsible researcher or the CEP-HCPA.

This Form is signed in two copies, one for the participant and one for the researchers.

Participant's Name

___________________________________ Local: ____________

Signature Date: / /

Name of the researcher who applied the Form

____________________________________ Local: ____________

Signature Date:

APPENDIX C - Sociodemographic Questionnaire

SOCIODEMOGRAPHIC QUESTIONNAIRE

HCPA Medical Record No.:________________ Date: ____ /____ / ____

GENERAL DATA

Patient's name: ____

Email: ____

Age: _____ years Date of Birth:____/___ Sex: ( ) M ( ) F

Landline: ( ) _________________ Cell phone: ( ) ___________________

Direct contact with Patient ( ) or Caregiver ( )

Preferred contact time: ( ) morning ( ) afternoon ( ) evening | obs __________

Education level (completed up to?):_________________ Years of schooling:______

Marital status: ( ) single ( ) married ( ) widowed ( ) separated ( ) divorced

Self-declared race/color: ( ) White ( ) Black ( ) Mixed (Pardo) ( ) Indigenous ( ) Asian (Yellow)

Monthly family income: ( ) 1 to 2 ( ) 2 to 4 ( ) 4 to 10 ( ) more than 10

(in minimum wages)

Has or had nutritional follow-up ( ) yes ( ) no | how long: ___________
