# Supplementary material for: Dietitian-led intervention to manage constipation in Parkinson’s disease: Study protocol for a parallel-group randomized controlled trial (NUTRI-GUT-PD)
Source: PLoS One. 2026 Jul 27;21(7):e0354608. doi: 10.1371/journal.pone.0354608 (PMC13405298; doi:10.1371/journal.pone.0354608)
Supplement: Attachment — Submitted filename: PONE-D-26-05353_reviewer (2).pdf [file pone.0354608.s005.pdf]

PLOS One

Dietitian-led Intervention to Manage Constipation in Parkinson's Disease: Study Protocol for a Parallel-Group Randomized Controlled Trial (NUTRI-GUT-PD)

--Manuscript Draft--

**Manuscript Number:**	PONE-D-26-05353
**Article Type:**	Study Protocol
**Full Title:**	Dietitian-led Intervention to Manage Constipation in Parkinson's Disease: Study Protocol for a Parallel-Group Randomized Controlled Trial (NUTRI-GUT-PD)
**Short Title:**	NUTRI-GUT-PD: Dietitian-Led Intervention for Constipation in Parkinson's Disease
**Corresponding Author:**	MAIRA ROZENFELD OLCHIK<br>UFRGS: Universidade Federal do Rio Grande do Sul<br>Porto Alegre, RS BRAZIL
**Keywords:**	Parkinson Disease; constipation; Dietary intervention; Nutrition counseling; Randomized controlled trial; study protocol
**Abstract:**	Background: Constipation is a frequent non-motor symptom in Parkinson's Disease (PD). Standard care includes dietary and lifestyle guidance or the use of laxatives. Diet represents a promising non-pharmacological approach, but its management in PD is complex and goes beyond simple dietary recommendations. This study aims to examine whether nutritional counseling delivered by a dietitian is more effective than usual care in reducing constipation symptoms in PD patients.<br>Methods: This 90-day randomized controlled superiority trial uses a parallel-group design. A total of 54 outpatients with PD without dementia and fulfilling the Rome IV criteria for functional constipation will be included. Baseline assessments comprise demographics, nutritional evaluation (anthropometric measurements, food frequency questionnaire, and sarcopenia indicators), neurological assessment and the Constipation Scoring System scores. Participants will perform stool sample collection for microbiota analysis and will be requested to complete three 24-hour dietary recalls over the subsequent week. After baseline, participants will be randomly allocated to either the control group (usual care) or the intervention group (nutritional counseling delivered by a dietitian). At the first study visit, participants will provide the stool sample and dietary recalls. The intervention will last for 90 days and will include two in-person consultations and bi-weekly follow-up phone calls. Nutritional counseling will address four main topics: healthy eating, food processing, fiber and fluid intake, and levodopa interactions with diet. In addition, participants in the intervention group will receive an individualized diet plan. All baseline assessments will be repeated at the 90-day endpoint. The primary outcome is the change in weekly bowel movement frequency from baseline to day 90. Secondary outcomes include changes in fecal gut microbiota, macronutrient distribution, diet quality, body composition, sarcopenia indicators, and non-motor symptoms scale. The study has obtained approval from the Hospital's ethics committee. Trial registration number: NCT07213856.
**Order of Authors:**	Juliana Heitich Brendler, MSc
	Khadija Younes da Silva
	Andreza Francisco Martins, PhD
	Valesca Dall Alba, PhD
	Daniel Teixeira-dos-Santos, MSc
	Artur Schumacher Schuh, PhD
	Maira Rozenfeld Olchik, PhD
**Additional Information:**	
**Question**	**Response**
**Financial Disclosure**	Yes

Powered by Editorial Manager® and ProduXion Manager® from Aries Systems Corporation

Enter a financial disclosure statement that describes the sources of funding for the work included in this submission. Review the submission guidelines for detailed requirements. View published research articles from *PLOS ONE* for specific examples.<br><br>This statement is required for submission and **will appear in the published article** if the submission is accepted. Please make sure it is accurate.<br><br>**Funded studies**<br>Enter a statement with the following details:<br>• Initials of the authors who received each award<br>• Grant numbers awarded to each author<br>• The full name of each funder<br>• URL of each funder website<br>• Did the sponsors or funders play any role in the study design, data collection and analysis, decision to publish, or preparation of the manuscript?<br><br>Did you receive funding for this work?	
Please add funding details.<br>as follow-up to "**Financial Disclosure**<br><br>Enter a financial disclosure statement that describes the sources of funding for the work included in this submission. Review the submission guidelines for detailed requirements. View published research articles from *PLOS ONE* for specific examples.<br><br>This statement is required for submission and **will appear in the published article** if the submission is accepted. Please make sure it is accurate.	This study was financed in part by the Coordenação de Aperfeiçoamento de Pessoal de Nível Superior – Brasil (CAPES) (Finance Code 001).<br>The doctoral scholarship was awarded to JHB.<br>Funder website: https://www.gov.br/capes<br><br>This funding represents general PhD funding and the funder had no role in the study design; data collection, analysis, or interpretation; decision to publish; or preparation of the manuscript.

**Funded studies**<br>Enter a statement with the following details:<br>• Initials of the authors who received each award<br>• Grant numbers awarded to each author<br>• The full name of each funder<br>• URL of each funder website<br>• Did the sponsors or funders play any role in the study design, data collection and analysis, decision to publish, or preparation of the manuscript?<br><br>Did you receive funding for this work?"	
Please select the country of your main research funder (please select carefully as in some cases this is used in fee calculation).<br>as follow-up to "**Financial Disclosure**<br><br>Enter a financial disclosure statement that describes the sources of funding for the work included in this submission. Review the submission guidelines for detailed requirements. View published research articles from *PLOS ONE* for specific examples.<br><br>This statement is required for submission and **will appear in the published article** if the submission is accepted. Please make sure it is accurate.<br><br>**Funded studies**<br>Enter a statement with the following details:<br>• Initials of the authors who received each award<br>• Grant numbers awarded to each author<br>• The full name of each funder<br>• URL of each funder website<br>• Did the sponsors or funders play any role in the study design, data collection and analysis, decision to publish, or preparation of the manuscript?<br><br>Did you receive funding for this work?"	BRAZIL - BR
**Competing Interests**	The authors have declared that no competing interests exist.

Use the instructions below to enter a competing interest statement for this submission. On behalf of all authors, disclose any competing interests that could be perceived to bias this work—acknowledging all financial support and any other relevant financial or non-financial competing interests.<br><br>This statement is required for submission and **will appear in the published article** if the submission is accepted. Please make sure it is accurate and that any funding sources listed in your Funding Information later in the submission form are also declared in your Financial Disclosure statement.<br><br>View published research articles from *PLOS ONE* for specific examples.<br><br>**NO authors have competing interests**<br><br>Enter: *The authors have declared that no competing interests exist.*<br><br>**Authors with competing interests**<br><br>Enter competing interest details beginning with this statement:<br><br>*I have read the journal's policy and the authors of this manuscript have the following competing interests: [insert competing interests here]*<br><br>* typeset	
**Ethics Statement**<br><br>Enter an ethics statement for this submission. This statement is required if the study involved:<br><br>• Human participants<br>• Human specimens or tissue<br>• Vertebrate animals or cephalopods<br>• Vertebrate embryos or tissues	This study will involve human participants and has been reviewed and approved by the Research Ethics Committee of the Hospital de Clínicas de Porto Alegre (HCPA), Brazil (protocol number 2025-0374). The trial is planned and will be conducted in accordance with the Declaration of Helsinki and applicable national regulations. All participants will provide written informed consent prior to enrollment. Participant confidentiality and privacy will be strictly maintained throughout the study, and all data will be de-identified for analysis and reporting.

Powered by Editorial Manager® and ProduXion Manager® from Aries Systems Corporation

• Field research<br><br>Write "N/A" if the submission does not require an ethics statement.<br><br>General guidance is provided below. Consult the submission guidelines for detailed instructions. **Make sure that all information entered here is included in the Methods section of the manuscript.**	

**Format for specific study types**<br><br>**Human Subject Research (involving human participants and/or tissue)**<br>• Give the name of the institutional review board or ethics committee that approved the study<br>• Include the approval number and/or a statement indicating approval of this research<br>• Indicate the form of consent obtained (written/oral) or the reason that consent was not obtained (e.g. the data were analyzed anonymously)<br><br>**Animal Research (involving vertebrate animals, embryos or tissues)**<br>• Provide the name of the Institutional Animal Care and Use Committee (IACUC) or other relevant ethics board that reviewed the study protocol, and indicate whether they approved this research or granted a formal waiver of ethical approval<br>• Include an approval number if one was obtained<br>• If the study involved *non-human primates*, add *additional details* about animal welfare and steps taken to ameliorate suffering<br>• If anesthesia, euthanasia, or any kind of animal sacrifice is part of the study, include briefly which substances and/or methods were applied<br><br>**Field Research**<br><br>Include the following details if this study involves the collection of plant, animal, or other materials from a natural setting:<br>• Field permit number<br>• Name of the institution or relevant body that granted permission	
**Data Availability**<br><br>Authors are required to make the data underlying their research findings fully available, without restriction.<br><br>If the manuscript reports pilot or preliminary results, the data underlying those results must be made available at the time of publication. A Data Availability	N/A - No results are reported

Powered by Editorial Manager® and ProduXion Manager® from Aries Systems Corporation

Statement describing where the data can be found is required at submission. Your answers to this question constitute the Data Availability Statement and will be published in the article, if accepted. When legal or ethical requirements prohibit public data sharing, authors must indicate how researchers may obtain access to the data. See the PLOS Data Policy and FAQ for detailed information.<br><br>**Important:** Stating 'data available on request from the author' is not sufficient. If your data are only available upon request, select 'No' for the first question and explain your situation in the text box.<br><br>For protocols without pilot or preliminary data, authors are strongly encouraged to state how they plan to share research data from their study when it is completed or published.<br><br>If your study includes pilot data, will they be made fully available without restriction?	
**Describe where the data may be found in full sentences. If you are copying our sample text, replace any instances of XXX with the appropriate details.**<br><br>• If **no pilot data are reported** in the manuscript, please enter the following: *No datasets were generated or analysed during the current study. All relevant data from this study will be made available upon study completion.*<br>• If the manuscript reports pilot data **held in a public repository**, include URLs, accession numbers or DOIs. If this information will only be available after acceptance, indicate this by ticking the box below. For example: *All XXX files are available from the XXX database (accession number(s) XXX, XXX.).*<br>• If the manuscript reports pilot data contained **within the manuscript and/or**	No datasets were generated or analysed during the current study. All relevant data from this study will be made available upon study completion.

Powered by Editorial Manager® and ProduXion Manager® from Aries Systems Corporation

**Supporting Information files**, enter the following:<br>*All relevant data are within the manuscript and its Supporting Information files.*<br>• If neither of these applies but you are able to provide **details of access elsewhere**, with or without limitations, please do so. For example:<br><br>*Data cannot be shared publicly because of [XXX]. Data are available from the XXX Institutional Data Access / Ethics Committee (contact via XXX) for researchers who meet the criteria for access to confidential data.*<br><br>*The data underlying the results presented in the study are available from (include the name of the third party and contact information or URL).*<br>• This text is appropriate if the data are owned by a third party and authors do not have permission to share the data.<br><br>For study protocols with data management or sharing plans, authors are encouraged to briefly describe in their Data Availability Statement how data they generate will be made accessible when the study is completed. For example:<br>• Deidentified research data will be made publicly available when the study is completed and published.<br><br>* typeset	
Additional data availability information:	

Manuscript
Click here to access/download;Manuscript;Manuscript.docx

Dietitian-led Intervention to Manage Constipation in Parkinson's Disease: Study Protocol for a Parallel-Group Randomized Controlled Trial (NUTRI-GUT-PD)

Juliana Heitich Brendler [1], Khadija Younes da Silva [5] ,Valesca Dall Alba [2,5], Andreza Francisco Martins [3,6], Daniel Teixeira-dos-Santos [4], Artur Francisco Schumacher Schuh [1,4], Maira Rozenfeld Olchik [1 ,4, 7*]

[1] Graduate Program in Medical Sciences, Universidade Federal do Rio Grande do Sul, Porto Alegre, RS, Brazil.
[2] Nutrition and Dietetics Division, Hospital de Clínicas de Porto Alegre, Porto Alegre, Brazil
[3] Microbiology Unit, Clinical Pathology Service, Hospital de Clínicas de Porto Alegre, Porto Alegre, Brazil
[4] Neurology Service, Hospital de Clínicas de Porto Alegre (HCPA), Porto Alegre, Brazil
[5] Department of Nutrition, School of Medicine, UFRGS, Porto Alegre, Brazil
[6] Department of Microbiology, Immunology and Parasitology, Federal University of Rio Grande do Sul, Porto Alegre, RS, Brazil.
[7] Department of Surgery and Orthopedics, School of Dentistry, Universidade Federal do Rio Grande do Sul, Porto Alegre, RS, Brazil.

* Corresponding author
e-mail: molchik@hcpa.edu.br (MRO)

Abstract

Background: Constipation is a frequent non-motor symptom in Parkinson's Disease (PD). Standard care includes dietary and lifestyle guidance or the use of laxatives. Diet represents a promising non-pharmacological approach, but its management in PD is complex and goes beyond simple dietary recommendations. This study aims to examine whether nutritional counseling delivered by a dietitian is more effective than usual care in reducing constipation symptoms in PD patients. **Methods:** This 90-day randomized controlled superiority trial uses a parallel-group design. A total of 54 outpatients with PD without dementia and fulfilling the Rome IV criteria for functional constipation will be included. Baseline assessments comprise demographics, nutritional evaluation (anthropometric measurements, food frequency questionnaire, and sarcopenia indicators), neurological assessment and the Constipation Scoring System scores. Participants will perform stool sample collection for microbiota analysis and will be requested to complete three 24-hour dietary recalls over the subsequent week. After baseline, participants will be randomly allocated to either the control group (usual care) or the intervention group (nutritional counseling delivered by a dietitian). At the first study visit, participants will provide the stool sample and dietary recalls. The intervention will last for 90 days and will include two in-person consultations and bi-weekly follow-up phone calls. Nutritional counseling will address four main topics: healthy eating, food processing, fiber and fluid intake, and levodopa interactions with diet. In addition, participants in the intervention group will receive an individualized diet plan. All baseline assessments will be repeated at the 90-day endpoint. The primary outcome is the change in weekly bowel movement frequency from baseline to day 90. Secondary outcomes include changes in fecal gut microbiota, macronutrient distribution, diet quality, body composition, sarcopenia indicators, and non-motor symptoms scale. The study has obtained approval from the Hospital's ethics committee. Trial registration number: NCT07213856.

Introduction

Parkinson's Disease (PD) has seen a rapid increase in its incidence and prevalence worldwide over the last decades, in an almost pandemic manner [1]. This neurodegenerative disease can span decades and tends to accumulate disabilities over time. For people living with PD, the consequences are significant, and for society, the socioeconomic burden is straining [2]. At this time, there is still no therapy that can slow down disease progression; therefore, optimal treatment should include a multidisciplinary approach with optimized symptomatic pharmacological therapy associated with non-pharmacological strategies [3].

Constipation is one of the most prevalent non-motor symptoms in patients with PD, affecting over 80% of individuals [4], and contributing to a reduced quality of life [5]. This symptom is linked to alterations in gut motility and gastrointestinal dysregulation [6], which can precede motor symptoms by up to two decades [7], and it is a prodromal characteristic of the disease. The prolonged intestinal transit time in these patients can lead to gut microbiota dysbiosis, an imbalance of beneficial and pathogenic bacteria. Dysbiosis is linked to increased intestinal permeability, which allows microbial products to enter the bloodstream, triggering inflammation that contributes to neuroinflammation and neurodegeneration [8].

Despite its prevalence and relevance, constipation in PD is often overlooked, and the absence of robust evidence has led to a lack of a standardized approach in international guidelines [9]. Usual care typically relies on general advice regarding fiber intake and lifestyle modifications provided by the clinician, or on the use of laxatives [10]. The available treatment options remain suboptimal for managing constipation symptoms in the majority of patients [11]. Focusing on dietary interventions for constipation in PD, the current literature does not provide sufficient evidence to support strong recommendations. The Mediterranean diet may have beneficial effects on the gut microbiota [12]; however, it has shown similar outcomes to standard care in managing constipation [13], and it may not be feasible to apply in

underdeveloped countries. Psyllium fiber supplementation demonstrated positive effects on constipation symptoms, but the evidence is limited to a small sample of seven participants [14]. Dietary changes in PD patients without expert guidance may pose risks of weight loss, malnutrition, sarcopenia, and worsening abdominal symptoms [15].

Diet management in PD is multifaceted and extends beyond simple dietary advice, encompassing the management of several non-motor symptoms and nutritional complications, including constipation [16]. Nutritional interventions led by dietitians have demonstrated superior effects compared to usual care in randomized controlled trials (RCTs) and meta-analyses, particularly in improving metabolic and cardiovascular outcomes, nutritional status, and diet quality [17–19]. Given the importance of nutritional management in PD and the lack of RCTs evaluating structured dietary interventions in this population, further investigation is warranted. Therefore, this RCT is designed to investigate whether individualized nutritional counseling, provided by a dietitian, is more effective than usual care in reducing constipation symptoms and promoting healthier gut microbiota profiles in patients with PD.

Methods

This protocol is reported following the SPIRIT reporting guidelines.

Trial design

This study is a randomized, controlled, superiority trial with a parallel-group design and a duration of 90 days. It will be conducted at the Hospital de Clínicas de Porto Alegre (HCPA), Brazil. The study targets adults diagnosed with PD without dementia who are experiencing constipation. Participants will be recruited at the Movement Disorders Research Group outpatient clinic of HCPA.

Eligibility Criteria

Adults with a prior diagnosis of PD, confirmed by a neurologist according to the Movement Disorders Society clinical diagnostic criteria [20], and receiving a stable dose of levodopa for at least 3 months will be included. In addition, participants must report at least two criteria defined by the Rome IV protocol for functional constipation [21]. Exclusion criteria include: (1) atypical or secondary parkinsonism, (2) Hoehn & Yahr stage greater than 3, (3) severe neurological or psychiatric disorders that could interfere with study procedures, (4) dementia diagnosis, (5) diagnosis of active cancer, chronic obstructive pulmonary disease, heart failure, or chronic kidney disease, (6) history of gastrointestinal neoplasms, inflammatory bowel disease, or gastrointestinal surgery (7) constipation secondary to conditions such as hypothyroidism or diabetes mellitus, (8) recent opioid use, (9) use of probiotics or antibiotics within the past 30 days, or within the past 90 days if administered during hospitalization, (10) continuous daily laxative use in the last eight weeks, (11) severe dysphagia by the Functional Oral Intake Scale classification, (12) enteral or parenteral nutrition, or (13) ongoing nutritional counseling within the last 3 months.

Recruitment

Participants will be recruited from a project previously approved by the Research Ethics Committee of the HCPA, under CAAE: 25047219.0.2002.5327, and authorized by the HCPA Research Ethics Committee under approval number 2021-0576. Individuals meeting the eligibility criteria will be invited to participate in the NUTRI-GUT-PD study, either by phone or during their routine outpatient visits. Those who agree to participate will provide written informed consent, after which the baseline visit will be scheduled to initiate study procedures.

Randomization

The randomization sequence will be generated by an independent statistician using the Sealed Envelope software. Randomization will be stratified by sex (male/female). Allocation concealment will be ensured through the REDCap electronic data capture tools. Participants will be randomized sequentially in the order that they provide informed consent. Group assignment will be disclosed to participants at D0.

Initial baseline assessments will occur before randomization, eliminating the need for blinding at this stage. Outcome assessors, microbiota sequencing professionals, and the statistician will be blinded to group allocation. The dietitians delivering the intervention will not be blinded, given the nature of the counseling.

Sample Size

The required sample size is 48 participants (24 per group) to detect a minimum difference of 1 bowel movement/week between groups at day 90, assuming 80% power, 5% significance, standard deviations of 1.15 (intervention) and 1.21 (control), and a 0.5 correlation in repeated measures. Accounting for 10% attrition, 54 participants (27 per group) will be recruited. Sample size was calculated using the PSS Health online tool [22].

Outcomes

Primary Outcome

The primary outcome is the change in frequency of weekly bowel movements measured through the stool diary and Constipation Scoring System (CSS) scores from baseline to close-out.

Secondary Outcomes

Secondary outcomes will include changes from initial assessments to close-out in the fecal gut microbiota (species richness, alpha diversity, beta diversity and differential

abundance), macronutrient distribution measured through the 24h dietary recalls, diet quality assessed by the Screener-Nova, body composition (BIA and anthropometric measures), sarcopenia indicators, and non-motor symptoms.

Participant Timeline

The participant timeline is presented in Fig 1. After eligibility screening and informed consent, participants will complete baseline assessments and will then be randomized to either usual care or dietitian-led nutritional counseling. The intervention period will last 90 days, with outcome assessments repeated at Day 90.

Fig 1. Participant schedule of enrolment, interventions and assessments.
CSS: Constipation Scoring System; FFQ: Food Frequency Questionnaire; BIA: Bioelectrical Impedance Analysis. *All visit days are ±3 days. [a] Randomization will occur between baseline and D0. [b] Patients will receive stool collection kits on baseline, allowing them to bring the samples on the visit day for analysis. [c] 24-hour dietary recalls will be completed on three different days prior to each of the study visits.

At the time of submission, the study is in the pre-recruitment phase. Participant recruitment is expected to begin in March 2026 and to be completed by July 2026. Each participant will undergo a 3-month intervention period, and data collection is expected to be completed by November 2027. Data analysis is planned between December 2026 and January 2027, and study results are expected in early 2027. Study findings will be disseminated through peer-reviewed publications and conference presentations.

Data Collection

The study will include three in-person visits at baseline, D0, and D90, with a phone contact on D45. In addition, participants will receive phone calls every 15 days to promote adherence to the intervention, address potential issues, and inquire about intestinal symptoms. The assessments planned for each visit are summarized in Fig 2 and detailed below.

Fig 2. Study Design NUTRI-GUT-PD. Figure created by the author (2025). BIA: Bioelectrical Impedance Analysis; 24h DRs: 24h Dietary Recalls; FFQ: Food Frequency Questionnaire.

Considering the complexity of the proposed assessment battery and the inherent mental and physical fatigue associated with the neurodegenerative condition, several strategies will be utilized to promote participant retention and adherence throughout the trial. Participants will be offered a ±3day window for all scheduled visits and phone calls. Caregivers will be actively encouraged to attend study visits and assist with data collection, and provide logistical support to the participant. Lastly, to facilitate compliance, scheduled communication via text message or telephone calls will be established. Encompassing confirmation of visit appointments, reminders regarding BIA orientation, daily prompts to complete the stool diary, and reminders to collect and bring the stool sample and the completed diary to the scheduled visit.

Sociodemographic Data

Will be collected in the baseline visit through a standardized questionnaire including age, sex, education, self-reported ethnicity and household income.

Neurological Evaluation

Medical history, covering disease duration, age at diagnosis, comorbidities, and use of Deep Brain Stimulation (DBS), will be collected at baseline. Disease severity and duration will be assessed using the Hoehn and Yahr staging scale. Information on medication use will be collected at baseline and on D90, including the daily levodopa equivalent dose (LEED) and any concomitant treatments or constipating medications.

The study neurologist will perform a clinical assessment at baseline and D90, including MDS-Unified Parkinson’s Disease Rating Scale (MDS-UPDRS) [23], Montreal Cognitive

Assessment (MoCA) [24], Swallowing Disturbance Questionnaire (SDQ) [25], and International Physical Activity Questionnaire- Short Form (IPAQ-SF) [26].

Constipation Assessment

Constipation will be evaluated using two instruments to capture both subjective and objective aspects of bowel function. All participants will receive a stool diary to be completed daily. This tool will be used to monitor the progression of constipation symptoms throughout the study period. The following items will be recorded:

- Was evacuation present today?
    - Weekly bowel movement frequency will be calculated accordingly.
- What was the stool consistency?
    - Assessed using the Bristol Stool Form Scale. Types 1–2 will be classified as hard stools and reported as the weekly percentage of total evacuations.
- Was there a sensation of straining?
    - Responses ("Yes" or "No") will be summarized as weekly percentages.
- Was the evacuation incomplete?
    - Responses ("Yes" or "No") will be summarized as weekly percentages.
- Did you use laxatives today due to the absence of spontaneous bowel movements?
    - The weekly frequency of rescue laxative use will be calculated.

The CSS will be administered at baseline, D45, and D90. This instrument assesses intestinal dysfunction through eight items evaluating bowel movement frequency, straining, incomplete evacuation, and time spent in the bathroom. The total score ranges from 0 to 30, with higher scores indicating a worse perception of functional constipation. The culturally adapted Brazilian version has demonstrated strong comprehension and psychometric reliability[27].

Gut Microbiota

At baseline, participants will receive standardized instructions and two sterile containers labeled with their name and the collection date, to collect stool samples at home on D0 and D90. Participants will be reminded during the visit and through follow-up messages about the instructions for proper at-home stool sample collection.

Samples must be stored refrigerated (up to 48h) and delivered to the research team, preferably within 1 hour of removal from refrigeration. Upon receipt at the laboratory, samples will be aliquoted into sterile microtubes and stored at –80 °C until analysis.

- DNA Extraction: The DNA will be extracted from 250mg of the sample using QIAamp PowerFecal Pro DNA Kit following the manufacturer's protocol. DNA yield and quality will be verified before sequencing.
- 16S rRNA Sequencing: The V3–V4 hypervariable region will be amplified by PCR using the Illumina® 16S Metagenomic Sequencing Library Preparation protocol. Amplified products will be purified with AMPure XP beads, quantified using Qubit DNA HS assays, and fragment distribution assessed with the TapeStation 4200 system. Libraries will be pooled and sequenced on the MiSeq® platform with the MiSeq™ i100 Series 25M Reagent Kit (600 cycles). A negative control (molecular-grade water) will be included in each run.

Dietary Assessments

To assess habitual dietary patterns before the intervention, a Food Frequency Questionnaire (FFQ) will be utilized. The FFQ is a dietary assessment tool that estimates usual food and nutrient intake based on the reported frequency of consumption of listed food items over the previous year. The version adapted for the population of Porto Alegre [28] (validated for adult and elderly populations) will be administered at baseline. Nutrient composition will be calculated using the Brazilian Food Composition Table (TACO). The extracted variables

will include total daily energy intake (kcal), macronutrient distribution (protein, fat, carbohydrate, and fiber as percentages of total energy), and the proportion of food consumption by NOVA classification categories (unprocessed/minimally processed, processed, and ultra-processed foods).

Before each assessment point (D0, D45, and D90), participants will complete three 24-hour dietary recalls (two weekdays and one weekend day). To ensure accuracy, two recalls will be conducted by telephone during the week preceding the visit, and the third will be completed in person at the visit, referring to the day immediately before. Standardized multiple-pass methodology will be used: quick listing of foods, detailed descriptions, portion size estimation with household measures, checking for forgotten items (oil, condiments, snacks), and final review. Data will be entered into nutrient analysis software to estimate energy, macronutrient, micronutrient, and fiber intake. Each food recall will also be entered by the nutritionist into the R24h-Nova [29] to identify the percentage of energy from each Nova classification group.

To evaluate diet quality the Screener-NOVA [29] will be implemented. It is a brief, web-based questionnaire designed to assess diet quality according to the NOVA food classification system. It consists of seven yes/no sections addressing the consumption of unprocessed or minimally processed foods (fruits, vegetables, legumes, grains) and ultra-processed foods (sugar-sweetened beverages, ready-to-eat meals, snacks). The questionnaire will be administered at D0, D45, and D90 with support from the dietitian. It generates two indicators: the NOVA-WPF (Whole Plant Foods) score, ranging from 0 to 33 points, where higher values indicate greater consumption of WPF; and the NOVA-UPF (Ultra-Processed Foods) score, ranging from 0 to 23 points, where higher values indicate greater consumption of UPF. For classification, a NOVA-WPF score of ≥ 10 points will be used to define desirable consumption, and a NOVA-UPF score of < 4 points will be used to define non-elevated consumption [30].

Nutritional Status

Anthropometry will consist of weight, height, calf circumference, arm circumference, and waist circumference, measured according to the national guidelines [31] for anthropometrics measurements at baseline and D90. BMI will be calculated and classified according to the World Health Organization [32] categories.

Bioelectrical Impedance Analysis (BIA) with multiple electrodes will be performed in the first (D0) and last visits (D90). It estimates lean mass, fat mass, fat percentage, cellular mass, total body water, basal metabolic rate, segmental distribution, visceral fat, and phase angle. Participants will follow standardized pre-test conditions (≥4h fasting, empty bladder, no intense physical activity for 24h, no alcohol/caffeine the previous day).

The Mini Nutritional Assessment – Short Form (MNA-SF)[33] will be applied by a trained dietitian at D0 and D90 of the study to assess malnutrition risk. Scores classify participants as well-nourished (12–14), at risk of malnutrition (8–11), or malnourished (0–7).

Sarcopenia Assessment

Sarcopenia assessment will be done in the baseline visit and also at the end point of the study (D90). It will follow the revised 2019 European consensus [34]:

- Screening: SARC-F questionnaire (≥4 indicates probable sarcopenia).
- Muscle Strength: Handgrip strength via calibrated dynamometer (<27 kg men; <16 kg women = low strength).
- Muscle Mass: Appendicular skeletal muscle mass (BIA; <20 kg men, <15 kg women = low mass).
- Physical Performance: Timed Up and Go (TUG) test; ≥20 s indicates poor performance.

Interventions

Intervention Group

The nutritional intervention is aimed at improving diet quality, constipation symptoms, and gut health. The intervention is based on recommendations from the Brazilian and European consensus for neurological diseases [35,36] and the Food Guide for the Brazilian Population [37]. The intervention will last 90 days, with an initial in-person visit at the research center followed by biweekly telephone contacts until the final assessment, totaling one onsite visit and five phone calls between the participants and the study's dietitian. Participants in the intervention group will receive individual nutritional counseling sessions, a standardized manual on the covered topics (S2 Appendix) and a personalized diet plan elaborated by a dietitian specialized in neurology and elderly health.

During the first visit (D0), the dietitian will assess participants' prior knowledge and provide counseling on four key topics: levels of food processing, healthy eating, the importance of fiber and fluid intake, and interactions between levodopa and nutrients (Table 1). Individual goals will be established collaboratively with each participant during the first visit and reinforced throughout the study period (e.g., increasing daily fruit intake, reducing added sugar in coffee, or replacing ultra-processed foods with minimally processed alternatives). This section of the visit will last approximately one hour. Participants will also receive a standardized healthy eating manual developed specifically for this trial, which was reviewed by faculty members from the fields of nutrition, medicine, speech therapy, and pharmacy. The manual covers the four key topics to ensure consistency in the information provided across the group.

Table 1. Nutritional counseling framework for the intervention.

**Nutritional Counseling Topics**	**Description**
Food Processing	- Definition and examples of minimally processed, processed, and ultra-processed foods.<br>- Practical identification of UPFs in daily life.
Healthy Eating	- Preference for natural or minimally processed foods.<br>- Moderate use of oils, fats, salt, and added sugars.<br>- How to assemble a balanced plate.<br>- How to read food labels.
Fiber and Fluid Intake	- Types of dietary fiber (soluble and insoluble) and their natural food sources.<br>- Role of fiber in improving constipation and supporting gut microbiota.<br>- Importance of adequate hydration and strategies to increase daily fluid intake.
Levodopa and Nutrients	- How levodopa absorption interacts with dietary protein.<br>- Identification of protein-rich foods and recommended intake.<br>- Meal-time strategies to minimize levodopa–protein interaction.

Source: Author

During the same visit, participants will receive a personalized diet plan, followed by a 30-minute explanation on how to implement it. Based on the baseline dietary assessment, the individualized diet plan will be previously designed according to each participant’s food preferences and cultural eating patterns within the Brazilian context. The plan will outline a full day of meals (breakfast, lunch, afternoon snack, dinner, and supper) with portion sizes provided in grams and standard household measurements. Each meal will include food options.

Diet plans will be prescribed by the study dietitian, considering age, sex, weight, BMI, physical activity level, sarcopenia risk, and stage of disease progression. The nutrient

distribution will follow these principles: (1) energy intake estimated using the Estimated Energy Requirement (EER) [38], (2) protein intake is set to 0.8 g/kg/day and Protein Redistribution Diet (PRD) prioritized when appropriate, (3) carbohydrates composing 45–65% of total energy intake, (4) lipids composing 20–35% of total energy intake, and (5) fiber intake set at a minimum of 14 g/1000 kcal.

Food selection will emphasize items aligned with the counseling recommendations, prioritizing minimally processed and *in natura* foods, while restricting ultra-processed products and high-sugar foods. PRD will be recommended for participants using levodopa, when feasible and safe. PRD is defined as limiting protein-rich foods at breakfast and lunch, with higher protein offerings at dinner and supper to meet total daily protein requirements. This strategy has been associated with improvements in motor fluctuations and lower levodopa requirements [39]. Fiber intake from fruits, vegetables, and whole-grain cereals will be emphasized, alongside adequate hydration. Fluid intake will follow a target of 30 mL/kg/day, adjusted to age and clinical characteristics of the PD population.

Nutritional counseling, dietary goals and diet plan implementation will be reinforced every 15 days through 30-minute phone calls with the study dietitian, totaling five sessions. All four counseling topics will be reviewed during each call, and participants will have the opportunity to discuss any difficulties or questions related to the diet plan.

At midpoint (D45), 24-hour dietary recalls and diet quality will be analyzed. Individual goals, nutrient composition, and overall diet quality will be reviewed. Adherence to the diet plan will be defined as meeting at least 75% of the nutrient goals. If participants withdraw or fail to fully comply, primary outcome data will still be collected whenever possible to support an intention-to-treat analysis.

Control Group

The control group will continue to receive usual care from their personal neurologist, consisting of standard clinical practice for constipation management. This may include lifestyle advice (adequate hydration, dietary fiber intake, and physical activity) as well as over-the-counter laxatives, when clinically indicated. They will not receive structured nutritional counseling during the trial period. Participants will receive follow-up calls every 15 days to address constipation, maintain engagement in the study until the final evaluation, and ensure consistency with the interaction frequency of the intervention group. At the end of the study, if proven beneficial, the control group will be offered the same nutritional counseling provided to the intervention group (wait-list design).

Statistical analysis

Participants will be identified by numeric codes to ensure confidentiality. All collected data will be collected and managed using REDCap electronic data capture tools hosted at *Universidade Federal do Rio Grande do Sul* [40], accessible only to the responsible investigators. After data entry, the dataset will be exported to SPSS version 18.0 for statistical analysis.

Descriptive statistics will be presented according to variable type: mean and standard deviation (or median and interquartile range) for continuous variables, and absolute frequencies and percentages for categorical variables.

Intent-to-treat (ITT) analyses will be performed for all outcomes, including all randomized participants. The last available data from withdrawn participants will be carried forward to D90 for ITT analyses. Change in weekly bowel movements (D90–baseline) will be compared between groups using Student’s t-test. Secondary outcomes will be analyzed using generalized linear mixed models with group, time, and their interaction as fixed effects, and participants as a random effect to account for repeated measures. Age, sex, disease duration,

and levodopa equivalent daily dose will be included as covariates when relevant. Data will be reported as mean ± SEM, and a two-sided $p < 0.05$ will denote statistical significance.

For gut microbiota analysis, alpha-diversity will be calculated using the Shannon index, and statistical significance will be assessed using linear mixed-effects models. beta diversity will be examined by Principal Component Analysis (PCA), Principal Coordinates Analysis (PCoA), and Non-metric Multidimensional Scaling (NMDS), based on Aitchison and Jaccard distances. Statistical significance and explained variance will be assessed using Permutational Multivariate Analysis of Variance (PERMANOVA) (Anderson, 2014) and linear mixed-effects models.

Differential abundance analysis will be performed using MaAsLin2 (Multivariable Association with Linear Models 2) (Mallick et al., 2021). Multiple testing corrections will be applied using the Benjamini–Hochberg method (Benjamini & Hochberg, 1995) to control the false discovery rate (FDR), and a mixed directional FDR (mdFDR) approach with Holm's procedure (Holm, 1979) to control the familywise error rate. Statistical significance will be set at $\alpha = 0.05$.

For continuous variables with missing values, multiple imputation will be applied to estimate plausible values based on available data, thereby reducing bias and preserving statistical power.

Ethical Considerations

This project has been approved by the Research Ethics Committee of Hospital de Clínicas de Porto Alegre under protocol number 2025-0374 and registered in ClinicalTrials.gov under NCT07213856. This is protocol version 1.0, dated January 2026. Researchers declare compliance with the Declaration of Helsinki and the General Data Protection Law (*Lei Nº 13.709*, August 14, 2018) regarding the handling of personal and sensitive data.

De-identified individual participant data and statistical code will be made available upon reasonable request after results publication, subject to ethical approval and data protection regulations. All potential risks and benefits are described in the ICF, and participants will provide specific consent for stool sample collection and microbiota analysis.

Potential adverse events related to dietary changes (e.g., bloating, abdominal discomfort, unintended weight loss) will be monitored systematically during follow-up calls and visits. Given the low-risk behavioral nature of the intervention, no Data Monitoring Committee was established. Trial conduct will be monitored by the principal investigators through regular team meetings.

All data will be anonymized, especially during statistical analysis. After the trial, if the intervention demonstrates benefit, participants in the control group will be offered the same nutritional counseling as a matter of ethical fairness. Additionally, all participants will be able to request feedback on their individual questionnaire and assessment results.

The funder had no role in the study design, data collection, analysis, interpretation of data, or decision to submit the manuscript for publication. The trial is coordinated by the principal investigators, and no independent steering committee or endpoint adjudication committee was established due to the low-risk nature of the intervention.

Discussion

This study is the first randomized controlled trial identified to date to evaluate the effect of a dietitian-led nutritional intervention for managing constipation in patients with PD. Evidence for nutritional interventions on PD constipation and gut health is scarce and generally focuses on a specific dietary pattern or a supplement [13,41–43]. Considering the heterogeneity of the literature on this topic, recommendations have been pointing to a minimally processed plant-based diet with dietary counseling tailored to personal preference, cultural context, and socioeconomic realities [44].

By testing a structured, individualized nutritional intervention, this trial directly addresses this gap in evidence-based management of constipation in PD. Dietitian-led interventions have demonstrated superior improvements compared to usual care or dietary advice provided by other health professionals in the management of prediabetes [18], diabetes type 2 [17] and hypertension [19]. In PD, only 11–15% of patients have access to dietetic care, which calls for further research in this area [16]. The trial is also designed to investigate a range of secondary outcomes, including changes in gut microbiota composition, dietary intake and quality, body composition, and markers of sarcopenia. Investigating these outcomes will provide more consistency to the importance and influence of diet in PD management.

If successful, this trial will contribute robust evidence to support the integration of specialized nutritional counseling into the standard of care for PD. Such integration has the potential not only to improve quality of life but also to advance precision nutrition approaches that consider the interplay between microbiota, diet, and disease progression. The findings could strengthen clinical guidelines and reinforce the inclusion of nutrition as a key component of multidisciplinary care in PD.

This trial has some limitations. First, restricting eligibility to patients with a maximum H&Y stage of 2 limits generalizability to individuals with more advanced PD. However, this criterion is necessary to ensure adherence and completion of assessments, as more severe disease may compromise participation. Although constipation is also common in later stages, nutritional management must be individualized by disease severity, which already limits broad generalization. Second, as with any behavioral intervention, complete uniformity of counseling sessions is difficult to guarantee. To address this, the diet manual and individualized diet plan were standardized to enhance fidelity. Home stool sample collection may also affect microbiota analysis due to variations in storage and transport, but requesting in-center collection would be impractical for constipated patients; therefore, clear instructions will be reinforced during visits

and follow-ups. Despite these limitations, the protocol was designed to maximize internal validity through rigorous standardization and fidelity monitoring.

Acknowledgments

The authors thank the FONAD research group from *Hospital de Clínicas de Porto Alegre* for their scientific support in the development of this project and future assistance with data collection. We thank the Graduate Program in Medical Sciences and the Universidade Federal do Rio Grande do Sul (UFRGS) for their institutional support.

References

[1] Bloem BR, Okun MS, Klein C. Parkinson's disease. The Lancet 2021;397:2284–303.

[2] Dorsey E, Elbaz A, Nichols E, Abd-Allah F, Abdelalim A, Adsuar J, et al. GBD 2016 Parkinson's Disease Collaborators. Global, regional, and national burden of Parkinson's disease, 1990-2016: a systematic analysis for the Global Burden of Disease Study 2016. Lancet Neurol 2018;17:939–53.

[3] Goldman JG, Volpe D, Ellis TD, Hirsch MA, Johnson J, Wood J, et al. Delivering Multidisciplinary Rehabilitation Care in Parkinson's Disease: An International Consensus Statement. J Parkinsons Dis 2024;14:135–66. https://doi.org/10.3233/JPD-230117.

[4] Pfeiffer RF. Gastrointestinal dysfunction in Parkinson's disease. Parkinsonism & Related Disorders 2011;17:10–5.

[5] Arora C, Kamble N, Holla VV, Mahale R, Mythirayee S, Yadav R, et al. The prevalence of gastrointestinal dysfunction and its impact on quality of life in Parkinson's disease. J Neural Transm (Vienna) 2025. https://doi.org/10.1007/s00702-025-03038-3.

[6] Tan AH, Chuah KH, Beh YY, Schee JP, Mahadeva S, Lim S-Y. Gastrointestinal dysfunction in Parkinson's disease: neuro-gastroenterology perspectives on a multifaceted problem. Journal of Movement Disorders 2023;16:138.

[7] Postuma RB, Gagnon J, Pelletier A, Montplaisir J. Prodromal autonomic symptoms and signs in Parkinson's disease and dementia with Lewy bodies. Movement Disorders 2013;28:597–604.

[8] Salim S, Ahmad F, Banu A, Mohammad F. Gut microbiome and Parkinson's disease: Perspective on pathogenesis and treatment. Journal of Advanced Research 2023;50:83–105.

[9] Pedrosa Carrasco AJ, Timmermann L, Pedrosa DJ. Management of constipation in patients with Parkinson's disease. NPJ Parkinson's Disease 2018;4:6.

[10] Chang L, Chey WD, Imdad A, Almario CV, Bharucha AE, Diem S, et al. American Gastroenterological Association-American College of Gastroenterology clinical practice guideline: pharmacological management of chronic idiopathic constipation. Gastroenterology 2023;164:1086–106.

[11] Quigley EMM. Constipation in Parkinson's Disease. Semin Neurol 2023;43:562–71. https://doi.org/10.1055/s-0043-1771457.

[12] Barber TM, Kabisch S, Pfeiffer AFH, Weickert MO. The Effects of the Mediterranean Diet on Health and Gut Microbiota. Nutrients 2023;15:2150. https://doi.org/10.3390/nu15092150.

[13] Rusch C, Beke M, Nieves Jr C, Mai V, Stiep T, Tholanikunnel T, et al. Promotion of a Mediterranean Diet Alters Constipation Symptoms and Fecal Calprotectin in People with Parkinson's Disease: A Randomized Controlled Trial. Nutrients 2024;16:2946.

[14] Ashraf W, Pfeiffer RF, Park F, Lof J, Quigley EM. Constipation in Parkinson's disease: objective assessment and response to psyllium. Movement Disorders 1997;12:946–51.

[15] Trinh J, de Vries NM, Chan P, Dekker MC, Helmich RC, Bloem BR. The role of lifestyle interventions in symptom management and disease modification in Parkinson's disease. The Lancet Neurology 2025.

[16] Flanagan R, Rusch C, Lithander FE, Subramanian I. The missing piece of the puzzle–The key role of the dietitian in the management of Parkinson's disease. Parkinsonism & Related Disorders 2024:106021.

[17] Møller G, Andersen HK, Snorgaard O. A systematic review and meta-analysis of nutrition therapy compared with dietary advice in patients with type 2 diabetes†. The American Journal of Clinical Nutrition 2017;106:1394–400. https://doi.org/10.3945/ajcn.116.139626.

[18] Dudzik JM, Senkus KE, Evert AB, Raynor HA, Rozga M, Handu D, et al. The effectiveness of medical nutrition therapy provided by a dietitian in adults with prediabetes: a systematic review and meta-analysis. The American Journal of Clinical Nutrition 2023;118:892–910.

[19] Senkus KE, Dudzik JM, Lennon SL, DellaValle DM, Moloney LM, Handu D, et al. Medical nutrition therapy provided by a dietitian improves outcomes in adults with

prehypertension or hypertension: a systematic review and meta-analysis. The American Journal of Clinical Nutrition 2024;119:1417–42. https://doi.org/10.1016/j.ajcnut.2024.04.012.

[20] Postuma RB, Berg D, Stern M, Poewe W, Olanow CW, Oertel W, et al. MDS clinical diagnostic criteria for Parkinson’s disease. Movement Disorders 2015;30:1591–601.

[21] Drossman DA, Hasler WL. Rome IV—functional GI disorders: disorders of gut-brain interaction. Gastroenterology 2016;150:1257–61.

[22] Borges RB, Mancuso ACB, Camey SA, Leotti VB, Hirakata VN, Azambuja GS, et al. Power and Sample Size for Health Researchers: uma ferramenta para cálculo de tamanho amostral e poder do teste voltado a pesquisadores da área da saúde. Clinical and Biomedical Research 2020;40.

[23] Soares T, Vale TC, Guedes LC, Maciel RO, Antunes AP, Camargos S, et al. Validation of the Portuguese MDS-UPDRS: Challenges to Obtain a Scale Applicable to Different Linguistic Cultures. Movement Disorders Clinical Practice 2025;12:34–42. https://doi.org/10.1002/mdc3.14236.

[24] Tumas V, Borges V, Ballalai-Ferraz H, Zabetian CP, Mata IF, Brito M, et al. Some aspects of the validity of the Montreal Cognitive Assessment (MoCA) for evaluating cognitive impairment in Brazilian patients with Parkinson’s disease. Dementia & Neuropsychologia 2016;10:333–8.

[25] Ayres A, Ghisi M, Rieder CR de M, Manor Y, Olchik MR. Tradução e adaptação cultural do swallowing disturbance questionnaire para o português-brasileiro. Revista CEFAC 2016;18:828–34.

[26] Matsudo S, Araújo T, Matsudo V, Andrade D, Andrade E, Oliveira LC, et al. QUESTIONÁRIO INTERNACIONAL DE ATIVIDADE FÍSICA (IPAQ): ESTUDO DE VALIDADE E REPRODUTIBILIDADE NO BRASIL. Revista Brasileira de Atividade Física & Saúde 2001;6:5–18. https://doi.org/10.12820/rbafs.v.6n2p5-18.

[27] Taniguchi TM, ABREU GE de, Portugal MM, BARROSO JUNIOR U. Cross-cultural adaptation and validation of the Constipation Scoring System for the Brazilian population. Arquivos de Gastroenterologia 2022;59:3–8.

[28] Henn RL, Fuchs SC, Moreira LB, Fuchs FD. Development and validation of a food frequency questionnaire (FFQ-Porto Alegre) for adolescent, adult and elderly populations from Southern Brazil. Cadernos de Saude Publica 2010;26:2068–79.

[29] Louzada ML da C, Souza TN, Frade E, Gabe KT, Patricio GA. QuestNova: inovação na avaliação do consumo alimentar segundo o processamento industrial. Revista de Saúde Pública 2024;58:38.

[30] Costa C dos S, Santos FS dos, Gabe KT, Steele EM, Marrocos-Leite FH, Khandpur N, et al. Description and performance of two diet quality scores based on the Nova classification. Revista de Saude Publica 2024;58:47.

[31] Brasil. Orientações para a coleta e análise de dados antropométricos em serviços de saúde: Norma Técnica do Sistema de Vigilância Alimentar e Nutricional-SISVAN 2011.

[32] World Health Organization (WHO). Obesity: preventing and managing the global epidemic: report of a WHO consultation 2000.

[33] Rubenstein LZ, Harker JO, Salvà A, Guigoz Y, Vellas B. Screening for undernutrition in geriatric practice: developing the short-form mini-nutritional assessment (MNA-SF). The Journals of Gerontology Series A: Biological Sciences and Medical Sciences 2001;56:M366–72.

[34] Cruz-Jentoft AJ, Bahat G, Bauer J, Boirie Y, Bruyère O, Cederholm T, et al. Sarcopenia: revised European consensus on definition and diagnosis. Age and Ageing 2019;48:16–31.

[35] Burgos R, Bretón I, Cereda E, Desport JC, Dziewas R, Genton L, et al. ESPEN guideline clinical nutrition in neurology. Clinical Nutrition 2018;37:354–96.

[36] Alves JTM, Gonçalves TJM, Ribeiro PC, de Albuquerque CL, Alvite M de FL, de Assis T, et al. Diretriz BRASPEN de terapia nutricional no paciente com doenças neurodegenerativas. Braspen Journal 2023;37:2–34.

[37] Brasil M da S Secretaria de Atenção à Saúde, Departamento de Atenção Básica. Guia alimentar para a população brasileira. 2nd ed. Brasília: Ministério da Saúde; 2014.

[38] Cominetti C, Cozzolino SMF. Recomendações de nutrientes. 3rd ed. São Paulo: International Life Sciences Institute do Brasil – ILSI Brasil; 2023.

[39] Barichella M, Cereda E, Cassani E, Pinelli G, Iorio L, Ferri V, et al. Dietary habits and neurological features of Parkinson’s disease patients: implications for practice. Clinical Nutrition 2017;36:1054–61.

[40] Harris PA, Taylor R, Minor BL, Elliott V, Fernandez M, O’Neal L, et al. The REDCap consortium: building an international community of software platform partners. Journal of Biomedical Informatics 2019;95:103208.

[41] Barichella M, Pacchetti C, Bolliri C, Cassani E, Iorio L, Pusani C, et al. Probiotics and prebiotic fiber for constipation associated with Parkinson disease: an RCT. Neurology 2016;87:1274–80.

[42] Ibrahim A, Ali RAR, Manaf MRA, Ahmad N, Tajurruddin FW, Qin WZ, et al. Multi-strain probiotics (Hexbio) containing MCP BCMC strains improved constipation and gut motility in Parkinson's disease: A randomised controlled trial. Plos One 2020;15:e0244680.
[43] Tan AH, Lim S-Y, Chong KK, A Manap MAA, Hor JW, Lim JL, et al. Probiotics for constipation in Parkinson disease: a randomized placebo-controlled study. Neurology 2021;96:e772–82.
[44] Subramanian I, Ricciardi L, Schrag A, Appel-Cresswell S, Kola S, Domingos JM, et al. A Holistic Wellness Prescription for Parkinson's Disease: Evidence-Based Perspectives and Unmet Needs. Movement Disorders Clinical Practice n.d.

Supporting Information

S1 Appendix. SPIRIT checklist.

S2 Appendix. Dietary Intervention Manual.

S3 Appendix. Study protocol approved by the ethics committee (Original version)

S4 Appendix. Study protocol approved by the ethics committee (English translation)

Figure 1 Click here to access/download;Figure;Fig1.tiff

	NUTRI-GUT-PD TRIAL							
	**Baseline**	**Post-randomization**						**Close-out**
**TIMEPOINT**	***D-7***	**D0**	***D15*** (phone call)	***D30*** (phone call)	***D45*** (phone call)	***D60*** (phone call)	***D75*** (phone call)	***D90***
**ENROLLMENT:**								
**Eligibility screen**	X							
**Informed consent**	X							
**Randomization** [a]	X							
**INTERVENTION/ COMPARATOR:**								
Intervention Group		X	X	X	X	X	X	X
Control Group		X	X	X	X	X	X	X
**ASSESSMENTS:**								
Demographics	X							
Neurological Evaluation	X							X
CSS	X				X			X
Stool diary		X			X			X
Stool sample collection [b]		X						X
FFQ	X							
24-hour dietary recalls [c]		X			X			X
Screener Nova		X			X			X
Anthropometry	X							X
BIA		X						X
Nutritional Risk Screening		X						X
Sarcopenia Assessment	X							X

Figure 2

Click here to access/download;Figure;Fig2.tiff

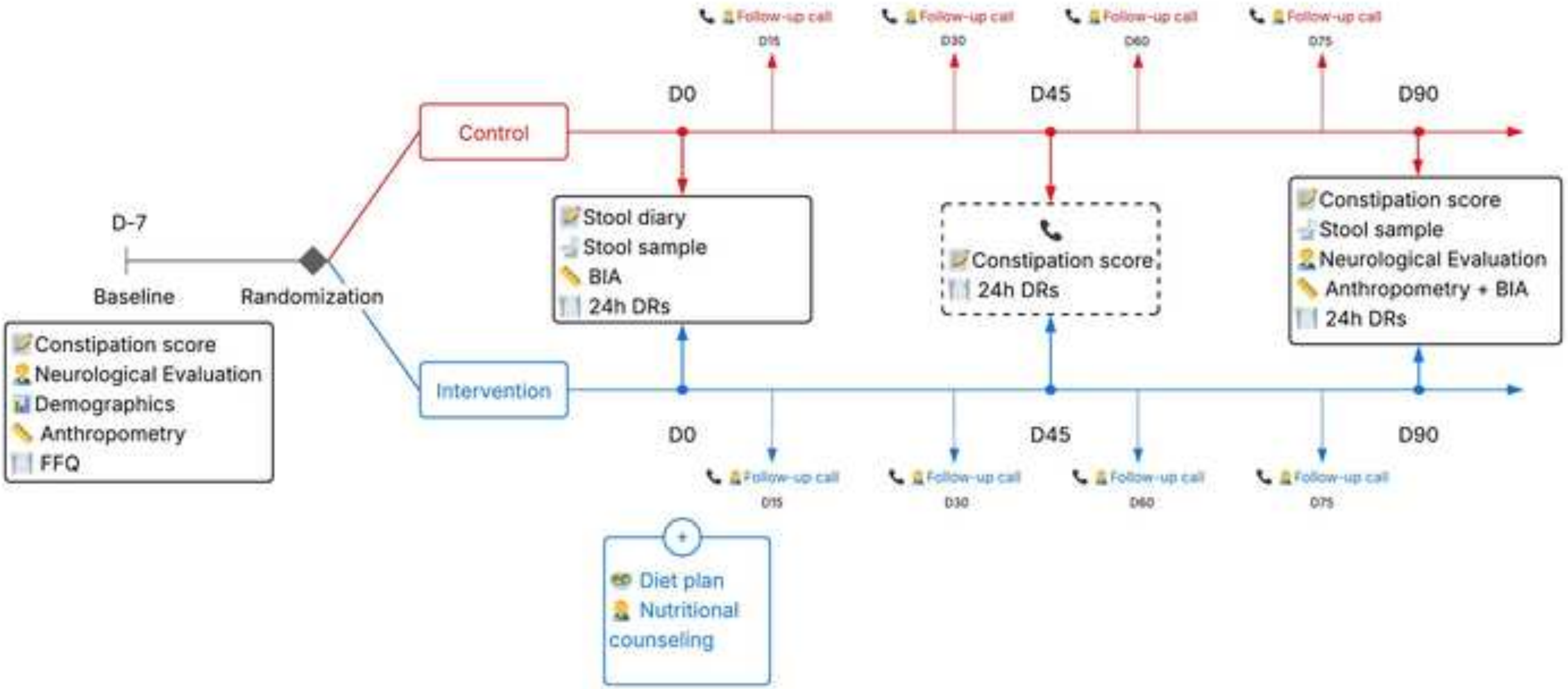

Supporting Information 1

Click here to access/download
Supporting Information
S1_Appendix.docx.pdf

Supporting Information 2

Click here to access/download
Supporting Information
S2_Appendix.pdf

Supporting Information 3

Click here to access/download
Supporting Information
S3_Appendix.pdf

Supporting Information 4

Click here to access/download
Supporting Information
S4_Appendix.pdf
