# Supplementary material for: Dietitian-led intervention to manage constipation in Parkinson’s disease: Study protocol for a parallel-group randomized controlled trial (NUTRI-GUT-PD)
Source: PLoS One. 2026 Jul 27;21(7):e0354608. doi: 10.1371/journal.pone.0354608 (PMC13405298; doi:10.1371/journal.pone.0354608)
Supplement: Attachment — Submitted filename: PONE-D-26-05353_reviewer.docx [file pone.0354608.s004.docx]

**Reviewer Comments – Major Revision**

**General Comments:**
The manuscript describes a study protocol with important potential contributions to understanding the role of gut microbiota and nutrition in Parkinson’s disease (PD)-related constipation. While the study is generally well designed, several issues need to be addressed to improve clarity, methodological rigor, and reporting consistency before the manuscript can be considered for publication. I recommend **major revision**.

**Specific Comments:**

1. **Blinding Procedures:**
   The blinding procedures are described; however, it is not explicitly stated whether the neurologist conducting the study is one of the outcome assessors who are blinded. This should be clarified to ensure methodological transparency.
2. **Abstract:**
   On line 29, replace “study” with “study protocol” to accurately reflect the manuscript type.
3. **Introduction:**

- The introduction should be strengthened with more compelling statements regarding gut microbiota and constipation in Parkinson’s disease.
- Literature on PD-gut microbiota should be better contextualized, particularly human microbiome studies, to justify the planned 16S rRNA analysis.
- Line 74 references study 13, which demonstrated the effectiveness of the Mediterranean diet; the sentence should be revised to accurately reflect the study findings.
- Lines 75–76 reference study 14, which observed no changes in colonic structure; this point should also be addressed.
- The final paragraph should clearly state the primary objective and, if applicable, explicitly define primary and secondary hypotheses.

4. **Eligibility Criteria:**

- The protocol should clarify how continuous or intermittent use of osmotic laxatives (e.g., magnesium oxide) will be handled in terms of eligibility, follow-up, and statistical analysis.
- Since the intervention relies on individualized food-based counseling, the authors should clarify whether participants requiring texture-modified diets, feeding assistance, or having relevant chewing limitations are eligible, and how adherence will be ensured in such cases.

5. **Data Collection:**

- Physical activity records should be monitored at the midpoint as well, or the analysis should explicitly consider them as potential confounders.
- The term “Three 24-hour Dietary Recalls” is insufficiently precise. It should be clarified whether dietary intake will be recorded for a single day per session or over three days. Consistent terminology should be used throughout the manuscript (e.g., “A 3-day diet diary [two weekdays and one weekend day] was used to assess dietary intake” or “24-hour dietary recall was used to assess dietary intake”). These records can then be used to calculate a diet quality index.
- Using both food frequency assessment and the Screener-Nova is unnecessary. Evaluating food frequency at baseline and study end may be more appropriate.
- For anthropometric measures, calf circumference may not be required since it is already included in the MNA short form. Similarly, arm circumference may not be necessary because BIA and muscle strength measurements are performed; hip circumference can be preferred instead.
- In Figure 1, “nutritional risk screening” can be replaced with “MNA-SF” directly for clarity.

6. **References:**
   Recent references should be included where available to support background on PD constipation, microbiota, and nutrition interventions, while retaining seminal methodological references.
