# Supplementary material for: Dietitian-led intervention to manage constipation in Parkinson’s disease: Study protocol for a parallel-group randomized controlled trial (NUTRI-GUT-PD)
Source: PLoS One. 2026 Jul 27;21(7):e0354608. doi: 10.1371/journal.pone.0354608 (PMC13405298; doi:10.1371/journal.pone.0354608)
Supplement: Attachment — Submitted filename: Response to Reviewers.docx [file pone.0354608.s007.docx]

**Response to Reviewers**

Manuscript: PONE-D-26-05353

Dear Editor and Reviewers,

We would like to express our sincere gratitude to the Editor and all three Reviewers for the thorough and thoughtful evaluation of our manuscript entitled *“Dietitian-led Intervention to Manage Constipation in Parkinson’s Disease: Study Protocol for a Parallel-Group Randomized Controlled Trial (NUTRI-GUT-PD)”*. We greatly appreciate the comments and suggestions provided during the review process. We carefully revised the manuscript, considering all recommendations, which contributed to improving the clarity, methodological rigor, and overall quality of the study protocol.

Among the main revisions, the statistical analysis section was revised and clarified, and the intervention protocol was described in greater detail to improve reproducibility and methodological transparency. In addition, all minor comments and suggestions provided by the reviewers were carefully addressed throughout the manuscript, and all changes in the revised manuscript have been highlighted.

To facilitate the review of the new version, we prepared the point-by-point response document below, specifying the location of each modification (document and page number).

We sincerely hope that the revised version satisfactorily addresses all concerns raised, and we remain available for any further clarification.

Sincerely,

**Prof. Maira Rozenfeld Olchik**

*Corresponding author on behalf of all authors*

| **POINTS RAISED DURING REVIEW** | **AUTHORS’ RESPONSE** |
| --- | --- |
| **JOURNAL REQUIREMENTS** | |
| 1. Please ensure that your manuscript meets PLOS ONE’s style requirements, including those for file naming. | We thank the editors for this reminder. The manuscript and all associated files have been revised to comply with PLOS ONE’s style requirements. Specifically, file naming has been updated in accordance with the guidelines provided at the PLOS ONE formatting templates. These adjustments are reflected throughout the revised manuscript. |
| 2. Your ethics statement should only appear in the Methods section of your manuscript. If your ethics statement is written in any section besides the Methods, please delete it from any other section. | We appreciate the editorial guidance. The ethics statement has been removed from all sections other than the Methods section, where it now appears exclusively under the subheading “Ethical Considerations” (Manuscript, page 20). |
| 3. We note that the Figures in S2_Appendix.pdf in your submission contain copyrighted images. We require you to either (1) present written permission from the copyright holder to publish these figures specifically under the CC BY 4.0 license, or (2) remove the figures from your submission. | We thank the editors for bringing this to our attention. As obtaining CC BY 4.0 permission from all original copyright holders within the revision timeline was not feasible, we opted to remove all figures from the S2_Appendix. The supplementary material has been revised accordingly, and the updated S2_Appendix no longer contains any copyrighted figures. |
| 4. Thank you for providing your underlying data as Supporting Information. We note that the data set contains text or data that is not in English. Please upload an English-language version of your data set. | We appreciate this requirement. The summary table in S4_Appendix has been translated into English. Regarding S3_Appendix (the original trial protocol approved by the ethics committee), it is a regulatory document in Brazilian Portuguese; an English translation has been provided as S4_Appendix. All other documents already include English-language versions, as indicated in each file. |
| **REVIEWER #1** | |
| 1. Blinding Procedures: It is not explicitly stated whether the neurologist conducting the study is one of the outcome assessors who are blinded. This should be clarified to ensure methodological transparency. | We are grateful for this observation. The blinding procedures have been clarified in the revised manuscript under the “Randomization” subheading (Manuscript, page 6-7). Specifically, we now state that the neurologist involved in outcome assessment is blinded to group allocation and is not the same professional responsible for randomization or intervention delivery. |
| 2. Abstract: On line 29, replace “study” with “study protocol” to accurately reflect the manuscript type. | Thank you for this careful observation. The word “protocol” has been added to the Abstract (Manuscript, page 2, line 28), which now reads “This study protocol aims to examine...” |
| 3a. Introduction: The introduction should be strengthened with more compelling statements regarding gut microbiota and constipation in Parkinson’s disease. | We thank the reviewer for this suggestion. The Introduction has been substantially revised to include more compelling and up-to-date statements on the role of gut microbiota in constipation in PD. A new dedicated paragraph summarizing recent findings on gut dysbiosis and its mechanistic relationship with PD pathology has been added (Manuscript, pages 3-4). |
| 3b. Literature on PD-gut microbiota should be better contextualized, particularly human microbiome studies, to justify the planned 16S rRNA analysis. | We appreciate this important point. A new paragraph has been included in the Introduction specifically contextualizing human gut microbiome studies in PD, including those using 16S rRNA sequencing, to provide a stronger scientific rationale for the planned microbiota analysis (Manuscript, page 3-4). |
| 3c. Line 74 references study 13, which demonstrated the effectiveness of the Mediterranean diet; the sentence should be revised to accurately reflect the study findings. | We agree and have revised the sentence. Reference 18 (the MediDiet study) concluded that both the Mediterranean diet and standard care reduced constipation symptoms, with the Mediterranean diet additionally providing benefits of increased dietary fiber intake and less intestinal inflammation. The Introduction has been updated to accurately reflect these findings (Manuscript, page 4, lines 86-91). |
| 3d. Lines 75–76 reference study 14, which observed no changes in colonic structure; this point should also be addressed. | Thank you for this observation. The sentence referencing study 19 has been revised to more accurately describe its findings, noting that psyllium fiber supplementation improved constipation symptoms but did not alter colonic structure, and that the evidence was limited to a small sample (Manuscript, page 4, lines 91-93). |
| 3e. The final paragraph should clearly state the primary objective and, if applicable, explicitly define primary and secondary hypotheses. | We agree that this was insufficiently stated. The final paragraph of the Introduction now explicitly presents the primary objective of the trial (to investigate whether individualized nutritional counseling, delivered by a dietitian, is more effective than usual care in increasing the frequency of bowel movements in patients with PD) and includes a clear statement of the hypotheses (Manuscript, page 5, lines 103-107). |
| 4a. Eligibility Criteria: The protocol should clarify how continuous or intermittent use of osmotic laxatives (e.g., magnesium oxide) will be handled in terms of eligibility, follow-up, and statistical analysis. | We appreciate this methodological concern. The revised manuscript clarifies that continuous use of any laxative remains an exclusion criterion (item 10 of the eligibility criteria). Intermittent laxative use, however, has been retained as permissible to avoid selection bias and ensure adequate representation of the target population, since many constipated PD patients use laxatives occasionally. Laxative use is recorded in the stool diary throughout the trial, and it will be included in the generalized linear mixed models (GLMM) analysis as a time-varying covariate. As participants are matched in pairs, we expect intermittent use to be similarly distributed between groups (Manuscript, page 6; Trial Protocol, updated accordingly). |
| 4b. Since the intervention relies on individualized food-based counseling, the authors should clarify whether participants requiring texture-modified diets, feeding assistance, or having relevant chewing limitations are eligible, and how adherence will be ensured in such cases. | We thank the reviewer for raising this practical concern. Exclusion criterion 11 has been rewritten in the revised manuscript to explicitly state that participants who require diets in consistencies other than FOIS Level 7 (total oral diet with no restrictions) will be excluded (Manuscript, page 6, exclusion criterion 11). |
| 5a. Data Collection: Physical activity records should be monitored at the midpoint as well, or the analysis should explicitly consider them as potential confounders. | We agree with this recommendation. The IPAQ-SF has been added to the D45 assessment as well. This allows physical activity to be monitored at baseline, midpoint, and endpoint, and will be included as a covariate in the GLMM analyses (Manuscript, page 10; Figure 1, updated). |
| 5b. The term “Three 24-hour Dietary Recalls” is insufficiently precise. It should be clarified whether dietary intake will be recorded for a single day per session or over three days. Consistent terminology should be used throughout the manuscript (e.g., “A 3-day diet diary [two weekdays and one weekend day] was used to assess dietary intake” or “24-hour dietary recall was used to assess dietary intake”). These records can then be used to calculate a diet quality index. | We thank the reviewer for this suggestion. The term “Three 24-hour dietary recalls” has been retained and is now clearly defined in the Dietary Assessment subsection as representing three separate 24-hour recall assessments conducted by the dietitian on different days (two weekdays and one weekend day) at each measurement timepoint, consistent with current evidence for reliable usual nutrient intake estimation. Terminology has been standardized throughout the manuscript and supporting protocol (Manuscript, pages 12-13). |
| 5c. Using both food frequency assessment and the Screener-Nova is unnecessary. Evaluating food frequency at baseline and study end may be more appropriate. | We thank the reviewer for this suggestion. After careful consideration, we have decided to remove the food frequency questionnaire (FFQ) and retain only the three 24-hour dietary recalls and the Screener-Nova. The FFQ has a higher underreporting rate (approximately 29–34%) compared to 24-hour recalls (15–17%) (Park Y et al, 2018), requires considerably more time to administer, and is not appropriate for reassessment after only three months given its one-year reference period. Eliminating the FFQ also reduces the cognitive burden on this neurological population. These changes are reflected in the revised manuscript and Study Protocol (Manuscript, page 13; Trial Protocol, updated). |
| 5d. For anthropometric measures, calf circumference may not be required since it is already included in the MNA short form. Similarly, arm circumference may not be necessary. Hip circumference can be preferred instead. | We appreciate this practical suggestion. Regarding calf circumference: it will be retained as a standalone anthropometric measure since participants will already be in lightweight clothing for the assessment and it will later be incorporated into the MNA-SF scoring. Arm circumference has also been retained because it is a rapid, easy-to-collect measure that can serve as a proxy for skeletal muscle mass; cutoff points have been added. Hip circumference has been added to the anthropometric assessment protocol, as recommended (Manuscript, page 13; Trial Protocol, updated). |
| 5e. In Figure 1, “nutritional risk screening” can be replaced with “MNA-SF” directly for clarity. | Thank you for this suggestion. The label in Figure 1 has been updated to “MNA-SF” (Figure 1, updated). |
| 6. References: Recent references should be included where available to support background on PD constipation, microbiota, and nutrition interventions, while retaining seminal methodological references. | We agree. Recent references have been incorporated throughout the revised manuscript, particularly in the Introduction and Methods sections, to support the expanded discussion of gut microbiota, constipation in PD, and nutrition interventions. |
| **REVIEWER #2** | |
| • Defining the primary outcome with a single, clear measurement. | We thank Reviewer #2 for this important suggestion. The primary outcome has been redefined and presented as a single, clear measure: the change in weekly bowel movement frequency from baseline to D90, as recorded in the stool diary. This is now stated unambiguously in both the Abstract and the Methods section under “Outcomes” (Manuscript, pages 2 and 7). |
| • Updating the statistical analysis plan with an appropriate model for the census data. | We appreciate this methodological comment. The statistical analysis plan has been updated: GLMM will now be used as the primary analytical approach for all outcomes, including the primary outcome (weekly bowel movement frequency), replacing the previously described t-test. GLMM appropriately accounts for the repeated measures nature of the data and the correlation structure inherent in the paired design. The updated analysis plan is described in the revised Methods section under “Statistical Analysis” (Manuscript, page 19) and has been reflected and approved in the trial protocol by the ethics committee (S3/S4 Appendix). |
| • Presenting the sample size calculation along with its sources. | We are grateful for this comment. The sample size calculation has been expanded to include the specific parameters used (effect size, standard deviation, power, significance level) along with their sources, which are now fully cited. This information is now presented transparently in the “Sample Size” subsection (Manuscript, page 7). |
| • Standardizing and defining the content of the control group. | We agree that greater clarity was needed. The control group (“usual care”) has been more precisely defined in the revised manuscript. In addition, the outline of the bi-weekly follow-up phone calls for the control group has been included in S2_Appendix, ensuring the comparator condition is fully standardized and reproducible. The text in the Methods section has also been revised accordingly (Manuscript, page 18; S2_Appendix). |
| • Clarifying the number of visits consistent with the SPIRIT scheme. | Thank you for this comment. The number and timing of in-person visits and remote contacts have been clarified throughout the revised manuscript to ensure full consistency with the SPIRIT schedule of assessments presented in Figure 1. The trial includes three in-person visits (baseline, D0, and D90) and five bi-weekly phone contacts (D15, D30, D45, D60, D75), with assessments at D45. This is now clearly described in the Methods section (Manuscript, page 8) and fully reflected in Figure 1. |
| • Discussing the risk of bias due to the absence of participant blindness. | We appreciate this suggestion. A discussion of the risk of bias introduced by the impossibility of blinding participants to group allocation (given the nature of the dietary intervention) has been added to the Limitations paragraph in the Discussion section. We acknowledge that performance bias is a potential concern and describe how it has been mitigated through standardized protocols and objective outcome measures (Manuscript, page 22). |
| • Updating the missing data management with modern standards and addressing the deficiencies noted in the reviewer file. | We thank Reviewer #2 for this comment. The missing data management section has been substantially revised. With the adoption of GLMM as the primary analytical framework, missing data will be handled through likelihood-based estimation under the Missing at Random (MAR) assumption, which is a methodologically sound and modern approach. The inconsistency previously present has been resolved. Sensitivity analyses will be conducted to evaluate the robustness of results under departures from the MAR assumption. These revisions are reflected in the “Statistical Analysis” subsection (Manuscript, page 19). |
| **REVIEWER #2 - Additional Comments (*Attached File*)** | |
| 1. Revise the primary outcome statement to: ‘The primary outcome is the change in weekly bowel movement frequency from baseline to day 90, as recorded in the stool diary’. Add CSS to the secondary outcomes list. | We thank Reviewer #2 for this precise suggestion. The primary outcome statement in the Abstract section has been revised to read exactly as recommended: The primary outcome is the change in weekly bowel movement frequency from baseline to day 90, measured through the stool diary. Secondary outcomes include CSS, changes in fecal gut microbiota…” |
| 2. Reference 4 (Pfeiffer 2011) alone may be insufficient to support the claim of ‘~80% prevalence’. A more recent source could be added. | We thank the reviewer for this observation. We agree that relying on a single older reference to support the prevalence estimate warrants updating. In the revised version we have added a range to reflect the updated literature. We replaced reference 4 with a more recent Cochrane systematic review (Stewart et al., 2024) that better reflects current evidence on gastrointestinal dysfunction in central neurological diseases, including PD (Manuscript, page 3, reference 4). |
| 3. A more up-to-date resource on the gut-brain axis mechanism could be added. | We appreciate this suggestion. The revised Introduction now includes two updated references specifically addressing the gut-brain axis mechanisms relevant to PD. Hajare & Kulkarni (2025, Brain Research) and Wang et al. (2021, Brain) have been added to contextualize the neural, immune, and endocrine communication pathways between the enteric nervous system, gut microbiota, and central nervous system (Manuscript, page 3, references 8 and 9). |
| 4. If the intervention has a theoretical basis (social cognitive theory, motivational interviewing, etc.), please specify. | We thank the reviewer for this important point. The theoretical basis of the nutritional counseling intervention has been made more prominent. Social Cognitive Theory (SCT) is now stated as the theoretical framework, and its key constructs targeted by the intervention are described in detail in the Intervention Group subsection (Manuscript, page 14-15). The SCT framework also underpins the structured session scripts provided in S2 Appendix (Part B). References supporting the application of SCT to dietary behavior change have been included. |
| 5. “Samples must be stored refrigerated (up to 48h) and delivered to the research team, preferably within 1 hour of removal from refrigeration.” — A reference is needed for this storage recommendation. | We agree that this recommendation requires supporting evidence. References have been added to justify the refrigerated storage window and the 1-hour post-refrigeration delivery limit. Choo et al. (2015, Scientific Reports) and Cunningham et al. (2020, PLOS ONE) are cited to support these sample handling parameters, as both studies directly assessed the effects of time and temperature on fecal microbiome profiles and short-chain fatty acid composition (Manuscript, page 12). |
| 6. Regarding the phrase “It will take approximately one hour” in the Intervention Group section: Is the duration standardized? Providing a minimum and maximum range would be more appropriate. | We thank the reviewer for this precision-oriented comment. The phrasing has been revised to provide a standardized time range rather than a fixed estimate. The manuscript now states that the first in-person visit of the intervention “will range from 60 to 90 minutes”, and the diet plan explanation component is specified as “approximately 30 minutes”. These ranges are directly aligned with the structured session outline provided in S2 Appendix (Part B) (Manuscript, page 15) |
| 7. It is not specified how individualized diet plans can be standardized and how consistency can be ensured among dietitians. Define a protocol or monitoring mechanism. Perhaps this can be done in a diagram. | We greatly appreciate this recommendation. The consistency and standardization of individualized diet plan prescription across dietitians is now explicitly addressed in the revised manuscript and supporting material. In the manuscript, we describe that decision-making is guided by a structured algorithm presented in Fig 3, which outlines the step-by-step nutrient distribution logic. This ensures that all dietitians follow the same decision pathway while accommodating individual clinical characteristics. Additionally, S2 Appendix (Part A) details the dietitian training program (Manuscript, page 17 and Fig 3). |
| 8. Will an interview script or standard operating procedure be used? | Yes, and this is now explicitly stated in the revised manuscript. S2 Appendix (Part B) contains the full structured session protocols for both the intervention and control groups. |
| 9. Whether 90 days is sufficient for microbiota change has not been debated. Briefly mention the results obtained in similar time periods in the literature. | We thank the reviewer for raising this important point. A discussion of the 90-day timeframe in the context of microbiota change has been added to the Discussion section. We acknowledge that 90 days represents a timeframe consistent with detecting meaningful microbiota shifts: gut microbiota beta-diversity changes have been documented as rapidly as 24 hours following dietary modifications, and genus-level compositional changes have been reported after 3-month dietary interventions (David et al., 2014, Nature; Pagliai et al., 2020, European Journal of Nutrition). We also note that species-level changes may require longer intervention periods, which we acknowledge as a limitation. This contextualizes the 90-day design as biologically plausible for detecting the microbiota-level effects hypothesized by this trial (Manuscript, page 21). |
| **REVIEWER #3** | |
| 1. Authors are encouraged to include a brief description of the instruments being used as part of the neurological evaluation rather than just listing them with corresponding references. Also, there are instruments mentioned in the protocol that are not in the manuscript (ANNEX-D, BDI). The protocol and manuscript should be consistent. | We are grateful for this comment. Brief descriptions of all instruments included in the neurological evaluation (MDS-UPDRS, MoCA, SDQ-BR, and IPAQ-SF) have been added to the Methods section (Manuscript, page 10). Regarding consistency: BDI has been removed from the trial protocol as it was not included in the manuscript. This amendment was formally approved by the ethics committee, and the protocol was updated accordingly. The revised ethics-approved protocol is provided in S3_Appendix (original) and S4_Appendix (English translation). |
| 2. The primary outcome is based on a change in weekly bowel movements, as captured by the stool diary and the Constipation Scoring System (CSS). The stool diary is completed daily so weekly measures are obtained over the entire 90-day duration of the trial. The CSS is measured at 3 different time points (baseline, day 45 and day 90). However, in the statistical analysis section, authors mention the analysis of weekly bowel movements will be done using a t-test. Other outcomes (that are also collected at 2-3 time points) are analyzed with generalized linear mixed models due to the repeated measures. It is not clear why the weekly bowel movements will be analyzed with a t-test, especially when weekly data over 3 months will be available. The protocol, included as part of the supplemental material, also does not indicate the t-test as the method being used to analyze data. | We sincerely thank the reviewer for this critical observation. We fully agree that GLMM is the appropriate analytical approach for the primary outcome given the repeated weekly measures available over 90 days. The statistical analysis plan has been revised: GLMM will now be used for all outcomes, including the primary outcome of weekly bowel movement frequency. This change increases the statistical power of the analysis, appropriately accounts for within-subject correlation and time effects, and eliminates the inconsistency between the manuscript and the protocol. The protocol in S3/S4 Appendix has been updated and re-approved by the ethics committee (Manuscript, page 19). |
| 3. There is an inconsistency in the manuscript for the handling of missing data. In line 369, authors indicate they will use last-observation carried forward and later in line 389, they indicate they will use multiple imputation. Authors should clarify when each approach will be applied. | We thank the reviewer for identifying this inconsistency. The missing data management has been completely revised to present a unified and coherent approach. As GLMM is now the primary analytical model, missing data will be handled through maximum likelihood estimation under the MAR assumption, which is the recommended modern approach for such models. Sensitivity analyses will be performed to assess robustness to potential departures from the MAR assumption. The contradictory references to LOCF and multiple imputations have been removed (Manuscript, page 19). |
| Minor point 1. Line 145: BIA should be defined here (it is defined in Figure captions and in line 275), but this is the first use of the acronym. | Thank you for this careful reading. The acronym BIA (Bioelectrical Impedance Analysis) has been defined at its first use in the text, at line 176 (Manuscript, page 8). |
| Minor point 2. Line 162: I believe the year should be 2026 instead of November 2027. | Thank you for catching this error. The year has been corrected to 2026 in the revised manuscript (Manuscript, page 8, line 193). |
| Minor point 3. Lines 186–187: please make this a complete sentence. | Thank you for this observation. The sentence at lines 186–187 has been revised to form a complete, grammatically correct sentence (Manuscript, page 9). |
| Minor point 4. Line 255: authors mention a nutrient analysis software – is there a specific one being used? | Thank you for this question. We will use the DietBox software for dietary analysis, specifically selecting the two food composition tables that best represent Brazilian food culture, including regional food items. This has been specified in the revised manuscript (Manuscript, page 13, line 296-299). |
