# Supplementary material for: Dietitian-led intervention to manage constipation in Parkinson’s disease: Study protocol for a parallel-group randomized controlled trial (NUTRI-GUT-PD)
Source: PLoS One. 2026 Jul 27;21(7):e0354608. doi: 10.1371/journal.pone.0354608 (PMC13405298; doi:10.1371/journal.pone.0354608)
Supplement: Attachment — Submitted filename: Response_to_Reviewers_auresp_2.docx [file pone.0354608.s008.docx]

**Response to Reviewers**

PONE-D-26-05353R1

Dear Editor and Reviewers,

We would like to express our sincere gratitude to the Editor and Reviewers for their careful assessment of our manuscript entitled *“Dietitian-led Intervention to Manage Constipation in Parkinson’s Disease: Study Protocol for a Parallel-Group Randomized Controlled Trial (NUTRI-GUT-PD)”*.

We sincerely thank Reviewer 1 for the comments. Below, we provide our detailed point-by-point responses to each comment. We carefully revisited every question and thoroughly reviewed the manuscript to ensure that all concerns were comprehensively addressed and that the corresponding revisions were clearly presented.

We noted that several comments refer to issues raised during the initial review and addressed in our previous revision. Nevertheless, to facilitate the review process and avoid any ambiguity, we have provided additional explanations where appropriate and further refined the manuscript. These revisions have improved the clarity, transparency, and overall presentation of the manuscript. All changes have been highlighted in the revised version, and the corresponding sections are identified in our responses below.

We sincerely hope that this revised version satisfactorily addresses all remaining concerns. We appreciate your time and consideration, and remain at your disposal should any additional clarification be required.

Sincerely,

**Prof. Maira Rozenfeld Olchik**

*Corresponding author on behalf of all authors*

| **POINTS RAISED DURING REVIEW** | **AUTHORS’ RESPONSE** |
| --- | --- |
| **JOURNAL REQUIREMENTS** | |
| 1.If the reviewer comments include a recommendation to cite specific previously published works, please review and evaluate these publications to determine whether they are relevant and should be cited. There is no requirement to cite these works unless the editor has indicated otherwise. | We thank the editors for this reminder. |
| 2. Please review your reference list to ensure that it is complete and correct. If you have cited papers that have been retracted, please include the rationale for doing so in the manuscript text, or remove these references and replace them with relevant current references. Any changes to the reference list should be mentioned in the rebuttal letter that accompanies your revised manuscript. If you need to cite a retracted article, indicate the article’s retracted status in the References list and also include a citation and full reference for the retraction notice. | All modifications made during the initial revision round were fully documented in our previous response letter. To the best of our knowledge, none of the articles included in our current reference list has been retracted. |
| **REVIEWER #1** | |
| **1st Review in 05/2026:**  1. Blinding Procedures: It is not explicitly stated whether the neurologist conducting the study is one of the outcome assessors who are blinded. This should be clarified to ensure methodological transparency. | **1st Review Answer in 05/2026:**  We are grateful for this observation. The blinding procedures have been clarified in the revised manuscript under the “Randomization” subheading (Manuscript, page 6-7). Specifically, we now state that the neurologist involved in outcome assessment is blinded to group allocation and is not the same professional responsible for randomization or intervention delivery. |
| **CURRENT REVIEW**  Although the manuscript states that outcome assessors, microbiota sequencing personnel, and the statistician will be blinded to group allocation, it remains unclear whether the study neurologist performing the clinical assessments (e.g., MDS-UPDRS, MoCA, SDQ, and IPAQ) is also blinded. Since these assessments contribute to secondary outcomes, please clarify whether the neurologist is considered an outcome assessor and, if so, whether blinding will be maintained throughout the study. | **CURRENT ANSWER**  We appreciate the reviewer’s question for clarification on this critical aspect. We would like to gently reiterate that the study neurologist is indeed considered an outcome assessor and will remain strictly blinded to group allocation throughout the entire study. We have further adjusted the sentence in the "Randomization" section (page 6), for clarity: “Outcome assessors, including dietitians responsible exclusively for assessments and the neurologist performing clinical evaluations at baseline and D90, as well as microbiota sequencing professionals and the statistician, will be blinded to group allocation.” |
| **1st Review in 05/2026:**  5a. Data Collection: Physical activity records should be monitored at the midpoint as well, or the analysis should explicitly consider them as potential confounders. | **1st Review in 05/2026:**  We agree with this recommendation. The IPAQ-SF has been added to the D45 assessment as well. This allows physical activity to be monitored at baseline, midpoint, and endpoint, and will be included as a covariate in the GLMM analyses (Manuscript, page 10; Figure 1, updated). |
| **CURRENT REVIEW**  The clarification regarding dietary assessments is appreciated. However, physical activity remains a potential confounder in constipation outcomes. The IPAQ-SF appears to be administered only at baseline and D90. Please either justify why physical activity is not assessed at the midpoint (D45) or explicitly indicate how changes in physical activity during the intervention period will be accounted for in the statistical analyses. | **CURRENT ANSWER**  We entirely agree with the reviewer that physical activity is a crucial confounding factor in constipation outcomes.  We would like to clarify that, following the reviewer's excellent recommendation in the previous round, we had already incorporated the IPAQ-SF into the midpoint (D45) assessment (page 10): “This assessment will be repeated at mid-point (D45) through phone contact by the study dietitian.” Furthermore, Figure 1 was previously updated to visually reflect the inclusion of the IPAQ-SF at D45. We hope this reassures the reviewer that physical activity is being rigorously monitored and statistically controlled at all three time points (Baseline, D45, and D90). |
| **1st Review in 05/2026:**  4a. Eligibility Criteria: The protocol should clarify how continuous or intermittent use of osmotic laxatives (e.g., magnesium oxide) will be handled in terms of eligibility, follow-up, and statistical analysis. | **1st Review in 05/2026:**  We appreciate this methodological concern. The revised manuscript clarifies that continuous use of any laxative remains an exclusion criterion (item 10 of the eligibility criteria). Intermittent laxative use, however, has been retained as permissible to avoid selection bias and ensure adequate representation of the target population, since many constipated PD patients use laxatives occasionally. Laxative use is recorded in the stool diary throughout the trial, and it will be included in the generalized linear mixed models (GLMM) analysis as a time-varying covariate. As participants are matched in pairs, we expect intermittent use to be similarly distributed between groups (Manuscript, page 6; Trial Protocol, updated accordingly). |
| **CURRENT REVIEW**  The eligibility criteria now exclude participants with continuous daily laxative use in the previous eight weeks, which improves clarity. However, the protocol still does not clearly describe how intermittent or rescue laxative use (e.g., osmotic laxatives such as magnesium oxide) will be managed during follow-up and incorporated into the statistical analyses. Additional clarification would strengthen the interpretation of constipation-related outcomes and help distinguish intervention effects from medication-related effects. | **CURRENT ANSWER**  We thank the reviewer for pointing out again the importance of tracking intermittent or rescue laxative use, as it directly impacts the interpretation of the intervention's efficacy. To clarify our protocol's approach: clinically, intermittent or rescue laxative use will not be restricted or managed by the research team during follow-up to ensure patient safety and ethical care. Instead, to isolate the true effect of the dietary intervention from medication-related effects, all intermittent laxative intake will be recorded by the participants in their stool diaries and reviewed in phone calls. These data will then be accounted for in the statistical models. As currently detailed in the "Statistical Analysis" section of the manuscript (page 19). This clarification was included on page 11. |
