# Supplementary material for: Dietitian-led intervention to manage constipation in Parkinson’s disease: Study protocol for a parallel-group randomized controlled trial (NUTRI-GUT-PD)
Source: PLoS One. 2026 Jul 27;21(7):e0354608. doi: 10.1371/journal.pone.0354608 (PMC13405298; doi:10.1371/journal.pone.0354608)
Supplement: Peer review history

# Peer review: Decision Letter 0

**DOI:** 10.1371/journal.pone.0354608.r001

**Authors:** Zehra Batu

31 Mar 2026

PONE-D-26-05353

Dietitian-led Intervention to Manage Constipation in Parkinson’s Disease: Study Protocol for a Parallel-Group Randomized Controlled Trial (NUTRI-GUT-PD)

PLOS One

Dear Dr. OLCHIK,

Thank you for submitting your manuscript to PLOS ONE. After careful consideration, we feel that it has merit but does not fully meet PLOS ONE’s publication criteria as it currently stands. Therefore, we invite you to submit a revised version of the manuscript that addresses the points raised during the review process.

Please submit your revised manuscript by May 15 2026 11:59PM. If you will need more time than this to complete your revisions, please reply to this message or contact the journal office at plosone@plos.org. When you're ready to submit your revision, log on to https://www.editorialmanager.com/pone/ and select the 'Submissions Needing Revision' folder to locate your manuscript file.

Please include the following items when submitting your revised manuscript:

- A letter that responds to each point raised by the academic editor and reviewer(s). You should upload this letter as a separate file labeled 'Response to Reviewers'.
- A marked-up copy of your manuscript that highlights changes made to the original version. You should upload this as a separate file labeled 'Revised Manuscript with Track Changes'.
- An unmarked version of your revised paper without tracked changes. You should upload this as a separate file labeled 'Manuscript'.

If you would like to make changes to your financial disclosure, please include your updated statement in your cover letter. Guidelines for resubmitting your figure files are available below the reviewer comments at the end of this letter.

If applicable, we recommend that you deposit your laboratory protocols in protocols.io to enhance the reproducibility of your results. Protocols.io assigns your protocol its own identifier (DOI) so that it can be cited independently in the future. For instructions see: https://journals.plos.org/plosone/s/submission-guidelines#loc-laboratory-protocols. Additionally, PLOS ONE offers an option for publishing peer-reviewed Lab Protocol articles, which describe protocols hosted on protocols.io. Read more information on sharing protocols at https://plos.org/protocols?utm_medium=editorial-email&utm_source=authorletters&utm_campaign=protocols.

As the corresponding author, your ORCID iD is verified in the submission system and will appear in the published article. PLOS supports the use of ORCID, and we encourage all coauthors to register for an ORCID iD and use it as well. Please encourage your coauthors to verify their ORCID iD within the submission system before final acceptance, as unverified ORCID iDs will not appear in the published article. *Only*  the individual author can complete the verification step; PLOS staff *cannot*  verify ORCID iDs on behalf of authors.

We look forward to receiving your revised manuscript.

Kind regards,

Zehra Batu

Academic Editor

PLOS One

Journal Requirements:

When submitting your revision, we need you to address these additional requirements.

1.Please ensure that your manuscript meets PLOS ONE's style requirements, including those for file naming. The PLOS ONE style templates can be found at

https://journals.plos.org/plosone/s/file?id=wjVg/PLOSOne_formatting_sample_main_body.pdf and

https://journals.plos.org/plosone/s/file?id=ba62/PLOSOne_formatting_sample_title_authors_affiliations.pdf

2. Your ethics statement should only appear in the Methods section of your manuscript. If your ethics statement is written in any section besides the Methods, please delete it from any other section.

3. We note that the Figures in S2_Appendix.pdf in your submission contain copyrighted images. All PLOS content is published under the Creative Commons Attribution License (CC BY 4.0), which means that the manuscript, images, and Supporting Information files will be freely available online, and any third party is permitted to access, download, copy, distribute, and use these materials in any way, even commercially, with proper attribution. For more information, see our copyright guidelines: http://journals.plos.org/plosone/s/licenses-and-copyright.

We require you to either (1) present written permission from the copyright holder to publish these figures specifically under the CC BY 4.0 license, or (2) remove the figures from your submission:

1. You may seek permission from the original copyright holder of the Figures to publish the content specifically under the CC BY 4.0 license.

We recommend that you contact the original copyright holder with the Content Permission Form (http://journals.plos.org/plosone/s/file?id=7c09/content-permission-form.pdf) and the following text:

“I request permission for the open-access journal PLOS ONE to publish XXX under the Creative Commons Attribution License (CCAL) CC BY 4.0 (http://creativecommons.org/licenses/by/4.0/). Please be aware that this license allows unrestricted use and distribution, even commercially, by third parties. Please reply and provide explicit written permission to publish XXX under a CC BY license and complete the attached form.”

Please upload the completed Content Permission Form or other proof of granted permissions as an "Other" file with your submission.

In the figure caption of the copyrighted figure, please include the following text: “Reprinted from [ref] under a CC BY license, with permission from [name of publisher], original copyright [original copyright year].”

2. If you are unable to obtain permission from the original copyright holder to publish these figures under the CC BY 4.0 license or if the copyright holder’s requirements are incompatible with the CC BY 4.0 license, please either i) remove the figure or ii) supply a replacement figure that complies with the CC BY 4.0 license. Please check copyright information on all replacement figures and update the figure caption with source information. If applicable, please specify in the figure caption text when a figure is similar but not identical to the original image and is therefore for illustrative purposes only.

4. Thank you for providing your underlying data as Supporting Information.

We note that the data set contains text or data that is not in English. Please note that PLOS is an English-language publisher, so we require data sets to be provided in English as well. Please upload an English-language version of your data set.

This will also allow us to determine if your data follows PLOS standards per our Data Availability policy here: https://journals.plos.org/plosone/s/data-availability

5. If the reviewer comments include a recommendation to cite specific previously published works, please review and evaluate these publications to determine whether they are relevant and should be cited. There is no requirement to cite these works unless the editor has indicated otherwise.

Additional Editor Comments:

The reviewer comments are below. Please read them carefully and make sure you respond to all suggestions.

[Note: HTML markup is below. Please do not edit.]

Reviewers' comments:

Reviewer's Responses to Questions

**Comments to the Author**

1. Does the manuscript provide a valid rationale for the proposed study, with clearly identified and justified research questions?

The research question outlined is expected to address a valid academic problem or topic and contribute to the base of knowledge in the field.

Reviewer #1: Yes

Reviewer #2: Yes

Reviewer #3: Yes

**********

2. Is the protocol technically sound and planned in a manner that will lead to a meaningful outcome and allow testing the stated hypotheses?

The manuscript should describe the methods in sufficient detail to prevent undisclosed flexibility in the experimental procedure or analysis pipeline, including sufficient outcome-neutral conditions (e.g. necessary controls, absence of floor or ceiling effects) to test the proposed hypotheses and a statistical power analysis where applicable. As there may be aspects of the methodology and analysis which can only be refined once the work is undertaken, authors should outline potential assumptions and explicitly describe what aspects of the proposed analyses, if any, are exploratory.

Reviewer #1: Partly

Reviewer #2: Partly

Reviewer #3: Yes

**********

3. Is the methodology feasible and described in sufficient detail to allow the work to be replicable?

Descriptions of methods and materials in the protocol should be reported in sufficient detail for another researcher to reproduce all experiments and analyses. The protocol should describe the appropriate controls, sample size calculations, and replication needed to ensure that the data are robust and reproducible.

Reviewer #1: Yes

Reviewer #2: No

Reviewer #3: Yes

**********

4. Have the authors described where all data underlying the findings will be made available when the study is complete?

The PLOS Data policy requires authors to make all data underlying the findings described in their manuscript fully available without restriction, with rare exception, at the time of publication. The data should be provided as part of the manuscript or its supporting information, or deposited to a public repository. For example, in addition to summary statistics, the data points behind means, medians and variance measures should be available. If there are restrictions on publicly sharing data—e.g. participant privacy or use of data from a third party—those must be specified.

Reviewer #1: No

Reviewer #2: Yes

Reviewer #3: Yes

**********

5. Is the manuscript presented in an intelligible fashion and written in standard English?

PLOS ONE does not copyedit accepted manuscripts, so the language in submitted articles must be clear, correct, and unambiguous. Any typographical or grammatical errors should be corrected at revision, so please note any specific errors here.

Reviewer #1: Yes

Reviewer #2: Yes

Reviewer #3: Yes

**********

6. Review Comments to the Author

Please use the space provided to explain your answers to the questions above and, if applicable, provide comments about issues authors must address before this protocol can be accepted for publication. You may also include additional comments for the author, including concerns about research or publication ethics.

You may also provide optional suggestions and comments to authors that they might find helpful in planning their study.

(Please upload your review as an attachment if it exceeds 20,000 characters)

Reviewer #1: Reviewer Comments – Major Revision

General Comments:

The manuscript describes a study protocol with important potential contributions to understanding the role of gut microbiota and nutrition in Parkinson’s disease (PD)-related constipation. While the study is generally well designed, several issues need to be addressed to improve clarity, methodological rigor, and reporting consistency before the manuscript can be considered for publication. I recommend major revision.

________________________________________

Specific Comments:

1. Blinding Procedures:

The blinding procedures are described; however, it is not explicitly stated whether the neurologist conducting the study is one of the outcome assessors who are blinded. This should be clarified to ensure methodological transparency.

2. Abstract:

On line 29, replace “study” with “study protocol” to accurately reflect the manuscript type.

3. Introduction:

• The introduction should be strengthened with more compelling statements regarding gut microbiota and constipation in Parkinson’s disease.

• Literature on PD-gut microbiota should be better contextualized, particularly human microbiome studies, to justify the planned 16S rRNA analysis.

• Line 74 references study 13, which demonstrated the effectiveness of the Mediterranean diet; the sentence should be revised to accurately reflect the study findings.

• Lines 75–76 reference study 14, which observed no changes in colonic structure; this point should also be addressed.

• The final paragraph should clearly state the primary objective and, if applicable, explicitly define primary and secondary hypotheses.

4. Eligibility Criteria:

• The protocol should clarify how continuous or intermittent use of osmotic laxatives (e.g., magnesium oxide) will be handled in terms of eligibility, follow-up, and statistical analysis.

• Since the intervention relies on individualized food-based counseling, the authors should clarify whether participants requiring texture-modified diets, feeding assistance, or having relevant chewing limitations are eligible, and how adherence will be ensured in such cases.

5. Data Collection:

• Physical activity records should be monitored at the midpoint as well, or the analysis should explicitly consider them as potential confounders.

• The term “Three 24-hour Dietary Recalls” is insufficiently precise. It should be clarified whether dietary intake will be recorded for a single day per session or over three days. Consistent terminology should be used throughout the manuscript (e.g., “A 3-day diet diary [two weekdays and one weekend day] was used to assess dietary intake” or “24-hour dietary recall was used to assess dietary intake”). These records can then be used to calculate a diet quality index.

• Using both food frequency assessment and the Screener-Nova is unnecessary. Evaluating food frequency at baseline and study end may be more appropriate.

• For anthropometric measures, calf circumference may not be required since it is already included in the MNA short form. Similarly, arm circumference may not be necessary because BIA and muscle strength measurements are performed; hip circumference can be preferred instead.

• In Figure 1, “nutritional risk screening” can be replaced with “MNA-SF” directly for clarity.

6. References:

Recent references should be included where available to support background on PD constipation, microbiota, and nutrition interventions, while retaining seminal methodological references.

Reviewer #2: • Defining the primary outcome with a single, clear measurement

• Updating the statistical analysis plan with an appropriate model for the census data

• Presenting the sample size calculation along with its sources

• Standardizing and defining the content of the control group

• Clarifying the number of visits consistent with the SPIRIT scheme

• Discussing the risk of bias due to the absence of participant blindness

• Updating the missing data management with modern standards and addressing the deficiencies noted in the reviewer file.

Reviewer #3: The authors present the protocol for a randomized controlled trial assessing the efficacy of a nutritional intervention on constipation outcomes among individuals with Parkinson's disease and constipation. The manuscript will be strengthened if the authors consider the following points.

1. Authors are encouraged to include a brief description of the instruments being used as part of the neurological evaluation rather than just listing them with corresponding references. Also, there appears to be a couple of instruments mentioned in the protocol that are not in the manuscript (ANNEX-D, BDI). The protocol and manuscript should be consistent.

2. The primary outcome is based on a change in weekly bowel movements, as captured by the stool diary and the Constipation Scoring System (CSS). The stool diary is completed daily so weekly measures are obtained over the entire 90-day duration of the trial. The CSS is measured at 3 different time points (baseline, day 45 and day 90). However, in the statistical analysis section, authors mention the analysis of weekly bowel movements will be done using a t-test. Other outcomes (that are also collected at 2-3 time points) are analyzed with generalized linear mixed models due to the repeated measures. It is not clear why the weekly bowel movements will be analyzed with a t-test, especially when weekly data over 3 months will be available. The protocol, included as part of the supplemental material, also does not indicate the t-test as the method being used to analyze data.

3. There is an inconsistency in the manuscript for the handling of missing data. In line 369, authors indicate they will use last-observation carried forward and later in line 389, they indicate they will use multiple imputation. Authors should clarify when each approach will be applied.

Minor points:

1. line 145: BIA should be defined here (it is defined in Figure captions and in line 275), but this is the first use of the acronym

2. line 162: I believe the year should be 2026 instead of November 2027.

3. lines 186-187: please make this a complete sentence

4. line 255: authors mention a nutrient analysis software - is there a specific one being used?

**********

7. PLOS authors have the option to publish the peer review history of their article (what does this mean?). If published, this will include your full peer review and any attached files.

If you choose “no”, your identity will remain anonymous but your review may still be made public.

**Do you want your identity to be public for this peer review?**  For information about this choice, including consent withdrawal, please see our Privacy Policy.

Reviewer #1: No

Reviewer #2: **Yes:** Assistant Professor Basak CAN

Reviewer #3: No

**********

[NOTE: If reviewer comments were submitted as an attachment file, they will be attached to this email and accessible via the submission site. Please log into your account, locate the manuscript record, and check for the action link "View Attachments". If this link does not appear, there are no attachment files.]

To ensure your figures meet our technical requirements, please review our figure guidelines: https://journals.plos.org/plosone/s/figures

You may also use PLOS’s free figure tool, NAAS, to help you prepare publication quality figures: https://journals.plos.org/plosone/s/figures#loc-tools-for-figure-preparation.

NAAS will assess whether your figures meet our technical requirements by comparing each figure against our figure specifications.

Attachment Submitted filename: PONE-D-26-05353_reviewer.docx

Attachment Submitted filename: PONE-D-26-05353_reviewer (2).pdf

# Author comment: Author response to Decision Letter 1

**DOI:** 10.1371/journal.pone.0354608.r002

25 May 2026

Dear Editor and Reviewers,

We would like to express our sincere gratitude to the Editor and all three Reviewers for the thorough and thoughtful evaluation of our manuscript. We greatly appreciate the comments and suggestions provided during the review process. We carefully revised the manuscript, considering all recommendations, which contributed to improving the clarity, methodological rigor, and overall quality of the study protocol.

Among the main revisions, the statistical analysis section was revised and clarified, and the intervention protocol was described in greater detail to improve reproducibility and methodological transparency. In addition, all minor comments and suggestions provided by the reviewers were carefully addressed throughout the manuscript, and all changes in the revised manuscript have been highlighted.

To facilitate the review of the new version, we prepared the point-by-point response document "Response to Reviewers" specifying the location of each modification (document and page number).

We sincerely hope that the revised version satisfactorily addresses all concerns raised, and we remain available for any further clarification.

Attachment Submitted filename: Response to Reviewers.docx

# Peer review: Decision Letter 1

**DOI:** 10.1371/journal.pone.0354608.r003

**Authors:** Zehra Batu

16 Jun 2026

PONE-D-26-05353R1

Dietitian-led Intervention to Manage Constipation in Parkinson’s Disease: Study Protocol for a Parallel-Group Randomized Controlled Trial (NUTRI-GUT-PD)

PLOS One

Dear Dr. OLCHIK,

Thank you for submitting your manuscript to PLOS ONE. After careful consideration, we feel that it has merit but does not fully meet PLOS ONE’s publication criteria as it currently stands. Therefore, we invite you to submit a revised version of the manuscript that addresses the points raised during the review process.

- **Please review each of Reviewer 1's questions, make any necessary adjustments, and ensure you answer them all.**

-->Please submit your revised manuscript by Jul 31 2026 11:59PM. If you will need more time than this to complete your revisions, please reply to this message or contact the journal office at plosone@plos.org. When you're ready to submit your revision, log on to https://www.editorialmanager.com/pone/ and select the 'Submissions Needing Revision' folder to locate your manuscript file.

Please include the following items when submitting your revised manuscript:

- A letter that responds to each point raised by the academic editor and reviewer(s). You should upload this letter as a separate file labeled 'Response to Reviewers'.
- A marked-up copy of your manuscript that highlights changes made to the original version. You should upload this as a separate file labeled 'Revised Manuscript with Track Changes'.
- An unmarked version of your revised paper without tracked changes. You should upload this as a separate file labeled 'Manuscript'.

If you would like to make changes to your financial disclosure, please include your updated statement in your cover letter. Guidelines for resubmitting your figure files are available below the reviewer comments at the end of this letter.

If applicable, we recommend that you deposit your laboratory protocols in protocols.io to enhance the reproducibility of your results. Protocols.io assigns your protocol its own identifier (DOI) so that it can be cited independently in the future. For instructions see: https://journals.plos.org/plosone/s/submission-guidelines#loc-laboratory-protocols. Additionally, PLOS ONE offers an option for publishing peer-reviewed Lab Protocol articles, which describe protocols hosted on protocols.io. Read more information on sharing protocols at https://plos.org/protocols?utm_medium=editorial-email&utm_source=authorletters&utm_campaign=protocols.

As the corresponding author, your ORCID iD is verified in the submission system and will appear in the published article. PLOS supports the use of ORCID, and we encourage all coauthors to register for an ORCID iD and use it as well. Please encourage your coauthors to verify their ORCID iD within the submission system before final acceptance, as unverified ORCID iDs will not appear in the published article. *Only*  the individual author can complete the verification step; PLOS staff *cannot*  verify ORCID iDs on behalf of authors.

We look forward to receiving your revised manuscript.

Kind regards,

Zehra Batu

Academic Editor

PLOS One

**Journal Requirements:**

1. If the reviewer comments include a recommendation to cite specific previously published works, please review and evaluate these publications to determine whether they are relevant and should be cited. There is no requirement to cite these works unless the editor has indicated otherwise.

2. Please review your reference list to ensure that it is complete and correct. If you have cited papers that have been retracted, please include the rationale for doing so in the manuscript text, or remove these references and replace them with relevant current references. Any changes to the reference list should be mentioned in the rebuttal letter that accompanies your revised manuscript. If you need to cite a retracted article, indicate the article’s retracted status in the References list and also include a citation and full reference for the retraction notice.

[Note: HTML markup is below. Please do not edit.]

Reviewers' comments:

Reviewer's Responses to Questions

**Comments to the Author**

1. Does the manuscript provide a valid rationale for the proposed study, with clearly identified and justified research questions?

The research question outlined is expected to address a valid academic problem or topic and contribute to the base of knowledge in the field.

Reviewer #1: Yes

Reviewer #3: Yes

**********

2. Is the protocol technically sound and planned in a manner that will lead to a meaningful outcome and allow testing the stated hypotheses?

The manuscript should describe the methods in sufficient detail to prevent undisclosed flexibility in the experimental procedure or analysis pipeline, including sufficient outcome-neutral conditions (e.g. necessary controls, absence of floor or ceiling effects) to test the proposed hypotheses and a statistical power analysis where applicable. As there may be aspects of the methodology and analysis which can only be refined once the work is undertaken, authors should outline potential assumptions and explicitly describe what aspects of the proposed analyses, if any, are exploratory.

Reviewer #1: Yes

Reviewer #3: Yes

**********

3. Is the methodology feasible and described in sufficient detail to allow the work to be replicable?

Descriptions of methods and materials in the protocol should be reported in sufficient detail for another researcher to reproduce all experiments and analyses. The protocol should describe the appropriate controls, sample size calculations, and replication needed to ensure that the data are robust and reproducible.

Reviewer #1: Yes

Reviewer #3: Yes

**********

4. Have the authors described where all data underlying the findings will be made available when the study is complete?

The PLOS Data policy requires authors to make all data underlying the findings described in their manuscript fully available without restriction, with rare exception, at the time of publication. The data should be provided as part of the manuscript or its supporting information, or deposited to a public repository. For example, in addition to summary statistics, the data points behind means, medians and variance measures should be available. If there are restrictions on publicly sharing data—e.g. participant privacy or use of data from a third party—those must be specified.

Reviewer #1: Yes

Reviewer #3: Yes

**********

5. Is the manuscript presented in an intelligible fashion and written in standard English?

PLOS ONE does not copyedit accepted manuscripts, so the language in submitted articles must be clear, correct, and unambiguous. Any typographical or grammatical errors should be corrected at revision, so please note any specific errors here.

Reviewer #1: Yes

Reviewer #3: Yes

**********

6. Review Comments to the Author

Please use the space provided to explain your answers to the questions above and, if applicable, provide comments about issues authors must address before this protocol can be accepted for publication. You may also include additional comments for the author, including concerns about research or publication ethics.

You may also provide optional suggestions and comments to authors that they might find helpful in planning their study.

(Please upload your review as an attachment if it exceeds 20,000 characters)

**Reviewer #1:** Although the manuscript states that outcome assessors, microbiota sequencing personnel, and the statistician will be blinded to group allocation, it remains unclear whether the study neurologist performing the clinical assessments (e.g., MDS-UPDRS, MoCA, SDQ, and IPAQ) is also blinded. Since these assessments contribute to secondary outcomes, please clarify whether the neurologist is considered an outcome assessor and, if so, whether blinding will be maintained throughout the study.

The clarification regarding dietary assessments is appreciated. However, physical activity remains a potential confounder in constipation outcomes. The IPAQ-SF appears to be administered only at baseline and D90. Please either justify why physical activity is not assessed at the midpoint (D45) or explicitly indicate how changes in physical activity during the intervention period will be accounted for in the statistical analyses.

The eligibility criteria now exclude participants with continuous daily laxative use in the previous eight weeks, which improves clarity. However, the protocol still does not clearly describe how intermittent or rescue laxative use (e.g., osmotic laxatives such as magnesium oxide) will be managed during follow-up and incorporated into the statistical analyses. Additional clarification would strengthen the interpretation of constipation-related outcomes and help distinguish intervention effects from medication-related effects.

**Reviewer #3:** The authors have adequately addressed my earlier comments. I have no remaining critiques and am satisfied with the current version.

**********

7. PLOS authors have the option to publish the peer review history of their article (what does this mean?). If published, this will include your full peer review and any attached files.

If you choose “no”, your identity will remain anonymous but your review may still be made public.

**Do you want your identity to be public for this peer review?**  For information about this choice, including consent withdrawal, please see our Privacy Policy.

Reviewer #1: **Yes:** hülya oğur

Reviewer #3: No

**********

[NOTE: If reviewer comments were submitted as an attachment file, they will be attached to this email and accessible via the submission site. Please log into your account, locate the manuscript record, and check for the action link "View Attachments". If this link does not appear, there are no attachment files.]

To ensure your figures meet our technical requirements, please review our figure guidelines: https://journals.plos.org/plosone/s/figures

You may also use PLOS’s free figure tool, NAAS, to help you prepare publication quality figures: https://journals.plos.org/plosone/s/figures#loc-tools-for-figure-preparation.

NAAS will assess whether your figures meet our technical requirements by comparing each figure against our figure specifications.

# Author comment: Author response to Decision Letter 2

**DOI:** 10.1371/journal.pone.0354608.r004

30 Jun 2026

We sincerely thank Reviewer 1 for the comments. In the "Response to reviwers file", we provide our detailed point-by-point responses to each comment. We carefully revisited every question and thoroughly reviewed the manuscript to ensure that all concerns were comprehensively addressed and that the corresponding revisions were clearly presented.

We noted that several comments refer to issues raised during the initial review and addressed in our previous revision. Nevertheless, to facilitate the review process and avoid any ambiguity, we have provided additional explanations where appropriate and further refined the manuscript. These revisions have improved the clarity, transparency, and overall presentation of the manuscript. All changes have been highlighted in the revised version, and the corresponding sections are identified in the file.

Attachment Submitted filename: Response_to_Reviewers_auresp_2.docx

# Editor report: Decision Letter 2

**DOI:** 10.1371/journal.pone.0354608.r005

**Authors:** Zehra Batu

9 Jul 2026

Dietitian-led Intervention to Manage Constipation in Parkinson’s Disease: Study Protocol for a Parallel-Group Randomized Controlled Trial (NUTRI-GUT-PD)

PONE-D-26-05353R2

Dear Dr. MAIRA ROZENFELD OLCHIK

We’re pleased to inform you that your manuscript has been judged scientifically suitable for publication and will be formally accepted for publication once it meets all outstanding technical requirements.

Within one week, you’ll receive an e-mail detailing the required amendments. When these have been addressed, you’ll receive a formal acceptance letter and your manuscript will be scheduled for publication.

An invoice will be generated when your article is formally accepted. Please note, if your institution has a publishing partnership with PLOS and your article meets the relevant criteria, all or part of your publication costs will be covered. Please make sure your user information is up-to-date by logging into Editorial Manager at Editorial Manager® and clicking the ‘Update My Information' link at the top of the page. For questions related to billing, please contact billing support.

If your institution or institutions have a press office, please notify them about your upcoming paper to help maximize its impact. If they’ll be preparing press materials, please inform our press team as soon as possible -- no later than 48 hours after receiving the formal acceptance. Your manuscript will remain under strict press embargo until 2 pm Eastern Time on the date of publication. For more information, please contact onepress@plos.org.

Kind regards,

Zehra Batu

Academic Editor

PLOS One

Additional Editor Comments (optional):

Reviewers' comments:

# Editor report: Acceptance letter

**DOI:** 10.1371/journal.pone.0354608.r006

**Authors:** Zehra Batu

PONE-D-26-05353R2

PLOS One

Dear Dr. OLCHIK,

I'm pleased to inform you that your manuscript has been deemed suitable for publication in PLOS One. Congratulations! Your manuscript is now being handed over to our production team.

At this stage, our production department will prepare your paper for publication. This includes ensuring the following:

* All references, tables, and figures are properly cited

* All relevant supporting information is included in the manuscript submission,

* There are no issues that prevent the paper from being properly typeset

You will receive further instructions from the production team, including instructions on how to review your proof when it is ready. Please keep in mind that we are working through a large volume of accepted articles, so please give us a few days to review your paper and let you know the next and final steps.

Lastly, if your institution or institutions have a press office, please let them know about your upcoming paper now to help maximize its impact. If they'll be preparing press materials, please inform our press team within the next 48 hours. Your manuscript will remain under strict press embargo until 2 pm Eastern Time on the date of publication. For more information, please contact onepress@plos.org.

You will receive an invoice from PLOS for your publication fee after your manuscript has reached the completed accept phase. If you receive an email requesting payment before acceptance or for any other service, this may be a phishing scheme. Learn how to identify phishing emails and protect your accounts at https://explore.plos.org/phishing.

If we can help with anything else, please email us at customercare@plos.org.

Thank you for submitting your work to PLOS ONE and supporting open access.

Kind regards,

PLOS ONE Editorial Office Staff

on behalf of

Professor Zehra Batu

Academic Editor

PLOS One
